# Anti-Obesity Evaluation of *Averrhoa carambola* L. Leaves and Assessment of Its Polyphenols as Potential α-Glucosidase Inhibitors

**DOI:** 10.3390/molecules27165159

**Published:** 2022-08-12

**Authors:** Nehal S. Ramadan, Nabil H. El-Sayed, Sayed A. El-Toumy, Doha Abdou Mohamed, Zeinab Abdel Aziz, Mohamed Sobhy Marzouk, Tuba Esatbeyoglu, Mohamed A. Farag, Kuniyoshi Shimizu

**Affiliations:** 1 Chemistry of Tanning Materials and Leather Technology Department, National Research Centre, Dokki, Cairo 12622, Egypt; 2 Nutrition and Food Sciences Department, National Research Centre, Dokki, Cairo 12622, Egypt; 3 Pharmacognosy Department, College of Pharmacy, Cairo University, Kasr El Aini St., Cairo 11562, Egypt; 4 Department of Food Development and Food Quality, Institute of Food Science and Human Nutrition, Gottfried Wilhelm Leibniz University Hannover, Am Kleinen Felde 30, 30167 Hannover, Germany; 5 Department of Agro-Environmental Sciences, Graduate School of Bioresource and Bioenvironmental Sciences, Kyushu University, Fukuoka 819-0395, Japan

**Keywords:** antidiabetic, *Averrhoa* *carambola* L., dihydrochalcone, flavone glycosides, obesity, Oxalidaceae, type 2 diabetes

## Abstract

*Averrhoa carambola* L. is reported for its anti-obese and anti-diabetic activities. The present study aimed to investigate its aqueous methanol leaf extract (CLL) in vivo anti-obese activity along with the isolation and identification of bioactive compounds and their in vitro α-glucosidase inhibition assessment. CLL improved all obesity complications and exhibited significant activity in an obese rat model. Fourteen compounds, including four flavone glycosides (**1**–**4**) and ten dihydrochalcone glycosides (**5**–**12**), were isolated and identified using spectroscopic techniques. New compounds identified in planta included (**1**) apigenin 6-*C*-(2-deoxy-*β*-D-galactopyranoside)-7-*O*-*β*-D-quinovopyranoside, (**8**) phloretin 3′-*C*-(2-*O*-(*E*)-cinnamoyl-3-*O*-*β*-D-fucopyranosyl-4-*O*-acetyl)-*β*-D-fucopyranosyl-6′-*O*-*β*-D fucopyranosyl-(1/2)-α-L arabinofuranoside, (**11a**) phloretin3′-*C*-(2-*O*-(*E*)-p-coumaroyl-3-*O*-*β-*D-fucosyl-4-*O*-acetyl)-*β*-D-fucosyl-6′-*O*-(2-*O*-*β*-D-fucosyl)-α-L-arabinofuranoside, (**11b**) phloretin3′-*C*-(2-O-*(Z)-p*-coumaroyl-3-*O*-*β*-D-fucosyl-4-*O*-acetyl)-*β*-D-fucosyl-6′-*O*-(2-*O*-*β*-D-fucosyl)-α-L-arabinofuranoside. Carambolaside M (**5**), carambolaside Ia (**6**), carambolaside J (**7**), carambolaside I (**9**), carambolaside P (**10a**), carambolaside O (**10b**), and carambolaside Q (**12**), which are reported for the first time from *A. carambola* L. leaves, whereas luteolin 6-*C*-α-L-rhamnopyranosyl-(1-2)-*β*-D-fucopyranoside (**2**), apigenin 6-*C*-*β*-D-galactopyranoside (**3**), and apigenin 6-*C*-*α*-L-rhamnopyranosyl-(1-2)-*β*-L-fucopyranoside (**4**) are isolated for the first time from Family. Oxalidaceae. In vitro α-glucosidase inhibitory activity revealed the potential efficacy of flavone glycosides, viz., **1**, **2**, **3**, and **4** as antidiabetic agents. In contrast, dihydrochalcone glycosides (**5**–**11**) showed weak activity, except for compound **12**, which showed relatively strong activity.

## 1. Introduction

The radical shift from malnutrition to overnutrition, as well as the increase in sedentary behaviour, has led to the increasing incidence of obesity, a complex chronic nutritional disorder characterized by an energy expenditure and intake imbalance. With estimates of 2.3 billion overweight individuals and 700 million obese adults, obesity with its comorbidities is considered the fifth-largest cause of death worldwide [1,2]. Insulin resistance is one of the most prevalent obesity-related changes [3] and hence, obesity is a key predisposal to type 2 diabetes [1]. Furthermore, some white fat storage areas in the body are more directly associated to metabolic consequences of obesity, such as diabetes, than others [2]. Obesity-related problems, viz., diabetes has been associated with decreased life expectancy [4] as well as imparting various clinical disorders such as renal disease, blindness, and amputation of lower limbs, among others [5].

Moreover, obesity and associated symptoms are predicted to cost the global economy USD 2 trillion per year nearly as much as smoking, armed conflict, and terrorism [4]. The common synthetic anti-obesity medicine orlistat is successful in treating obesity, however, it exerts serious gastrointestinal side effects [6].

Likewise, α-glucosidase inhibitors as typical anti-diabetic medicines reported to possess side effects despite their important function in lowering blood glucose levels [7]. Only three α-glucosidase inhibitors are currently used in clinical practice including acarbose, miglitol, and voglibose [5,7], warranting for the development of natural medicines that comprise medicinal herbs, either as pure components or as extracts, as an alternative therapy for obesity [3,7].

For decades, *Averrhoa carambola* L., commonly known as starfruit, a member of the Oxalidaceae family, indigenous to the tropical southeast, is planted across the tropics for its edible fruit as well as its ornamental traits, and it was recently domesticated in other countries, including Ecuador and Egypt [8]. *A. carambola* L. flesh is reported for its potential in the treatment of diabetes [9] as well as its confirmed hypoglycemic and porcine pancreatic lipase inhibitory effects [10,11].

In spite of being edible with several health benefits, starfruit is contraindicated in uremic patients owing to its high oxalate content in addition to its negative inotropic and chronotropic effects [8].

Besides, *A. carambola* leaves were reported for their traditional uses in treatment of hyperglycemia, diabetes, and its related diseases [12,13]. Biological studies further confirmed the hypoglycemic activities of leaves and some of its isolated compounds [13,14] Moreover, leaves were reported to possess potential antioxidant activity [15]. Further, leaf decoction are reported to be used for treatment of aphthous stomatitis and angina [16].

With regards to chemical composition and compared to fruits, *A. carambola* leaves are less investigated. Flavone *C*-glycosides have been previously isolated from leaves, viz., isovitexin, carambolaflavones A and B, and apigenin 6-*C*-(2″-O-α-L-rhamnopyranosyl)-*β*-D-glucopyranoside [15]. Both carambolaflavones were reported for their hypoglycemic effect in rats [12]. Recently, 12 dihydrochalcone *C*-glycosides were reported from leaves [17]. Dihydrochalcones are natural phenolics with a C6–C3–C6 skeleton structure, where two aromatic rings are connected via a C3 chain [18] and abundant in *A. carambola*.

In the context of the overall strategy to control obesity and its complications using functional foods, an *A. carambola* crude methanol-leaf extract (CLL) anti-obese effect was assessed using an in vivo high fat diet (HFD)-induced obesity rat model. CLL extract as well as Orly (as reference drug) were orally administered as interventions for the management of obesity showing significant improvement in obesity and its associated complications.

Where, rats were fed on high fat diet for eight weeks resulting in dyslipidemia, hyperglycemia, hyperleptinemia, insulin resistance, oxidative stress, and abnormalities in liver and kidney functions. To confirm development of the obesity model and to assess different treatment actions, both physical and biochemical parameters were monitored. Further, leaf extract was subjected to detailed phytochemical isolation to identify active agent(s) using NMR and MS spectroscopy, with pure compounds assessed for their α-glucosidase inhibitory activity.

## 2. Results and Discussion

### 2.1. In Vivo Assay of A. carambola Leaf Extract against HFD-Induced Obesity Model in Rats

*A. carambola* L. flesh has been reported for the treatment of diabetes in folk medicine [9]. Besides, pharmacological assays confirmed its hypoglycemic effect [10] as well as its porcine pancreatic lipase inhibitory effect [11]. Starfruit is known for its richness in phenolics, especially flavonoids [19], its potential for preventing and curing metabolic disorders, viz., obesity and obesity-related metabolic syndrome [20]. The existence of bioactive phytochemicals, i.e., flavan-3-ols and 2-diglycosyloxybenzoates in carambola leaf, with reported lipase and α-glucosidase inhibitory activities [17], might participate in the anti-obese activity of leaves, warranting their assessment. The effect of CLL extract was assessed against different parameters in HFD-induced obese rats including body weight, dyslipidemia, effect on leptin, α-amylase, plasma glucose, insulin levels and insulin resistance, oxidative stress, and lipid peroxidation as well as the effect on liver and kidney functions, as detailed in the next subsections.

#### 2.1.1. Body Weight and Biochemical Markers Determination

**Figure 1:**
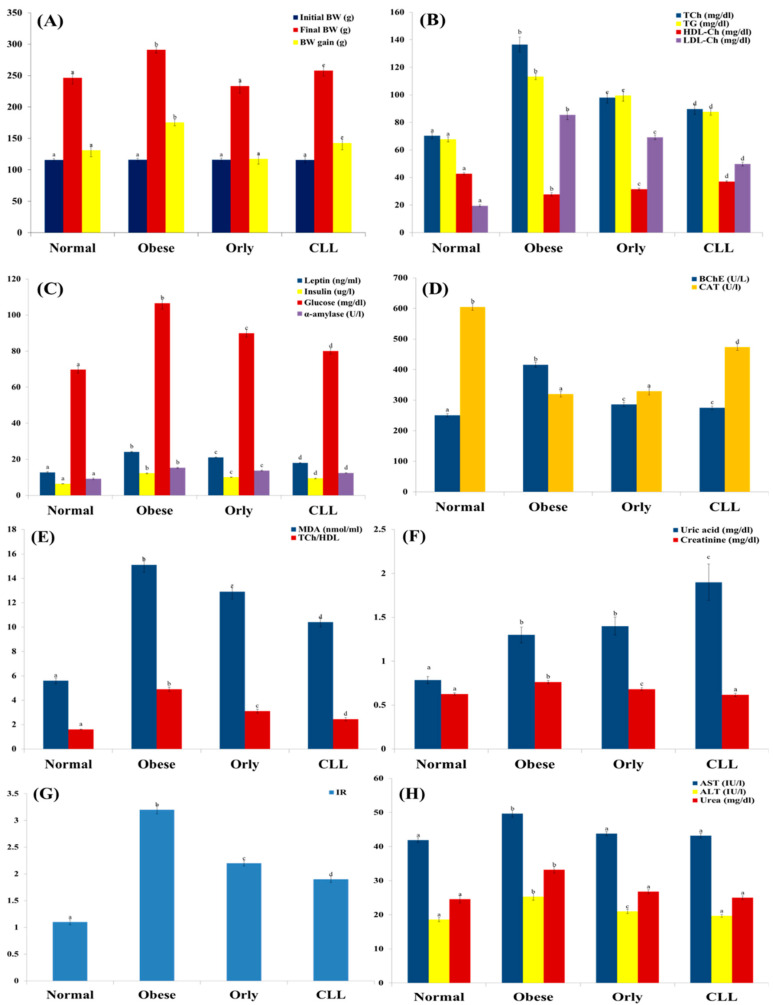
Body weight and biochemical parameters in the 4th week of treatment of experimental animals with Orly and CLL compared to normal and obese groups. (**A**) The initial, final body weights (BW) and BW gain (g). (**B**) The level of plasma total cholesterol (TCh mg/dL), triglycerides (TG mg/dL), high-density lipoprotein cholesterol (HDL-Ch mg/dL), and low-density lipoprotein cholesterol (LDL-Ch mg/dL). (**C**) The level of leptin (ng/mL), insulin (μg/L), glucose (mg/dL), and α-amylase (U/L). (**D**) The level of plasma butyrylcholinesterase (BChE (U/L)) and plasma catalase activity (CAT (U/L)). (**E**) The level of malondialdehyde (MDA (nmol/mL) and TCh/HDL. (**F**) The level of plasma uric acid (mg/dL) and plasma creatinine (mg/dL). (**G**) Calculated insulin resistance (IR). (**H**) The activity of aspartate transaminase (AST (IU/L)), alanine transaminase (ALT (IU/L)), and urea (mg/dL). Each bar graph represents the mean replicate measurement (*n* = 6) expressed as mean ± S.E. The bar graphs with a similar lower-case letter (such as ‘a’) among experimental groups are not significantly different from each other (*p* > 0.05). The bar graphs with different lower-case letters (such as a, b, c, d, and e) are statistically different from each other (*p* < 0.05).

CLL extracts showed a significant reduction in rat body weight (258 g) compared to obese rats (291 g), however, it was still higher than the normal rats group (246 g) (Figure 1A and Table 1). Although Orly caused a significant decrease in BW gain (*p* < 0.05), as compared with the CLL and obese groups, several biochemical markers were measured as the index for obesity status, revealing the excelling of CLL over Orly, as detailed in the next subsections.

#### 2.1.2. Effect of CLL on Dyslipidemia

Dyslipidemia, a metabolic complication of obesity manifested by hypertriglyceridemia [21], was observed in obese rats compared to normal rats, as shown by the elevation of plasma total cholesterol (two-fold increase), triglycerides (1.7-fold increase), LDL cholesterol (four-fold increase), and the ratio of T-Ch/HDL-Ch (three-fold increase) (Figure 1A, Table 1) concurrent with the reduction in plasma level of HDL-Ch (1.5-fold decrease) (Figure 1E, Table 1). CLL significantly improved dyslipidemia compared to obese control rats as well as rats treated with Orly (*p* < 0.05), however, it was still higher than normal rats (Figure 1A,E, Table 1).

#### 2.1.3. Effect of CLL on Leptin, α-Amylase, Plasma Glucose, Insulin Levels, and Insulin Resistance

Obese rats are also reported to exhibit increment in the plasma levels of plasma glucose, insulin, insulin resistance, leptin, and α-amylase [22]. A significant elevation in plasma levels of glucose (1.5-fold increase), insulin (two-fold increase), and insulin resistance (three-fold increase) was noted in obese rats compared to normal rats.

Oral administration of Orly and CLL improved plasma levels of glucose (89.9 and 80 mg/dL, respectively), insulin (10.1 and 9.4 µg/L, respectively), and insulin resistance (2.2 and 1.9, respectively) with different degrees (Figure 1C,G, Table 1). Obese rats exhibited significantly elevated levels of plasma leptin, a key hormone in the control of food intake and body weight and a target in obesity management [23,24] (two-fold increase) comparable to those in normal rats. Orly and CLL significantly reduced plasma levels of leptin (21.1 and 18.0 ng/mL, respectively) compared to obese control (24.1 ng/mL). Another drug target in obesity is the inhibition of the digestive enzyme α-amylase [25]. In this study, significant increase in α-amylase activity in obese rats (15.3 U/L) was dramatically reduced upon administration of both Orly and CLL (13.7 and 12.4 U/L, respectively) (Figure 1C, Table 1). Hence, CLL is significantly excelling over Orly in decreasing leptin, insulin, glucose, and α-amylase levels (*p* < 0.05).

#### 2.1.4. Effect of CLL on Oxidative Stress and Lipid Peroxidation

In the current study, elevated plasma levels of butyrylcholinesterase (BChE) were observed in the obese control (415.7 U/L) in agreement with [22], compared to different experimental groups (250.7, 274.8, and 285.2 U/L in normal, CLL and Orly treated groups, respectively). High plasma BChE activity is associated with aberrant lipid profiles, insulin resistance, and hypertension [23], suggestive for BChE role in many metabolic functions [24]. Oral administration of Orly as well as CLL reduced BchE plasma elevation significantly at different levels (285.2 and 274.8 U/L, respectively). Malondialdehyde (MDA), a biomarker used for assessing oxidative stress, was significantly enhanced in obese rats (15.1 nmol/mL) compared to those of normal ones (5.6 nmol/mL), as an indicator of lipid peroxidation, while catalase enzyme activity, an indicator of antioxidant status, showed reduction by 1.9 fold. Rats treated with Orly and CLL exhibited improved oxidative stress markers at different levels (Figure 1D,E, Table 1). CLL revealed significant improvement in oxidative stress markers and lipid peroxidation profiles, better than Orly (*p* < 0.05).

#### 2.1.5. Effect of CLL on Kidney and Liver Functions

Kidney function indicators (creatinine, urea, and uric acid) as well as plasma transaminases (AST and ALT) revealed significant elevation in obese rats compared to normal rats, in agreement with [25]. Treatment with Orly and CLL significantly improved kidney and liver functions, except for the significant elevation of uric acid content in the case of CLL, which is most probably attributed to the high oxalate level in the leaves [26] (Figure 1F,H, Table 1). Hence, CLL was significantly better than Orly in terms of kidney- and liver-function improvement, except for an elevated uric acid level (*p* < 0.05).

Overall, despite the better effect of Orly in reducing body weight gain compared to CLL, the latter revealed better improvement in mostly all tested biochemical parameters, except for an elevated uric acid level.

### 2.2. Isolation and Structure Elucidation

To identify anti-obese agents in the CLL extract, the extract was subjected to fractionation using column chromatography (CC) and liquid chromatography (LC), to afford 14 compounds (C1–C12) including 4 flavone glycosides, i.e., **1** (Figures S1–S6), **2** (Figures S7–S11), **3** (Figures S12–S14), and **4** (Figures S15–S19) as well as 10 dihydrochalcone glycosides, i.e., **5** (Figures S20–S24), **6** (Figures S25–S29), **7** (Figures S30–S36), **8** (Figures S37–S43), **9** (Figures S44–S48), **10a** and **10b** (Figures S49–S54), **11a** and **11b** (Figures S55–S60), and **12** (Figures S61–S67). All compounds were checked for their purity using HPLC (Figure S68).

#### Isolated Compounds Structure Determination Using NMR and MS

Fourteen compounds were isolated and identified using different spectroscopic techniques including (**1**) apigenin 6-*C*-(2-deoxy-*β*-D-galactopyranoside)-7-*O*-*β*-D-quinovopyranoside, (**2**) luteolin 6-*C*-α-L-rhamnopyranosyl-(1-2)-*β*-D-fucopyranoside, (**3**) apigenin 6-*C*-*β*-D-galactopyranoside, (**4**) apigenin 6-*C*-*α*-L-rhamnopyranosyl-(1-2)-*β*-L-fucopyranoside, (**5**) carambolaside M, (**6**) carambolaside Ia, (**7**) carambolaside J, (**8**) phloretin 3′-*C*-(2-*O*-(*E*)-cinnamoyl-3-*O*-*β*-D-fucopyranosyl-4-*O*-acetyl)-*β*-D-fucopyranosyl-6′-*O*-*β*-D fucopyranosyl-(1/2)-α-L arabinofuranoside, (**9**) carambolaside I, (**10a**) carambolaside P, (**10b**) carambolaside O, (**11a**) phloretin3′-*C*-(2-*O*-(*E*)-p-coumaroyl-3-*O*-*β-*D-fucosyl-4-*O*-acetyl)-*β*-D-fucosyl-6′-*O*-(2-*O*-*β*-D-fucosyl)-α-L-arabinofuranoside, (**11b**) phloretin3′-*C*-(2-*O*-(*E*)-p-coumaroyl-3-*O*-*β-*D-fucosyl-4-*O*-acetyl)-*β*-D-fucosyl-6′-*O*-(2-*O*-*β*-D-fucosyl)-α-L-arabinofuranoside, and (**12**) carambolaside Q.

**Figure 2:**
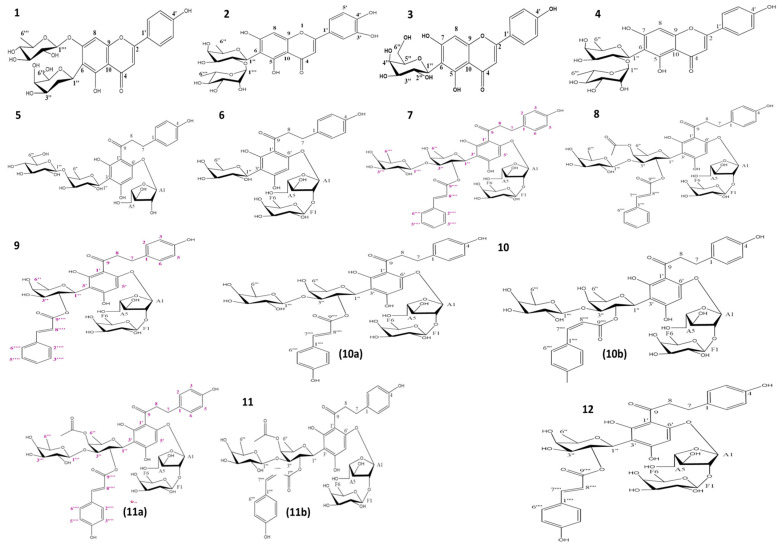
Chemical structures of compounds **1**–**12** isolated from CLL extract.

New compounds for the first time to be identified *in nature*, including compounds **1**, **8**, **11a**, and **11b**, are discussed in detail in this section. All spectral data are provided in supplementary file. Compound **1** (Figure 2) was isolated as a yellowish amorphous powder soluble in 100% MeOH. The molecular formula of compound **1** was calculated as C_27_H_30_O_13_, based on a deprotonated ion peak calculated at *m*/*z* 561.16137, detected at *m*/*z* 561.1614 [M-H]^−^ (calculated C_27_H_29_O_13_^−^, error −0.1 ppm) in the HR-ESI-MS spectrum (Figure S1). Compound 1 showed two UV maximums (λ_max_) (MeOH) at 270 nm (Band II) and 334 nm (Band I), characteristic for a flavone skeleton [27]. The IR spectrum of compound 1 illustrated a broad band at 3431.4 cm^−1^ and 1623 cm^−1^, consistent with the presence of hydroxy group and carbonyl functions [28].

**Figure 3:**
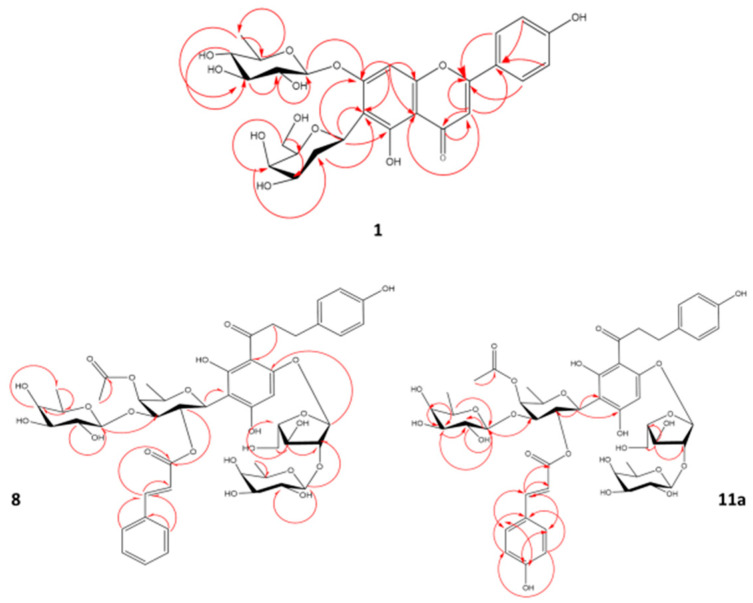
Key HMBC correlations of compound **1**, **8**, and **11a**.

The full assignment of ^1^H and ^13^C NMR data (Figures S2 and S3, Table 2) was adopted based on the analysis of the ^1^H-^1^H COSY, HSQC, and HMBC spectra (Figures S4–S6). The existence of a flavone unit could be easily assigned from the ^1^H NMR and ^13^C NMR spectra (Figures S2 and S3, Table 2) from key signals of 4 A_2_B_2_-type aromatic protons at δ 7.80 (2H, d, *J* = 8.8 Hz, H-2′/6′) and at δ 6.77 (2H, d, *J* = 8.8 Hz, H-3′/5′) for a *p*-disubstituted benzene ring, together with two aromatic singlets at δ 7.02 (H-8) and at δ 6.58 (H-3), referring to 6,7-disubstituted apigenin [29]. Moreover, ^13^C NMR (Figure S3) revealed 27 carbon resonances, which may be typical for di-glycosylated apigenin as follows: a carbon signal at δ 184.0 (C-4) assignable for a ketonic carbonyl, carbon resonances at δ 102.4 (C-3), δ 159.9 (C-5), δ 113.6 (C-6), δ 164.5 (C-7), and δ 96.4 (C-8) were similar to those reported for 6,7-disubstituted apigenin [29]. Excluding carbons of flavone unit, another 12 carbons were left assigned to two sugar moieties for deoxy-hexopyranosyl units. δ (Chemical shift) and *J* (coupling constant) values as well as the ^1^H-^1^H COSY spectrum (Figure S4) identified the first hexose moiety as 2-deoxy-β-D-galactose. The signals for an anomeric proton at δ 5.10 (dd, *J* = 12.1, 2.4 Hz, H-1″), two protons at δ 2.83 (q, *J* = 12.1 Hz, H_1_-2″) and at δ 1.59 (m, H_2_-2″), two protons at δ 4.02 (dd, *J* = 12.1, 2.1 Hz, H_1_-6″) and at δ 3.74 (dd, *J* = 12.1, 6.4 Hz, H_2_-6″), six carbons at δ 70.5 (C-1″), 32.3 (C-2″), 71.6 (C-3″), 78.7 (C-4″), 76.1 (C-5″), and δ 62.8 (C-6″) are consistent with those of a 2-deoxy-β-D-galactose [30] attached at the C-6 position in apigenin via a C-glycosidic linkage, confirmed via HMBC correlations (Figure 3). The second sugar was assigned as β-quinovopyranose attached to carbon 7 via an oxygen bridge, based on its anomeric proton and carbon at δ 4.92 (1H, d, *J* = 7.7 Hz, H-1‴) and δ 103.8 (C-1‴). Further, methyl protons at δ 1.26 (3H, d, *J* = 6.5 HZ, H-6‴), four oxymethine carbons at δ 75.0 (C-2‴), 77.1 (C-3‴), 71.8 (C-4‴), and 72.1 (C-5‴), and a methyl carbon at δ 17.9 (C-6‴) confirmed sugar constitution. This sugar unit was identified from large axial–axial coupling constants revealing the axial orientation of all the ring protons of this unit, in agreement with the literature [31]. Hence, compound **1** was identified as apigenin 6-*C*-(2-deoxy-β-D-galactopyranoside)-7-*O*-β-D-quinovopyranoside, a new compound first time to be isolated in planta.

Another novel dihydrochalcone reported for the first time is compound **8** (Figure 2), isolated as yellowish amorphous powder soluble in 100% MeOH, with an estimated molecular formula of C_49_H_60_O_23_, based on a deprotonated ion peak calculated at *m*/*z* 1015.34526 and detected at *m*/*z* 1015.3460 [M-H]^−^ (calculated C_49_H_59_O_23_^−^, error −0.7 ppm) in the HR-ESI-MS spectrum. The IR spectrum of compound 8 showed absorption bands ascribable to hydroxyl (3432 cm^−1^) and carbonyl moeity (1616 cm^−1^) [28]. The ^13^C/DEPT spectrum (Figure S40) revealed the presence of 49 carbon atoms with 48 directly attached protons (3 × CH_2_, 11 × C, 31 × CH, 4 × CH_3_). Analysis of the ^1^H-^1^H COSY, HSQC, and HMBC spectra led to their assignment as the dihydrochalcone skeleton [28] (Figures S41–S43, Table 3). Typical signals for the dihydrochalcone of four A_2_B_2_-type aromatic protons were detected at δ 6.91 (1H, br.s, H-2), δ 7.09 (1H, d, *J* = 8.4 Hz, H-6), 6.62 (1H, br.s, H-3), and δ 6.70 (1H, d, *J* = 8.4 Hz, H-5), with an aromatic proton singlet at δ 6.00 (H-5′) and four aliphatic methylene protons at δ 2.73 and δ 2.68 (H_2_-7) and δ 3.19 and δ 3.35 (H_2_-8) (Figure S38, Table 3). Further, ^13^C NMR (Figure S39, Table 3) revealed the signal at δ 205.1 (C-9) of a ketonic carbonyl and two aliphatic methylene carbons at δ 30.8 (C-7) and 49.1 (C-8), confirming the 3′-substituted phloretin structure. The presence of two trans-olefinic protons at δ 7.51 (1H, d, *J* = 16.0 Hz, H-7⁗) and 6.26 (1H, d, *J* = 16.0 Hz, H-8⁗), five aromatic protons at δ 7.51 (2H, d, *J* = 3.8 Hz, H-2⁗/6⁗) and 7.38 (3H, m, H-3⁗/4⁗/5⁗), a carboxyl carbon at δ 168 (C-9⁗⁗), two olefinic carbons at δ 146 (C-7⁗) and 119.3 (C-8⁗), six aromatic carbons, typical for a cinnamoyl unit [32] and a resonance for a single acetate methyl singlet at δ 2.02, and two carbons of an acetyl moiety at δ 173.6 and 20.5 [33] that suggested a phloretin-acetylated cinnamate. However, the HMBC experiment (Figure S43) could not clarify the connection of the acetyl group, most probably due to the need for using the low-temperature NMR technique [34]. Excluding carbons of dihydrochalcone, acetyl moiety, and trans cinnamoyl units, 23 carbons remained in the ^13^C NMR spectrum, assigned for four sugar moieties including three hexoses and a pentose. Sugars δ (chemical shift) and J (coupling constant) values as well as ^1^H-^1^H-COSY cross peaks and three hexose moieties were determined to be β-fucopyranosyls. The signals for an anomeric proton at δ 5.09 (1H, d, *J* = 9.9 Hz, H-1″), a methyl proton doublet at δ 1.31 (3H, d, *J* = 4.2 Hz, H_3_-6″), five oxymethine carbons at δ 74.1 (C-1″), 71.9 (C-2″), 84.5 (C-3″), 74.1 (C-4″), and 76.3 (C-5″), and a methyl carbon at δ 17.2 (C-6″) are consistent with those of a β-fucosyl moiety attached to C-3′ of the phloretin moiety via a *C*-glycosidic linkage [35]. The HMBC spectrum could not though confirm this linkage, as no correlation appeared between (H-1″) and C-2′, C-3′, or C-4′, which is likely attributed to the phenomenon of coexistence of two conformationally variant rotamers, due to restricted rotation around the single bond between C-9 and C-1 resulting from the steric hindrance of the cinnamoyl moiety [34]. Further, the change pattern of δ values at C-1″ (−1.7 ppm), C-2″ (+1.9), and C-3″ (−1.7), due to the esterification in comparison to the unesterified analog (carambolaside Ja), confirmed the connection of a cinnamoyl unit at C-2″ [34]. The downfield shift in C-3″ (Δδ + 1.6) and C-4″ (Δδ +0.5), relative to those in compound 7 (Figure S32), located the acetyl moiety at C-4″. A second hexose was assigned based on its anomeric signals at δ 4.36 (1H, d, *J* = 7.6 Hz, H-1‴) and δ 106 (C-1‴), a methyl proton doublet at δ 1.26 (3H, d, *J* = 6.4 Hz, H_3_-6‴), four oxymethine carbons at δ 72.3 (C-2‴), 74.7 (C-3‴), 73 (C-4‴), and 72 (C-5‴), and a methyl carbon at δ 16.8 (C-6‴), as β-fucopyranose connected via an oxygen linkage [35] between C-1‴ and C-3″, confirmed by the HMBC correlations from H-1‴ to C-3″ (Figure 3 and Figure S43). The third sugar signals were typical for a pentose from its anomeric proton at δ 5.71 (1H, s, H-A_1_), four oxymethine carbons at δ 106.6 (C-A_1_), 92.9 (C-A_2_), 76.3 (C-A_3_), and 84.1 (C-A_4_), and an oxymethylene carbon at δ 62 (C-A_5_) annotated as a α-arabinofuranosyl moiety [36]. Lastly, signals of a third β-fucopyranosyl moiety were assigned from its anomeric signal at δ 3.97 (1H, br.s, H-F_1_) and δ 105.6 (C-F_1_), methyl protons at δ 1.29 (1H, s, H_1_-F_6_) and δ 0.75 (2H, s, H_2_-F_6_), four oxymethine carbons at δ 72.9 (C-F_2_), 72.2 (C-F_3_), 75 (F_4_), and 71.9 (F_5_), and a methyl carbon at δ 16.9 (F_6_).

The HMBC experiment could not clarify the connection of the acetyl group, α-arabinofuranosyl moiety, or the last *β*-fucopyranosyl moiety, which warranted using the low-temperature NMR technique [34]. Altogether, compound **8** was identified as phloretin 3′-*C*-(2-*O-(E)-*cinnamoyl-3-*O*-β-D-fucopyranosyl-4-*O*-acetyl)-β-D-fucopyranosyl-6′-*O*-β-D fucopyranosyl-(1/2)-α-L arabinofuranoside.

Compound **11** (Figure 2), another novel dihydrochalcone, was isolated as a yellowish amorphous powder soluble in 100% MeOH. The molecular formula of compound **11** was established as C_49_H_60_O_24_, based on a deprotonated mol. ion peak calculated at *m*/*z* 1031.34018 and detected at *m*/*z* 1031.3389 [M-H]^−^ (calculated C_49_H_59_O_24_^−^, error +1.2 ppm) in its HR-ESI-MS spectrum (Figure S55). The IR spectrum of compound 11 revealed two major absorption bands at 3432 cm^−h^ and 1616 cm^−&^, consistent to hydroxyl and carbonyl moieties, respectively [28].

NMR spectral analysis confirmed that compound **11** existed in the form of a mixture of two diastereoisomers (**11a** and **11b**). ^1^H and ^13^C NMR data, in addition to the HSQC spectrum (Figures S56–S58), indicated a structure closely related to that of compound **8**, with an extra hydroxyl group characteristic for a p-coumaroyl moiety, instead of the cinnamoyl moiety in compound **8** existing in two diastereomers, i.e., (*E)* and (*Z*) isomers. Signals for *(E)*-isomer were assigned for the two olefinics at δ 7.45 (d, *J* = 14.3 Hz, H-7⁗) and at 6.05 (d, *J* = 14.3 Hz, H-8⁗), characteristic for an *(E)-p*-coumaroyl moiety in addition to four *p*-coupled aromatic protons at δ 7.36 (2H, d, *J* = 8.6 Hz, H-2⁗/6⁗) and 6.67 (2H, d, *J* = 8.5 Hz, H-3⁗/5⁗). Moreover, ^13^C NMR (Figure S57) exhibited a carboxyl carbon at δ 168.9 (C-9⁗), two olefinic carbons at δ 145.2 (C-7⁗) and 113.2 (C-8⁗), and six aromatic carbons, typical for a *(E*)-coumaroyl unit [37]. In contrast, (*Z*)-*p*-coumaroyl moiety exhibited signals of four para-coupled aromatic protons at δ 7.19 and 7.4 (2H, s, H-2⁗/6⁗), unresolved peaks corresponding to H-3⁗/5⁗, and two olefinic protons at δ 6.69 (d, *J* = 11.2 Hz, H-7⁗) and 5.60 (d, *J* = 11.6 Hz, H-8⁗), coupled with a characteristic constant of *J* = 11.2 Hz. Then, ^13^C-NMR exhibited a carboxyl carbon at δ 168.9 (C-9⁗), two olefinic carbons at δ 142.4 (C-7⁗) and 114.8 (C-8⁗), and six aromatic carbons typical for an (*Z*)*-*coumaroyl unit [37]. A resonance for a single acetate methyl singlet at δ 2.04 and two carbons of an acetyl moiety at (δ 172.0 and 19.1) were detected [33], as in compound **8**. The downfield-shifted C-3″ (Δδ + 0.3) and C-4″ (Δδ − 2), relative to those in compound **10** (Figure S51, Table 4), located the acetyl moiety at C-4″. The HMBC experiment (Figure 3) could not confirm the connection of the acetyl group most probably due to the need for using low-temperature NMR technique, as in **8** [34]. Consequently, compound **11a** was established as phloretin 3′-*C*-(2-*O-(E)-*p-coumaroyl-3-*O*-β-D-fucosyl-4-*O*-acetyl)-β-D-fucosyl-6′-*O*-(2-*O*-β-D-fucosyl)-α-L-arabinofuranoside. Whereas **11b** was assigned as phloretin 3′-*C*-(2-*O*-*(Z)*-p-coumaroyl-3-*O*-β-D-fucosyl-4-*O*-acetyl)-β-D-fucosyl-6′-*O*-(2-*O*-β-D-fucosyl)-α-L-arabinofuranoside. These compounds are reported for the first time in nature. Other identified compounds reported in the literature included carambolaside M (**5**) [11] (Figures S20–S24), carambolaside Ia (**6**) [11] (Figures S25–S29), carambolaside J (**7**) [11] (Figures S30–S36), carambolaside I (**9**) [34] (Figures S44–S48), carambolaside P and O (**10**) [11] (Figures S49–S54), carambolaside Q (**12**) [11] (Figures S61–S67), luteolin 6-C-α-L-rhamnopyranosyl-(1-2)-β-D-fucopyranoside (**2**) [38] (Figures S7–S11), apigenin 6-*C*-β-D-galactopyranoside (**3**) [39] (Figures S12–S14) and apigenin 6-C-α-L-rhamnopyranosyl-(1-2)-β-L-fucopyranoside (**4**) [38] (Figures S15–S19), by comparison of their spectroscopic data to those in previous references, but isolated for the first time from starfruit leaves.

### 2.3. Structure-Activity Relationship Assessment of Isolated Compounds as α-Glucosidase Inhibitors

To further confirm potential efficacy of CLL compounds, isolated compounds were tested for their in vitro α-glucosidase inhibitory activity, to assess their efficacy. Considering the limitation of yield, in vivo assay was not possible to be performed. The efficacy of the isolated compounds was measured and discussed in relationship to the flavonoid structures, as discussed in the next subsections for each class separately, to identify the most crucial motifs within each for activity.

#### 2.3.1. Structure-Activity Relationship Assessment of Flavones as α-Glucosidase Inhibitors

**Figure 4:**
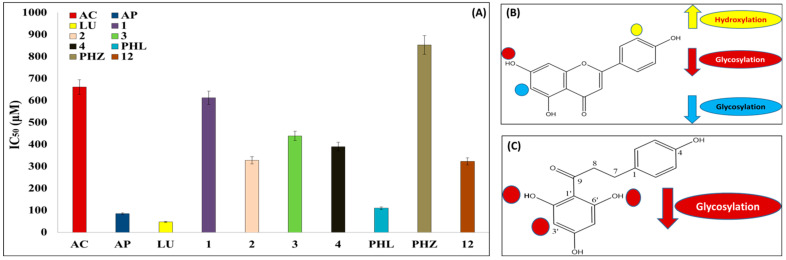
(**A**) IC_50_ (µM) of tested compounds against α-glucosidase enzymes in vitro. (**B**) The potential sites of flavone *C-*glycosides affecting α-glucosidase inhibitory potential. (**C**) The potential sites of dihydrochalcone *C*-glycosides affecting the α-glucosidase inhibitory potential. The up arrows represent increased inhibition, whereas the down arrows represent decreased inhibition activity. Results are expressed as mean ± SE (*n* = 3).

Tested flavone compounds **1**, **2**, **3**, and **4**, along with flavone standard aglycones, i.e., apigenin and luteolin, exhibited strong α-glucosidase inhibition, where IC_50_ values were determined at 613, 328, 439, and 390 μM, respectively, exceeding that of acarbose determined at 662 μM, a commercial α-glucosidase inhibitor anti-diabetic drug. The order of activity of the isolated flavone glycosides was as such, with IC_50_ values at 327.9, 390.4, 439.2, and 612.9 µM for compounds, viz., **2**, **4**, **3**, and **1**, which are much higher than those of acarbose. However, flavone glycosides were less potent than their corresponding aglycones, i.e., apigenin and luteolin, with IC_50_ values at 85.6 and 48.2 µM, respectively (Figure 4A,B, Table 5).

Among glycosides, compound **2**, identified as luteolin 6-C-α-L-rhamnopyranosyl-(1-2)-β-D-fucopyranoside, showed the highest inhibitory activity among all isolated flavone glycosides in line with its aglycone, suggestive for the improved efficacy of C-glycosyl flavone against α-glucosidase enzyme, which is in agreement with reports that sugar moiety attached at C-6 position improved efficacy against pancreatic lipase inhibitory activity [40], extended herein to include the α-glucosidase inhibition effect (Figure 4B).

In contrast, compound **1**, identified as apigenin 6-C-(2-deoxy-β-D-galactopyranoside)-7-O-β-D-quinovopyranoside, showed the weakest inhibitory activity among all isolated flavone glycosides, with IC_50_ 612.9 µM, likely attributed to the glycosylation of hydroxy group at the C-7 position [41] (Figure 4B) and absent in compounds **2**, **3**, and **4**. Compounds **3** and **4** exhibited strong inhibition with an IC_50_ value of 439.2 µM and 390.4 µM, respectively, in line with previously published data on the efficacy of apigenin 6-*C*-(2″-O-α-rhamnopyranosyl)-β-fucopyranoside in lowering the glucose level in hyperglycemic rats [40]. Standard apigenin and luteolin showed the strongest inhibitory activity, with IC_50_ values at 85.6 and 48.2 µM, respectively, compared to that of acarbose (661.6 µM), with luteolin showing the stronger inhibitory activity compared to that of acarbose, which is in accordance with the previously reported α-glucosidase inhibitory activity [42]. In line with our findings, hydroxylation at C-3′ of the B-ring of apigenin, in particular, was reported to enhance the α-glucosidase inhibition activity [41], whereas glycosylation affected it negatively compared to the aglycones [43].

#### 2.3.2. Structure-Activity Relationship of Dihydrochalcones and Their Glycosides as α-Glucosidase Inhibitors

The isolated dihydrochalcone glycosides, viz., compounds **5**, **6**, **7**, **8**, **9**, **10**, **11**, and **12**, were assessed for their α-glucosidase inhibitory activity in comparison to acarbose, together with two standard dihydrochalcones, phloretin and its glucoside phloretin-2′-glucose, commonly named phloridzin. All dihydrochalcone glycosides, viz., **5**, **6**, **7**, **8**, **9**, **10**, and **11**, were found inactive except for compound **12**, which showed relatively strong activity with IC_50_ at 323.6 µM (Figure 4A, Table 5). The reported weak α-glucosidase inhibitory activity of diglycosylated chalcones [41] clarified the inactivity of all isolated compounds and suggested that with regards to α-glucosidase inhibition, glycosylated forms of flavones are more active than dihydrochalcones. Further study is warranted, to carefully assess the α-glucosidase enzyme kinetic analysis of the dihydrochalcone carambolaside Q.

Regarding standard dihydrochalcones, phloretin was reported as a strong α-glucosidase inhibitor [44] and as a glucose transporter inhibitor [45], with a measured IC_50_ value at 110.4 µM (Figure 4A). Further, phloridzin revealed moderate inhibitory activity, with an IC_50_ of 853.1 µM (Figure 4A), in accordance with the reported dose-dependent α-glucosidase inhibition [46] and confirming that the inhibitory activity of monoglycosyl chalcones is lower than its aglycones [41] (Figure 4C). These results suggests that α-glucosidase inhibitory activity of *A. carambola* L. extract is mainly mediated by flavone glycosides composition, with a smaller contribution coming from dihydrochalcone glycosides being less active, except for compound **12**.

## 3. Materials and Methods

### 3.1. Plant Material

*A. carambola*, fresh leaf was collected from Groppy Arboretum, Giza, Egypt, in May 2021. The soil is of clay type with high humidity up to 90%. The tree grows in shade and is irrigated every 15 days. Plant material was authenticated by plant taxonomist Dr. Mohamed Gibali, Senior Botanist, Orman Botanic Garden (Giza, Egypt), and Mrs. Therese Labib, Consultant of Plant Taxonomy at the Ministry of Agriculture and Orman Botanic Garden, Giza, Egypt. A voucher specimen number (4754) was deposited in the (CAIM) Herbarium of Flora and Phytotaxonomy Researches Department, Horticultural Research Institute, Agricultural Research Center, Egypt.

Shade-dried powdered sample of *A. carambola* leaf (2 kg) was repeatedly extracted with 70% MeOH of analytical grade (Sigma Aldrich, St. Louis, MO, USA) in a water bath at 40 °C (3 × 5 L, each 48 h) until exhaustion and then filtered off. The filtrate was concentrated under reduced pressure to dryness at 55 °C to yield 500 g (25%) crude extract of *A. carambola* leaves. The obtained extract was kept at 4 °C for further phytochemical and biological assessments.

### 3.2. Chemicals

Biodiagnostic kits were purchased from Biodiagnostic Co. (Dokki, Giza, Egypt) for measurement of AST, ALT, urea, uric acid, creatinine, total cholesterol, HDL, LDL, MDA, leptin, insulin, glucose, α-amylase, BChe, and CAT levels. The enzyme α-glucosidase was purchased from Oriental Yeast Co. (Tokyo, Japan), while HEPES for making buffer solution was purchased from EMD Millipore Corp (Billerica, MA USA). Phenolic standards, i.e., luteolin, apigenin, phloretin, and phloridzin, and 5-fluorouracil as reference cytotoxic drug were purchased from Wako Pure Chemical Industries (Osaka, Japan). Orly as a reference anti-obese drug for in vivo experiment was obtained from Eva Pharma, Egypt. Acarbose as a reference antidiabetic for in vitro experiments was purchased from Wako Pure Chemical Industries (Tokyo, Japan).

### 3.3. Chromatographic and Spectroscopic Techniques

Polyamide 6S, Silica Gel 60 (60–120 mesh), and Sephadex LH-20 (Riedel-de Haën AG, Seelze, Germany) were used for column chromatography (CC). Medium-pressure liquid chromatography (MPLC) was performed using Reveleris Prep System set (Buchi, Flawel, Switzerland) with a UV-ELSD detector, a C-18 flash column (FP ID C18, 35–45 μm, 40 g). Celite No. 545 from Wako (Japan) was used for loading sample. Analytical pre-coated Silica Gel 60 F245 plates (NP-TLC), preparative reversed-phase silica gel 60 RP-18 F254S (RP_18_-PTLC) thin layer chromatography plates (Merck, Germany), and preparative normal phase silica Gel 70 FM (NP-PTLC) thin layer chromatography plates (Wako, Japan) were used for the final purification of compounds. Thin layer chromatography (TLC) plates were visualized under UV light at (254 and 365 nm) and sprayed with 10% MeOH-H_2_SO_4_ reagent, followed by heating for 2–3 min. Methanol used for extraction in CC was of analytical grade. Methanol and formic acid for MS and HPLC analyses were of HPLC grade. HPLC analysis was employed using an Agilent 1220 Infinity LC system, equipped with ELSD detector, a binary solvent delivery system, and an autosampler and connected to YMC column (5 μM, 4.6 × 150 mm, Japan). Aqueous formic acid (0.1%) and acetonitrile were used as mobile phases A and B, respectively, with the total flow rate at 1.0 mL/min for 35 min.

Detection of UV absorption of isolated compounds was done using a Shimadzu ultraviolet–visible (UV–Vis) 1601 recording spectrophotometer (P/N 206-67001, Kyoto, Japan) over the range of 190–500 nm was used for all measurements. Path length of cuvettes used was 1 cm. Manipulation of spectra was performed using UVProbe 2.42 software.

Optical rotation was measured on a Jasco DIP-370 polarimeter.

The NMR 1D and 2D spectra were recorded in CD_3_OD, using TMS as internal standard, and chemical shift values were recorded in δ ppm on a Bruker DRX 600 NMR spectrometer. Sample was completely dried to remove any residual solvent, resuspended in 600 µL deuterated methanol (CD_3_OD), and centrifuged prior to NMR analysis.

The HR-ESI-MS was acquired on an Agilent 6545 Q-TOF LC–MS system with dual electrospray ionization (ESI) (Santa Clara, CA, USA) in negative ionization mode, as it is more sensitive for the detection of phenolics, due to their acidic nature making it easier for them to lose protons. IR was recorded on an FTIR-6700 (JASCO, Tokyo, Japan). The sample was ground with KBr in a ratio of (1:10); the mixture is then pressed in disc form and placed into the sample hold, and the IR spectrum was run.

### 3.4. In Vivo Assessment of CLL Extract in an HFD Rat Anti-Obesity Activity

#### 3.4.1. Experimental Animals

Male albino rats of Sprague Dawley strain weighing 100–134 g (115.7 ± 7.6 g as mean ± SD) obtained from Animal House of National Research Centre, Cairo, Egypt, were kept on standard chow diet (8% fat) or high-fat diet (HFD) (30% saturated fat), provided with water ad libitum. Animals were kept individually in stainless steel metabolic cages at 25 °C; water and food were given ad libitum. All experiments were carried out according to the research protocols established by Research Ethics Committee in Faculty of Pharmacy, Cairo University, and by Medical Research Ethics Committee (MREC) in NRC, which follow the recommendations of the National Institutes of Health Guide for the Care and Use of Laboratory Animals Ethical Approval Certificate No. MP (1959).

#### 3.4.2. In Vivo Assay Experimental Design

Twenty-four rats were randomized into two groups and received either standard chow diet (8% fat, *n* = 6), as a normal control, or an HFD (30% saturated fat, *n* = 18) to induce obesity for 8 weeks [47]. Body weight and food intake were recorded every week. After induction of obesity, rats were divided into three subgroups and still fed on HFD. For subgroup 1, rats were fed on HFD and given the vehicle as obese control. For subgroup 2, rats were fed on HFD and given oral dose of Orly (10 mg/kg RBW/day) for 4 weeks as anti-obesity drug group. Rats in subgroup 3 were fed on HFD and given oral administration of crude methanol extract of *A. carambola* leaf (CLL), prepared as described in Section 3.1 (300 mg/kg RBW/day) for four weeks. Normal control rats were continued to be fed on standard chow diet for four weeks. Body weight and food intake were recorded every week. At the end of the experiment, blood samples were collected for determination of plasma total cholesterol (T-Ch) [48], high-density lipoprotein cholesterol (HDL-Ch) [49], low-density lipoprotein cholesterol (LDL-Ch) [50], and triglycerides (TG) [51]. T-Ch/HDL-Ch ratio was calculated as an indicator of cardiovascular risk. Plasma butyrylcholinesterase (BChE) [52], plasma α-amylase activity [53] was assessed. Plasma malondialdehyde (MDA) [54] and plasma catalase activity (CAT) [55] were estimated as indicators of lipid peroxidation and oxidative stress, respectively. For assessment of liver functions, the activity of plasma transaminases aspartate transaminase (AST) and alanine transaminase (ALT) were estimated, according to the method of [56]. Plasma level of creatinine [57], urea [58], and uric acid [59] were determined to assess changes in kidney functions. Plasma insulin [60] and blood glucose levels [61] were determined. Insulin resistance was calculated based on homeostasis model assessment of insulin resistance (HOMA-IR), according to [62]: (fasting plasma glucose (FPG) (mmoL/L) × fasting plasma insulin (FPI) (μU/mL))/22.5.

The animal experiment has been carried out according to Ethics Committee, National Research Centre, Cairo, Egypt, following the recommendations of the National Institutes of Health Guide for Care and Use of Laboratory Animals (Publication No. 85-23, revised 1985).

#### 3.4.3. Statistical Analysis

Statistical analyses were done using SPSS version 22. The results were expressed as mean ± standard error (SE) and analyzed statistically using one-way analysis of variance (ANOVA) followed by Duncan test. The statistical significance of difference was taken as *p* ≤ 0.05.

### 3.5. Isolation and Structural Elucidation

An amount of 150 g from aqueous methanol leaf extract (CLL; see Section 3.1) was fractionated using a polyamide column (Figure S69). Elution started with distilled H_2_O followed by H_2_O/MeOH, with gradual increase until reaching pure MeOH. The obtained fractions from the column (500 mL each) were examined using PC and TLC and observed under UV light. Similar fractions were pooled together, according to their TLC and PC profiles, to furnish 9 major fractions (A~I). Fraction B was the selected fraction for further purification based on TLC and PC detection. Fraction B (100% H_2_O, 40 g) was subjected to column chromatography (CC) on Sephadex LH-20 with aqueous MeOH for elution (30–100%). Similar fractions were pooled together, according to their TLC profiles, to furnish 7 fractions, B-1~B-7.

Fraction B-4 (30% MeOH, 700 mg) was subjected to reversed-phase flash column chromatography using a Buchi MPLC eluted with a gradient solvent mixture of MeOH/H_2_O (*v*/*v*, 4:6→ 5:5→ 6:4→ 7:3→8:2→9:1, each 400 mL, and flushed with 600 mL 100% MeOH). Similar fractions were pooled together, according to their TLC profiles, to afford 15 fractions, B-4-1~B-4-15. Fraction B-4-4 (*v*/*v*, 4:6 MeOH/H_2_O, 42 mg) was repeatedly purified with an n-PTLC to yield compound 5 (4 mg). Further, fraction B-4-11 (*v*/*v*, 5:5 MeOH/H_2_O, 150 mg) was repeatedly purified with an n-PTLC to yield compound 6 (3 mg). In addition, fraction B-4-13 (*v*/*v*, 5:5 MeOH/H_2_O, 25 mg) was repeatedly purified with an n-PTLC to yield compounds 7 and 8 (7 and 6 mg, respectively) (Figure S69).

Fraction B-6 (50% MeOH, 5.5 gm) was subjected to chromatographic separation using Sephadex LH-20 column eluted with *n*-butanol/H_2_O (1:1), to afford fractions B-6-1~B-6-9. Fraction B-6-6 (700 mg) was subjected to reversed-phase flash column chromatography using a Buchi MPLC and eluted with a gradient solvent mixture of MeOH/H_2_O (*v*/*v*, 20:80–100:0, each 400 mL) MPLC to afford 17 subfractions B-6-6-1~B-6-6-17. Subfraction B-6-6-3 (*v*/*v*, 4:6 MeOH/H_2_O, 16 mg) was repeatedly purified with an n-PTLC to yield compound 1 (7 mg). Further, subfraction B-6-6-12 (*v*/*v*, 6:4 MeOH/H_2_O, 40 mg) was repeatedly purified with an n-PTLC to afford compounds 9, 10 and 11 (6, 8.5 and 10 mg, respectively) (Figure S69).

Similarly, Fraction B-6-7 (2.2 g) was fractionated on a Sephadex LH-20 column, using *n*-butanol/H_2_O (1:1) to afford fractions B-6-7-1~B-6-7-3. Thereafter, subfraction B-6-7-3 (76 mg) was separated by reversed-phase flash column chromatography using a Buchi MPLC and eluted with a gradient solvent mixture of MeOH/H_2_O (*v*/*v*, 40:60–100:0, each 400 mL and flushed with 1000 mL 100% MeOH) to yield compound 2 (*v*/*v*, 5:5 MeOH/H_2_O, 7.4 mg) (Figure S69).

Fraction B-7 (50% MeOH, 1.14 g) was subjected to Sephadex eluted with butanol/H_2_O (1:1; *v*/*v*) to furnish fractions B-7-1~B-7-7. Moreover, the fractionation of fraction B-7-5 (146 mg) on reversed-phase flash column chromatography using a Buchi MPLC and eluted with a gradient solvent mixture of MeOH/H_2_O (*v*/*v*, 40:60 to 100:0, each 400 mL), which resulted in pure compounds 3, 4, and 12 (8, 4, and 5 mg, respectively).

Compound **1**: Yellowish amorphous powder (MeOH); [α]D^25^ −2.72 (c 0. 0044, MeOH); UV (MeOH) λ_max_ nm (log ε) 225 (2.39), 271 (2.43) and 333 (2.49) (Figure S70); IR (FTIR): ν = 3431, 2360, 1623 cm^1^ (Figure S71); R_t_ from HPLC 14.76 min; HR-ESI-MS detected at *m*/*z* 561.1614 [M-H]^−^ (calculated at *m*/*z* 561.16137, C_27_H_29_O_13_^−^, error −0.1 ppm); ^1^H (600 MHz) and ^13^C (150 MHz) NMR data in CD_3_OD, see Table 2.

Compound **2**: Yellowish amorphous powder (MeOH); [α]D^25^ −2.2 (c 0. 005, MeOH); UV (MeOH) λ_max_ nm (log ε) 226 (2.23), 272 (2.25) and 329 (2.37) (Figure S70); Rt from HPLC 14.59 min; HRESIMS *m/z* 577.1600 [M-H]^−^ (calculated C_27_H_29_O_14_^−^, error 6.53 ppm); ^1^H (600 MHz) and ^13^C (150 MHz) NMR data in CD_3_OD, see Table 2.

Compound **3**: Yellowish amorphous powder (MeOH); [α]D^25^ 15.0289 (c 0. 00519, MeOH); UV (MeOH) λ_max_ nm (log ε) 269 (1.33) and 335 (1.3) (Figure S70); R_t_ from HPLC 12.44 min; HRESIMS *m*/*z* 431.0986 [M-H]^−^ (calculated C_21_H_19_O_10_^−^, error 0.62 ppm); ^1^H (600 MHz) and ^13^C (150 MHz) NMR data in CD_3_OD, see Table 2.

Compound **4**: Yellowish amorphous powder (MeOH); [α]D^25^ 1.689 (c 0. 00296, MeOH); UV (MeOH) λ_max_ nm (log ε) 269 (2.73) and 336 (2.75) (Figure S70); R_t_ from HPLC 15.42 min; HRESIMS *m*/*z* 561.1661 [M-H]^−^ (calculated C_27_H_29_O_13_^−^, error 8.03 ppm); ^1^H (600 MHz) and ^13^C (150 MHz) NMR data in CD_3_OD, see Table 2.

Compound **5**: Yellowish amorphous powder (MeOH); [α]D^25^ +8.9 (c 0.003, MeOH); UV (MeOH) λ_max_ nm (log ε) 232 (1.92) and 286 (1.94) (Figure S70); R_t_ from HPLC 12.88 min; HRESIMS *m*/*z* 713.2272 [M-H]^−^ (calculated C_32_H_41_O_18_^−^, error 3.69 ppm); ^1^H (600 MHz) and ^13^C (150 MHz) NMR data in CD_3_OD, see Table 3.

Compound **6**: Yellowish amorphous powder (MeOH); [α]D^25^ −20.69 (c 0.0003, MeOH); UV (MeOH) λ_max_ nm (log ε) 228 (0.63) and 284 (0.54) (Figure S70); R_t_ from HPLC 14.37 min; HRESIMS *m*/*z* 697.2350 [M-H]^−^ (calculated C_32_ H_41_O_17_^−^, error 0.06 ppm); ^1^H (600 MHz) and ^13^C (150 MHz) NMR data in CD_3_OD, see Table 3.

Compound **7**: Yellowish amorphous powder (MeOH); [α]D^25^ −66.41 (c 0.003, MeOH); UV (MeOH) λ_max_ nm (log ε) 231 (2.53) and 284 (2.58) (Figure S70); R_t_ from HPLC 18.15 min; HRESIMS *m*/*z* 973.3340 [M-H]^−^ (calculated C_47_ H_57_O_22_^−^, error 1.1 ppm); ^1^H (600 MHz) and ^13^C (150 MHz) NMR data in CD_3_OD, see Table 3.

Compound **8**: Yellowish amorphous powder (MeOH); [α]D^25^ −40.39 (c 0.003, MeOH); UV (MeOH) λ_max_ nm (log ε) 230 (1.60) and 285 (1.65) (Figure S70); IR (FTIR): ν = 3433, 2921, 1616, 1516, 1449, 1382, 1222, 1173, 1073 cm^1^ (Figure S71); R_t_ from HPLC 18.14 min; HRESIMS detected at *m*/*z* 1015.3460 [M-H]^−^ (calculated at *m*/*z* 1015.34526, C_49_H_59_O_23_^−^, error −0.7 ppm); ^1^H (600 MHz) and ^13^C (150 MHz) NMR data in CD_3_OD, see Table 3.

Compound **9**: Yellowish amorphous powder (MeOH); [α]D^25^ −31.379 (c 0. 0015, MeOH); UV (MeOH) λ_max_ nm (log ε) 232 (1.99) and 290 (2.03) (Figure S70); R_t_ from HPLC 18.10 min; HRESIMS *m*/*z* 827.2768 [M-H]^−^ (calculated C_41_H_47_O_18_^−^, error 0.78 ppm); ^1^H (600 MHz) and ^13^C (150 MHz) NMR data in CD_3_OD, see Table 3.

Compound **10**: Yellowish amorphous powder (MeOH); [α]D^25^ −102.778 (c 0. 0014, MeOH); UV (MeOH) λ_max_ nm (log ε) 230 (1.94), 290 (1.99) and 310 (2.05) (Figure S70); R_t_ from HPLC 17.50 min; HRESIMS *m*/*z* 989.3289 [M-H]^−^ (calculated C_47_H_57_O_23_^−^, error 0.67 ppm); ^1^H (600 MHz) and ^13^C (150 MHz) NMR data in CD_3_OD, see Table 4.

Compound **11**: Yellowish amorphous powder (MeOH); [α]D^25^ −95.42 (c 0. 0024, MeOH); UV (MeOH) λ_max_ nm (log ε) 226 (1.27) and 286 (1.26) (Figure S70); IR (FTIR): ν = 3433, 2921, 1616, 1516, 1449, 1382, 1222, 1173, 1073 cm^1^ (Figure S71); R_t_ from HPLC 17.60 min; HRESIMS detected at *m*/*z* 1031.3389 [M-H]^−^ (calculated at *m*/*z* 1031.34018, C_49_H_59_O_24_^−^, error + 1.2 ppm); ^1^H (600 MHz) and ^13^C (150 MHz) NMR data in CD_3_OD, see Table 4.

Compound **12**: Yellowish amorphous powder (MeOH); [α]D^25^ −112.25 (c 0. 004, MeOH); λ_max_ nm (log ε) 226 (2.17), 290 (2.21) and 310 (2.19) (Figure S70); R_t_ from HPLC 16.94 min; HRESIMS *m*/*z* 843.2782 [M-H]^−^ (calculated C_41_H_47_ O_19_^−^, error 7.27 ppm); ^1^H (600 MHz) and ^13^C (150 MHz) NMR data in CD_3_OD, see Table 4.

### 3.6. In Vitro α-Glucosidase Inhibitory Assay

The assay of α-glucosidase inhibitory activity of compounds was adopted from [63]. Briefly, 100 μL of DMSO and 100 μL of α-glucosidase enzyme (5 U/mL in 0.15 M HEPES buffer) were added to 100 μL substrate (0.1 M sucrose solution dissolved into 0.15 M HEPES buffer). The mixture was vortexed for 5 sec and then incubated at 37 °C for 30 min to allow for enzymatic reaction. After incubation, the reaction was stopped by heating at 100 °C for 10 min in a block incubator. The formation of glucose was determined by means of glucose oxidase method, using a BF-5S Biosensor (Oji Scientific Instruments, Hyogo, Japan). Mathematically, α-glucosidase inhibitory activity of each sample was calculated according to this equation: (Average value of control (Ac) − average value of the sample (As))/Ac × 100.

The IC_50_ values were calculated from plots of log concentration of inhibitor concentration against the percentage inhibition curves, using Microsoft Excel 2016. The data were expressed as mean ± standard deviation (SD) of at least three independent experiments (*n* = 3).

## 4. Conclusions

**Figure 5:**
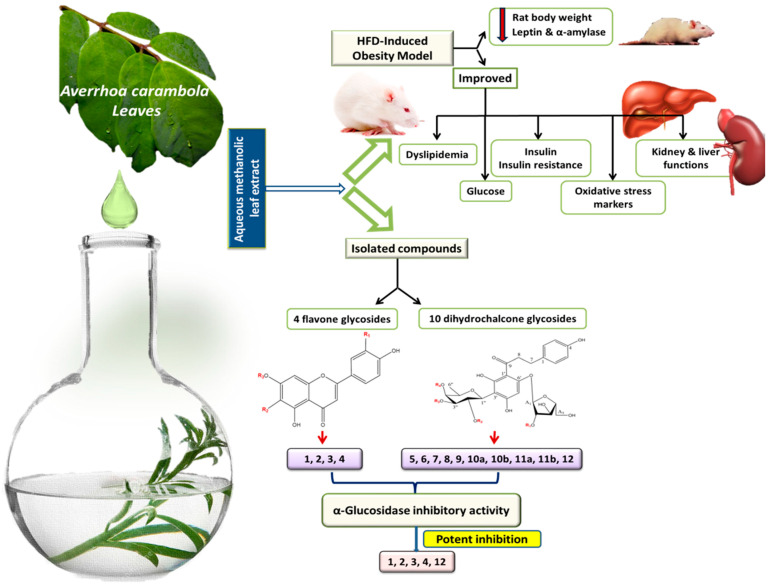
Collective scheme for extraction, isolation, and biological activities performed on *A. carambola* leaves.

The global quest for anti-obesity as well as anti-diabetic drugs is currently ongoing, as obesity and its complications continue to afflict the world’s population, warranting the discovery of new therapeutic regimens. A high-fat diet induced obesity model in rats was used for the assessment of anti-obese activity of *A. carambola* leaf extract, in relation to its phenolic composition. To the best of our knowledge, this study presents the first comprehensive attempt to reveal the in vivo anti-obese activity of *A. carambola* leaf extract, leading to the isolation of new bioactive components. Oral administration of *A. carambola* leaf extract enhanced all obesity complications, viz., dyslipidemia, hyperglycemia, insulin resistance, and oxidative stress, and exhibited significant anti-obesity activity in obese rats (Figure 5). Further, the effect of CLL was significantly better than Orly in almost all tested biochemical parameters, except for elevated uric acid level, although Orly revealed better reduction in body weight gain.

Multiple chromatographic approaches of the leaf extract led to the isolation of 14 compounds, including 4 flavone glycosides (**1**–**4**) and 10 dihydrochalcone glycosides (**5**–**12**) with two non-separable mixtures, including four newly described compounds, i.e., **1**, **8**, **11a**, and **11b** were reported for the first time in the literature. Further, in vitro α-glucosidase inhibitory activity assessment of isolated compounds revealed the strong potency of isolated flavone glycosides, viz., compounds **1**, **2**, **3**, and **4**, as α-glucosidase inhibitors, compared to dihydrochalcone glycosides, except for compound **12**. These results suggest for the role of flavone glycosides in alleviation of the major obesity comorbidity, i.e., diabetes via α-glucosidase inhibition, and has yet to be confirmed for other action mechanisms. An extended approach utilizing detailed studies on the molecular mechanisms of *A. carambola* leaf effect should now follow, together with subclinical and clinical trials on leaf crude extract, to be more conclusive, especially considering the known negative impact of its fruit on kidney functions. Moreover, assessment of the isolated phytoconstituents for their anti-obese activity using in vivo model or targeting other enzymes, i.e., lipases, etc., should now follow to correlate for the extract’s potential anti-obesity effect. This study poses *A. carambola* leaf as a new anti-obesity functional food and adds to its effects aside from its fruit’s more explored uses.

## Figures and Tables

**Table 1:** List of nutritional and biochemical parameters in obese, normal, and treated animal groups (*n* = 3).

No.	Measured Parameters	Tested Groups			
		Normal	Obese	Orly	CLL
1	Initial BW (g)	115.7 ^a^ ± 1.7	115.8 ^a^ ± 2.6	115.8 ^a^ ± 2.7	115.7 ^a^ ± 5.1
2	BW after induction of obesity (g)	217.7 ^a^ ± 7.8	248.7 ^b^ ± 8.3	248.8 ^b^ ± 7.5	249 ^b^ ± 3.8
3	Final BW (g)	246.5 ^b^ ± 10.1	291 ^d^ ± 4.9	233.2 ^a^ ± 11.2	258 ^c^ ± 8.7
4	Leptin (ng/mL)	12.8 ^a^ ± 0.3	24.1 ^d^ ± 0.4	21.09 ^c^ ± 0.1	18.0 ^b^ ± 0.2
5	Insulin (µg/L)	6.4 ^a^ ± 0.1	12.2 ^e^ ± 0.3	10.1 ^d^ ± 0.1	9.4 ^b^ ± 0.2
6	Glucose (mg/dL)	69.8 ^a^ ± 2.01	106.6 ^e^ ± 3.3	89.9 ^c^ ± 2.1	80.0 ^d^ ± 1.7
7	IR	1.1 ^a^± 0.1	3.2 ^e^± 0.1	2.2 ^d^ ± 0.1	1.9 ^c^ ± 0.1
8	BChE (U/L)	250.7 ^a^ ± 5.2	415.7 ^e^ ± 8.5	285.2 ^d^ ± 7.3	274.8 ^c^ ± 5.8
9	α-amylase (U/L)	9.2 ^a^ ± 0.4	15.33 ^e^ ± 0.3	13.7 ^d^ ± 0.3	12.4 ^c^ ± 0.2
10	MDA (nmol/mL)	5.6 ^a^ ± 0.2	15.1 ^e^ ± 0.6	12.9 ^d^ ± 0.6	10.4 ^c^ ± 0.4
11	CAT (U/L)	598.1 ^a^ ± 14.8	319.5 ^e^ ± 0.4	329.3 ^d^ ± 12.7	473.2 ^c^ ± 10.7
12	T-Ch (mg/dL)	70.4 ^a^ ± 2.1	136.6 ^e^ ± 5.4	97.9 ^d^ ± 3.9	89.7 ^c^ ± 3.8
13	TG (mg/dL)	67.7 ^a^ ± 1.9	113.3 ^e^ ± 2.4	99.5 ^d^ ± 4.2	87.6 ^c^ ± 2.4
14	HDL-Ch (mg/dL)	42.8 ^a^ ± 0.7	27.8 ^e^ ± 1.2	31.5 ^d^ ± 0.8	37 ^c^ ± 0.6
15	LDL-Ch (mg/dL)	19.5 ^a^± 0.7	85.5 ^d^ ± 3.5	69.2 ^c^ ± 1.8	49.8 ^c^ ± 1.4
16	T-Ch/HDL-Ch ratio	1.64 ^a^ ± 0.03	4.9 ^e^ ± 0.2	3.1 ^d^ ± 0.2	2.4 ^c^ ± 0.1
17	ALT (IU/L)	18.6 ^a^ ± 0.6	25.3 ^d^ ± 1.1	21.0 ^c^ ± 0.6	19.7 ^d^ ± 0.4
18	AST (IU/L)	41.9 ^a^ ± 0.7	49.7 ^b^ ± 1.1	43.8 ^c^ ± 0.6	43.2 ^d^ ± 0.7
19	Creatinine (mg/dL)	0.624 ^a^ ± 0.01	0.76 ^d^ ± 0.02	0.68 ^c^ ± 0.02	0.617 ^b^ ± 0.02
20	Urea (mg/dL)	24.6 ^a^ ± 1.1	33.2 ^d^ ± 0.9	26.8 ^c^ ± 0.6	25.0 ^b^ ± 0.6
21	Uric acid (mg/dL)	0.8 ^a^ ± 0.04	1.3 ^b^ ± 0.09	1.4 ^c^ ± 0.1	1.9 ^c^ ± 0.1

Results are expressed as mean ± S.E.M. Values with different superscript letters in the same raw are significantly different at *p* < 0.05 levels. ^b,c,d^ and ^e^ are significantly higher than ^a^.

**Table 2:** ^1^H (600 MHz) and ^13^C NMR (150 MHz) data of compounds **1**–**4** in CD_3_OD.

H/C	1		2		3		4	
	δ_H_ (*J* in Hz)	δ_C_	δ_H_ (*J* in Hz)	δ_C_	δ_H_ (*J* in Hz)	δ_C_	δ_H_ (*J* in Hz)	δ_C_
2		168.0		166.4		166.2		166.4
3	6.58, s	102.4	6.55, s	104.1	6.58, s	103.9	6.57, s	103.2
4		184.0		184.2		184.1		184.0
5		159.9		160.8		162.1		160.8
6		113.6		110.0		109.3		110.3
7		164.5		164.7		165.2		163.5
8	7.02, s	96.4	6.52, s	96.2	6.49, s	95.4	6.49, s	96.6
9		158.5		158.9		158.8		159.1
10		106.9		105.5		105.2		104.9
1″		118.8		123.7		123.2		122.9
2′	7.8, d (8.8)	129.8	7.38, br.s	114.2	7.82, d (8.4)	129.5	7.83, d (8.4)	129.4
3′	6.77, d (8.8)	119.3		147.1	6.92, d (8.5)	117.1	6.91, d (8.5)	117.3
4′		170.2		151.1		162.9		162.3
5′	6.77, d (8.8)	119.3	6.90, d (8.3)	116.9	6.92, d (8.5)	117.1	6.91, d (8.5)	117.3
6′	7.8, d (8.8)	129.8	7.39, br.s	120.4	7.82, d (8.4)	129.5	7.83, d (8.4)	129.4
1″	5.10, dd (12.1, 2.4)	70.5	4.92, d (9.8)	73.7	4.9, d (9.9)	75.4	4.91, d (9.5)	73.7
2″	2.83, q (12.1), 1.59, m	32.3	4.27, t-like (9.3)	75.8	4.16, t (8.9)	72.7	4.31, t (9.3)	76.1
3″	3.80, ddd (11.7, 4.9. 2.8)	71.6	3.75, dd (9.1, 6.2)	77.8	3.8, m	80.2	3.73, d (8.5)	77.9
4″	3.59, d (2.1)	78.7	3.70, br.s	74.1	3.7, dd (12.2, 5.4)	71.8	3.68, br.s	74.2
5″	3.60, d (2.1)	76.1	3.78, m	76.3	3.42, br.s	82.7	3.77, d (6.3)	76.1
6″	4.02, dd (12.1, 2.1), 3.74, dd (12.1, 6.4)	62.8	1.29, d (6.2)	17.2	3.48, m	62.9	1.28, d (5.9)	18.0
1‴	4.92, d (7.7)	103.8	5.19, br.s	102.4			5.17, s	102.4
2‴	3.64, dd (9.5, 7.8)	75	3.85, br.d	72.4			3.36, s	72.4
3‴	3.53, t (9.1)	77.1	3.47, d (8.2)	72.1			3.48, dd (3.2, 9.5)	72.2
4‴	3.40, t (9.4)	71.8	3.10, t-like (9.4)	73.4			3.10, t (9.5)	73.5
5‴	3.62, d (6.7)	72.1	2.56, m	69.9			2.63, m	69.9
6‴	1.26, d (3H, 6.5)	17.9	0.72, d (3H, 6)	18.0			0.73, d (5.9)	17.3

**Table 3:** ^1^H (600 MHz) and ^13^C NMR (150 MHz) data of compounds **5**–**9** in CD_3_OD isolated from CLL extract.

H/C	5		6		7		8		9	
	δ_H_ (*J* in Hz)	δ_C_	δ_H_ (*J* in Hz)	δ_C_	δ_H_ (*J* in Hz)	δ_C_	δ_H_ (*J* in Hz)	δ_C_	δ_H_ (*J* in Hz)	δ_C_
1		134.2		134.2		134.1		134.2		134.1
2	7.07, d (8.4)	130.5	7.11, d (8.4)	130.5	6.89, br.s	130.5	6.91, br.s	130.5	7.09, d (8.3)	130.5
3	6.67, d (8.4)	116.1	6.72, d (8.4)	116.3	6.61, br.s	116.4	6.62, br.s	116.3	6.70, d (8.4)	116.3
4		156.5		156.7		156.6		156.6		156.6
5	6.67, d (8.4)	116.1	6.72, d (8.4)	116.3	6.61, br.s	116.4	6.70, d (8.4)	116.3	6.70, d (8.4)	116.3
6	7.07, d (8.4)	130.5	7.11, d (8.4)	130.5	6.89, br.s	130.5	7.09, d (8.4)	130.5	7.09, d (8.3)	130.5
7	2.87, t (7.4)	31.4	2.91, d (5.7) 1.31, s	30.8	2.73/2.65, br.s	30.9	2.73/2.68, br.s	30.8	2.91, m/1.29, s	30.8
8	3.36, t (7.4)	46.6	3.41 unresolved	45.8	3.35/3.06	47.6	3.35/3.19, br.s	49.1	3.35/3.41	46.2
9		204.8		203.6		205.4		205.1		205.2
1′		106.4		106.5		106.0		106.0		106.8
2′		167.5		168		165.5		168		165.8
3′		107.1		106.5		106.0		106.0		106.8
4′		167.5		168		164.6		166.3		165.1
5′	6.05, s	98.3	6.03, s	99.5	6.12, s	97.4	6.00, S	98.7	6.13, S	97.6
6′		161.5		161.6		161.8		161.8		161.5
1″	4.77, d (9.9)	76.8	4.78, d (9.8)	76.1	5.11, d (9.5)	74.1	5.09, d (9.9)	74.1	5.07, d (9.7)	73.9
2″	4.36, br.s	71.3	4.43, t (9.4)	70.3	5.78, br.s	71.8	5.88, br.s	71.9	4.78, br.s	75.0
3″	3.63, dd (9.7, 3.3)	77.5	3.52, dd (9.4, 3.2)	77.7	3.96, br.s	82.9	3.97, br.s	84.5	3.53, dd (9.3, 3)	77.1
4″	3.95, d (3)	84.2	3.48, d (1.8)	74.4	3.95, d (2.5)	73.6	3.97, br.s	74.1	3.69, d (2.9)	73.7
5″	3.77, q (6.4)	76.1	3.74, q (6.7)	76	3.88, br.s	76.5	3.85, d (5.8)	76.3	3.73, q (6.5)	76.0
6″	1.33, d (3H, 6.4)	17.6	1.27, d (3H, 6.5)	17.2	1.33, d (3H, 6)	17.2	1.31, d (3H, 4.2)	17.2	1.32, d (6), 1.26, d (2H, 6.5)	17.2
1‴	4.58, d (7.7)	106.4			4.37, d (7.6)	105.7	4.36, d (7.6)	106.0		
2‴	3.34, m	76.2			3.48, dd (9.7, 7.7)	72.3	3.49, dd (9.7, 7.7)	72.3		
3‴	3.28, m	78.2			3.38, dd (9.7, 3.4)	74.8	3.38, dd (9.8, 3.4)	74.7		
4‴	3.35, m	71.3			3.55, d (3.4)	73.0	3.55, d (3.2)	73.0		
5‴	3.40, m	78.3			3.62, q (6.9)	72.0	3.62, q (6.5)	72.0		
6‴	3.85, dd (11.9, 2.1) 3.71, dd (11.8, 5.2)	62.7			1.26, d (3H, (6.5)	16.9	1.26, d (3H, 6.4)	16.8		
1⁗						136.0		136.0		136.4
2⁗					7.50, d (6.1)	129.3	7.51, d (3.8)	129.3	7.52, dd (7.4, 3.5)	129.2
3⁗					7.39, br.s	130.1	7.38, br.s	130.0	7.40, m	130.0
4⁗					7.39, br.s	131.4	7.38, br.s	131.4	7.40, m	131.5
5⁗					7.39, br.s	130.1	7.38, br.s	130.0	7.40, m	130.0
6⁗					7.50, d (6.1)	129.3	7.51, d (3.8)	129.3	7.60, dd (7.4, 3.5)	129.2
7⁗					7.51, d (16.0)	146.1	7.51, d (16.0)	146.0	7.69, d (16.0)	146.3
8⁗					6.27, d (16.0)	119.2	6.26, d (13.1)	119.3	6.53, d (16.0)	118.6
9⁗						167.7		168.0		167.7
A1	5.59, d (1.1)	108.1	5.78, d (1.2)	107.5	5.73, s	106.9	5.71, s	106.6	5.78, s	106.8
A2	4.04, dd (9.5, 6)	87.1	4.31, dd (4.6, 1.6)	92.6	4.19, br.s	92.9	4.19, br.s	92.9	4.29, m	92.5
A3	4.00, dd (6, 3.6)	78.2	4.17, dd (7.7, 4.9)	76.3	4.11, dd (7.9, 4.9)	76.3	4.10, dd (8, 5)	76.3	4.11, dd (7.9, 4.9)	76.0
A4	4.25, dd (3.6, 1.7)	83.5	4, m	84.5	3.99, br.s	84.4	3.97, br.s	84.1	4.00, br.s	84.2
A5	3.65, dd (12.1, 4.7) 3.73, dd (8.1, 3.1)	62.7	3.67, dd (12.5, 4.6) 3.8, dd (12.5, 2.8)	62.2	3.62, dd (13.3, 6.4), 3.77, dd (13.4, 6.3)	62.1	3.62, dd (13.4, 6.5), 3.78, br.s	62.0	3.66, dd (12.4, 4.8), 3.79, dd (12.4, 2.9)	62.2
F1			4.14, s	105.4	3.99, br.s	105.2	3.97, br.s	105.6	4.15, br.s	105.4
F2			3.48, m	72.9	3.41, br.s	72.9	3.41, br.s	72.9	3.47, dd (9.2, 3.4)	72.9
F3			3.37, dd (9.7, 3.4)	72	3.25, br.s	72.2	3.23, br.s	72.2	3.25, br.s	72.2
F4			3.37, s	75	3.43, br.s	75	3.42, br.s	75	3.36, d (3.4)	75
F5			3.26, q (6.5)	72.2	2.93, br.s	71.8	2.94, br.s	71.9	3.25, q (6.3)	72.0
F6			1.01, d (6.5)	16.7	1.29, 0.78, s (3H)	16.9	1.29, s (2H), 0.75, br.s	16.9	1.00, d (3H, 6.4)	16.9
CH_3_ CO-4″							2.02, S	20.5		
CO acetyl								173.6		

**Table 4:** ^1^H (600 MHz) and ^13^C NMR (150 MHz) data of compounds **10**–**12** in CD_3_OD.

H/C	10				11				12	
	10a (*Z*-isomer)		10b (*E*-isomer)		11a (*Z*-isomer)		11b (*E*-isomer)			
	δ_H_ (*J* in Hz)	δ_C_	δ_H_ (*J* in Hz)	δ_C_	δ_H_ (*J* in Hz)	δ_C_	δ_H_ (*J* in Hz)	δ_C_	δ_H_ (*J* in Hz)	δ_C_
1		134.2		133.8		134.7		134.4		134.2
2	6.93/7.00, br.s	130.5	7.09, d (8.2)/7.18, s	130.6	6.93/7.02, br.s	129.0	7.11, d (8.5)/7.23, d (8.2)	129.1	6.90, br.s	130.5
3	6.75, d (6.1)	117.6	6.70, d (8.5)	116.4	6.79, d (8.4)	115.9	6.72, d (8.5)	114.9	6.63, d (7.4)	116.4
4		156.6		156.5		155.1		155.1		156.6
5	6.75, d (6.1)	117.6	6.70, d (8.5)	116.4	6.79, d (8.4)	115.9	6.72, d (8.5)	114.9	6.63, d (7.4)	116.4
6	6.93/7.00, br.s	130.5	7.09, d (8.2)/7.18 s	130.6	6.93/7.02, br.s	129.0	7.11, d (8.5)/7.23, d (8.2)	129.1	6.90, br.s	130.5
7	2.77/2.70, br.s	30.8	2.77/2.89, br.s	30.8	2.78/2.68, br.s	29.3	2.78/2.91, br.s	29.3	2.65/2.75, br.s	30.4
8	3.35/3.07	46.6	3.35/3.16	46.6	3.37/unresolved	45.0	3.37/unresolved	45.0	3.09/3.36, br.s	47.5
9		204.9		204.9		204.1		204.1		206
1′		105.8		105.8		104.5		104.5		105.7
2′		165.8		165.8		167.1		167.1		165.5
3′		105.6		106.5		104.5		104.5		106
4′		165.8		165.8		167.1		167.1		164.9
5′	6.09, s	97.7	5.95, s	97.7	6.16, s	95.7	6.11, s	95.7	6.15, s	96.3
6′		161.8		161.8		160.3		160.3		161.7
1″	5.09, d (9.9)	74.1	5.02, d (9.8)	74.1	5.04, d (9.9)	72.6	5.12, d (9.5)	72.6	5.07, d (7.8)	74
2″	5.79, br.s	71.9	6.23, br.s	71.5	5.78, br.s	70	6.26, br.s	70	5.52, br.s	72.9
3″	3.91, br.d (9.7)	82.5	3.91, br.d (9.7)	82.5	3.94, br.s	82.8	3.94, br.s	82.8	3.85, br.s	74.9
4″	3.96, br.s	73.6	3.96, br.s	73.6	3.98, s	71.6	3.98, s	71.6	3.78, m	73.6
5″	3.82, m	76.3	3.86, br.s	76.3	3.85, br.d (6.7)	74.8	3.88, br.d (6.6)	74.8	3.82, m	76.9
6″	1.31, d (3H, 6.8)	17.2	1.29, br.s	17.2	1.31, d (3H, 6.8)	17.2	1.29, br.s	17.2	1.33, d (3H, 6.4)	17.2
1‴	4.36, d (7.6)	105.9	4.30, d (6.8)	105.9	4.38, d (7.6)	104.5	4.32, d (6.8)	104.5		
2‴	3.48, dd (9.7, 7.7)	72.2	3.46, br.d (7.2)	72.2	3.50, dd (9.7, 7.7)	70.8	3.46, br.d (7.2)	70.7		
3‴	3.39, dd (9.8, 3.4)	75.0	3.39, dd (9.8, 3.4)	74.9	3.41, dd (9.8, 3.4)	73.6	3.41, dd (9.8, 3.4)	73.5		
4‴	3.55, d (3.5)	73.0	3.56, d (4.0)	72.9	3.57, d (3.6)	71.4	3.58, d (4.1)	71.5		
5‴	3.62, q (6.9)	72.0	3.62, q (6.9)	72.0	3.63, q (6.6)	70.5	3.63, q (6.6)	70.4		
6‴	1.26, d (3H, 6.4)	16.9	1.26, d (3H, 6.4)	16.9	1.26, d (3H, 6.4)	15.4	1.26, d (3H, 6.4)	15.4		
1⁗		127.3		127.3		125.9		125.9		127.2
2⁗	7.33, d (8.4)	131.3	7.18/7.37	133.3	7.36, d (8.6)	129.8	7.19/7.4	132.7	7.37, d (8.5)	131.2
3⁗	6.65, d (8.4)	116.1	6.95, d (8.9)	116.3	6.67, d (8.5)	114.5			6.81, d (8.5)	117
4⁗		163.7		163.7		164.1		164.1		161.5
5⁗	6.65, d (8.4)	116.1	6.95, d (8.9)	116.3	6.67, d (8.5)	114.5			6.81, d (8.5)	117
6⁗	7.33, d (8.4)	131.3	7.18/7.37	133.3	7.36, d (8.6)	129.8	7.19/7.4	132.7	7.37, d (8.5)	131.2
7⁗	7.43, d (15.9)	146.8	6.66, d (11.2)	144.7	7.45, d (14.3)	145.2	6.69, d (11.2)	142.4	7.45, d (15.9)	146.4
8⁗	6.01, d (15.0)	114.4	5.59, d (12.4)	116.4	6.05, d (14.3)	113.2	5.60, d (11.6)	114.8	6.08, d (15.9)	115.3
9⁗		168.8		168.8		168.9		168.9		168.4
A1	5.71, s	105.9		105.9	5.74, s	104.5	5.92, s	104.5	5.73, br.s	107.1
A2	4.15, m	93.1	4.31, br.s	93.1	4.18, m	91.5	4.31, br.s	91.5	4.17, br.s	93
A3	4.10, dd (7.9, 4.9)	76.1	4.16, dd (7.7, 3.8)	76.3	4.13, dd (7.9, 4.9)	74.8	4.18, dd (6.7, 4.4)	74.8	4.11, dd (7.9, 4.8)	76.3
A4	3.94, m	84.1	3.94, m	84.1	3.94, m	84.3	3.94, m	84.3	3.95, ddd	84.5
A5	3.62, m, 3.77, br.s	62.0	3.62, m, 3.68, br.s	62.0	3.65, d (11.8, 5.3), 3.79, m	60.6	3.65, d (11.8, 5.3), 3.79, m	60.6	3.63, dd (12.1, 4.7), 3.83, br.d (12.3)	62
F1	3.97, d (5.6)	105.1	4.06, br.s	105.1	4.00, d (5.2)	104.5	4.08, br.s	104.5	3.96, d (7.6)	105.4
F2	3.38, dd (9.8, 6.4)	72.9	3.53, br.d (6.6)	72.9	3.4, dd (9.8, 6.4)	73.2	3.54, m	71.4	3.41, dd (9.6, 7.9)	72.2
F3	3.23, br.s	74.7	3.33, m	74.8	3.25, m	73.2	3.35, m	73.4	3.24, br.d (6.7)	74.90
F4	3.45, d (3.4)	72.3	3.42, d (3.2)	72.4	3.48, br.s	70.9	3.44, d (3.1)	70.8	3.40, d (3.3)	72.8
F5	2.89, br.s	71.5	3.19, m	71.5	2.91, br.s	70.4	3.21, m	70.5	2.9, br.s	71.8
F6	0.89, 0.76, s (3H)	16.9	0.94, 0.76 (3H)	16.9	0.89, 0.76, s (3H)	15.7	0.94, 0.76, s (3H)	15.7	0.79, br.s (3H)	16.9
CH_3_ CO-4″					2.04, s	19.1	2.04, s	19.1		
CO acetyl						172.0		172.0		

**Table 5:** IC_50_ of tested compounds using in vitro α-glucosidase inhibition assay. (-) indicates inactive compounds.

Class	Code	Compound Name	IC_50_ (μM)
**Standard**	**AC**	Acarbose	661.6 ± 0.01
**Flavone**	**Ap**	Apigenin	85.6 ± 0.01
	**LU**	Luteolin	48.2 ± 0.02
	**1**	Apigenin 6-*C*-(2-deoxy-β-D-galactopyranoside)-7-*O*-β-D-quinovopyranoside	612.9 ± 0.03
	**2**	Luteolin 6-*C*-α-L-rhamnopyranosyl-(1-2)-β-D-fucopyranoside	327.9 ± 0.05
	**3**	Apigenin 6-*C*-β-D-galactopyranoside	439.2 ± 0.01
	**4**	Apigenin 6-*C*-α-L-rhamnopyranosyl-(1-2)-β-L-fucopyranoside	390.4 ± 0.2
**Dihydrochalcone**	**PHL**	Phloretin	110.4 ± 0.06
	**PHZ**	Phloridzin	853.1 ± 0.02
	**5**	carambolaside M	-
	**6**	carambolaside Ia	-
	**7**	carambolaside J	-
	**8**	4″-*O*-acetyl-carambolaside J	-
	**9**	carambolaside I	-
	**10**	mix of carambolaside P and carambolaside O	-
	**11**	Mix of 4″-*O*-acetyl-carambolaside P and 4″-*O*-acetyl-carambolaside O	-
	**12**	carambolaside Q	323.6 ± 0.06

## Supplementary Materials

The following are available online at https://www.mdpi.com/article/10.3390/molecules27165159/s1. HRESIMS: ^1^H NMR: ^13^C NMR, ^1^H-^1^H COSY, HSQC, and HMBC spectra of compound **1** (Figures S1–S6); HRESIMS, ^1^H NMR, ^13^C NMR, HSQC, and HMBC spectra of compound **2** (Figures S7–S11); HRESIMS, ^1^H NMR, and ^13^C NMR spectra of compound **3** (Figures S12–S14); HRESIMS, ^1^H NMR, ^13^C NMR, HSQC, and HMBC spectra of compound **4** (Figures S15–S19); HRESIMS, ^1^H NMR, ^13^C NMR, HSQC, and HMBC spectra of compound **5** (Figures S20–S24); HRESIMS, ^1^H NMR, ^13^C NMR, HSQC, and HMBC spectra of compound **6** (Figures S25–S29); HRESIMS, ^1^H NMR, ^13^C NMR, DEPT-135, ^1^H-^1^H COSY, HSQC, and HMBC spectra of compound **7** (Figures S30–S36); HRESIMS, ^1^H NMR, ^13^C NMR, ^1^H-^1^H COSY, DEPT-135, HSQC, and HMBC spectra of compound **8** (Figures S37–S43); HRESIMS, ^1^H NMR, ^13^C NMR, HSQC, and HMBC spectra of compound **9** (Figures S44–S48); HRESIMS, ^1^H NMR, ^13^C NMR, HSQC, and HMBC spectra of compound **10** (Figures S49–S54); HRESIMS, ^1^H NMR, ^13^C NMR, HSQC, and HMBC spectra of compound **11** (Figures S55–S60); HRESIMS, ^1^H NMR, ^13^C NMR, HSQC, ^1^H-^1^H COSY, DEPT-135, and HMBC spectra of compound **12** (Figures S61–S67); Scheme of isolation (Figure S69); UV spectra of isolated compounds (Figure S70); IR spectra of new compounds **1**, **8**, **11** (Figure S71).
