# Supplementary material for: Anti-Obesity Evaluation of Averrhoa carambola L. Leaves and Assessment of Its Polyphenols as Potential α-Glucosidase Inhibitors
Source: Molecules. 2022 Aug 12;27(16):5159. doi: 10.3390/molecules27165159 (PMC9413271; doi:10.3390/molecules27165159)
Supplement: Supplementary file 1 [file molecules-27-05159-s001.zip › molecules-1858260-supplementary.pdf]

Supplementary data

Figure captions

Figure S1-S6. HRESIMS, ^{1}H NMR, ^{13}C NMR, ^{1}H–^{1}H COSY, HSQC, and HMBC spectra of compound **1**

Figure S7-S11. HRESIMS, ^{1}H NMR, ^{13}C NMR, HSQC, and HMBC spectra of compound **2**

Figure S12-S14. HRESIMS, ^{1}H NMR & ^{13}C NMR spectra of compound **3**

Figure S15-S19. HRESIMS, ^{1}H NMR, ^{13}C NMR, HSQC, and HMBC spectra of compound **4**

Figure S20-S24. HRESIMS, ^{1}H NMR, ^{13}C NMR, HSQC, and HMBC spectra of compound **5**

Figure S25-S29. HRESIMS, ^{1}H NMR, ^{13}C NMR, HSQC, and HMBC spectra of compound **6**

Figure S30-S36. HRESIMS, ^{1}H NMR, ^{13}C NMR, DEPT-135, ^{1}H–^{1}H COSY, HSQC, and HMBC spectra of compound **7**

Figure S37-S43. HRESIMS, ^{1}H NMR, ^{13}C NMR, ^{1}H–^{1}H COSY, **DEPT-135,** HSQC, and HMBC spectra of compound **8**

Figure S44-S48. HRESIMS, ^{1}H NMR, ^{13}C NMR, HSQC, and HMBC spectra of compound **9**

Figure S49-S54. HRESIMS, ^{1}H NMR, ^{13}C NMR, HSQC, and HMBC spectra of compound **10**

Figure S55-S60. HRESIMS, ^{1}H NMR, ^{13}C NMR, HSQC, and HMBC spectra of compound **11**

Figure S61-S67. HRESIMS, ^{1}H NMR, ^{13}C NMR, HSQC, ^{1}H–^{1}H COSY, DEPT-135 and HMBC spectra of compound **12**

Figure S68. HPLC purity check of isolated compounds.

Figure S69. Scheme of isolation

Figure S70. UV spectra of isolated compounds

Figure S71. IR spectra of new compounds 1, 8, 11

Compound 1

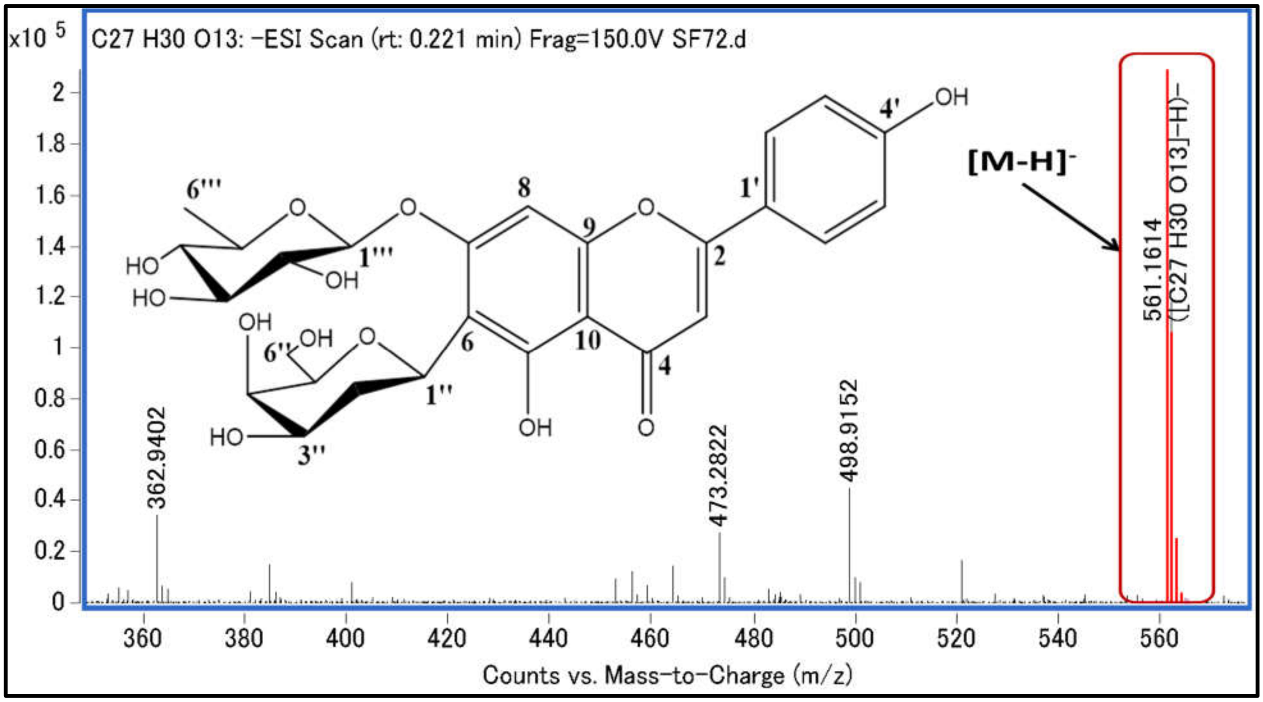

Figure S1: HRESIMS of compound 1

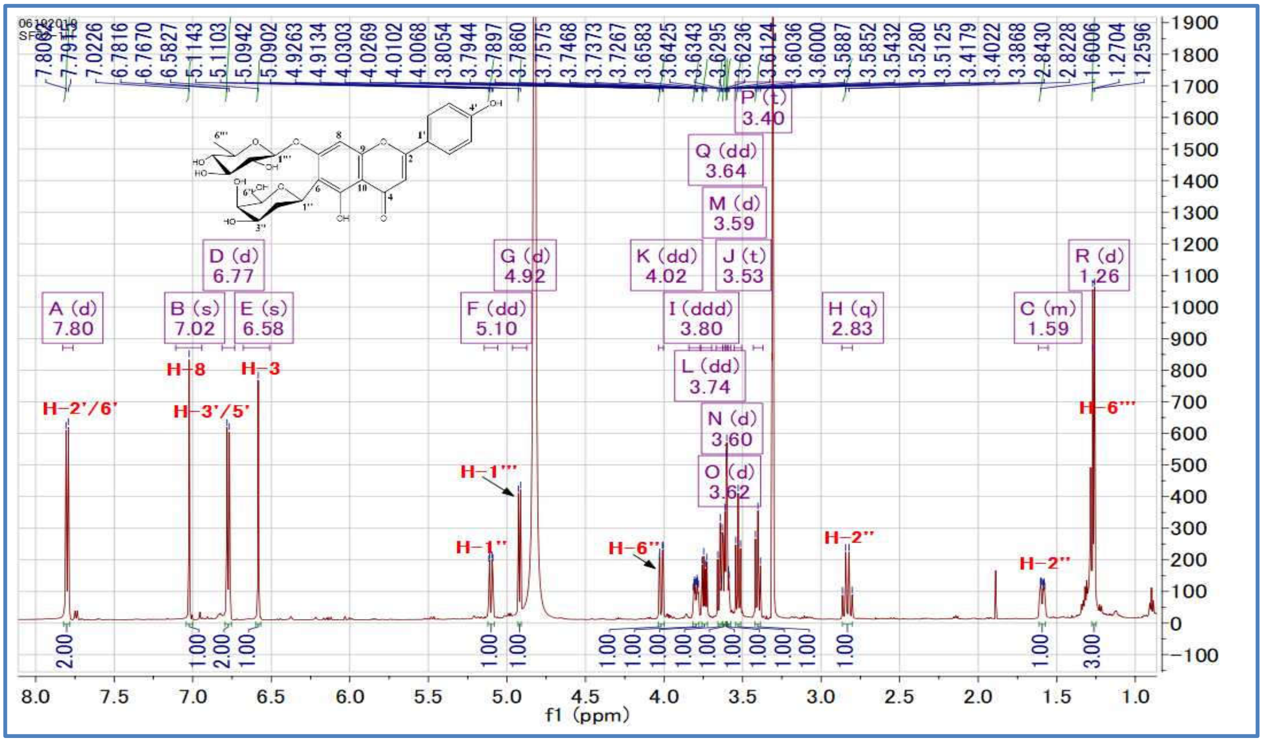

Figure S2: [1] H-NMR spectrum of compound 1 (CD_3OD).

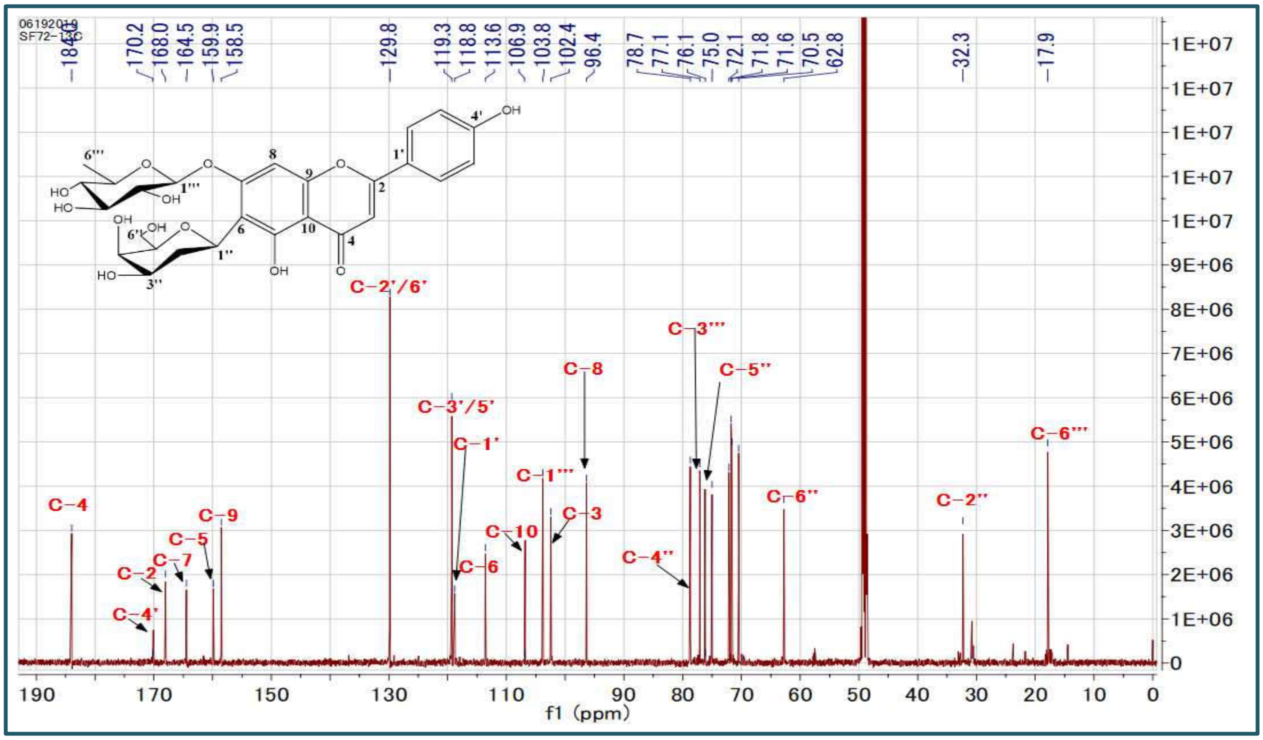

Figure S3: ^{13}C-NMR spectrum of compound 1 (CD_3OD).

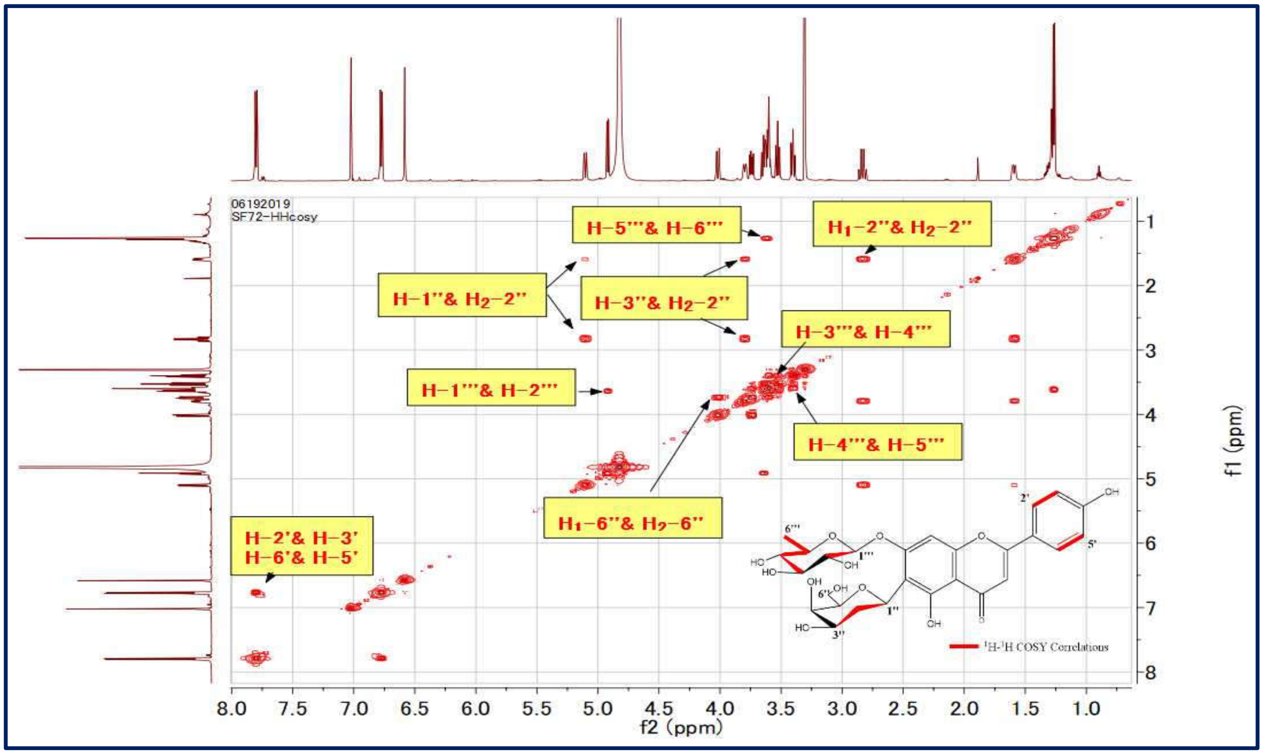

Figure S4: ^{1}H-^{1}H COSY spectrum of compound 1 (CD_3OD).

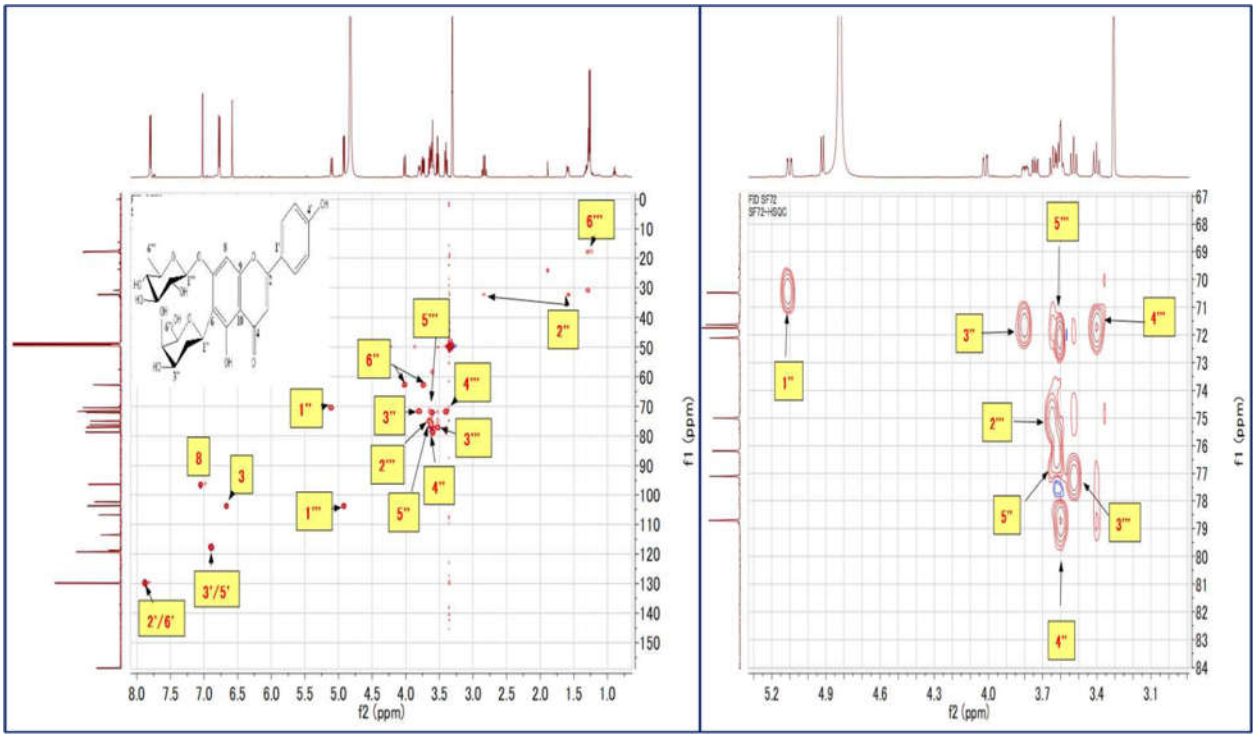

Figure S5: HSQC spectrum of compound 1 (CD_3OD).

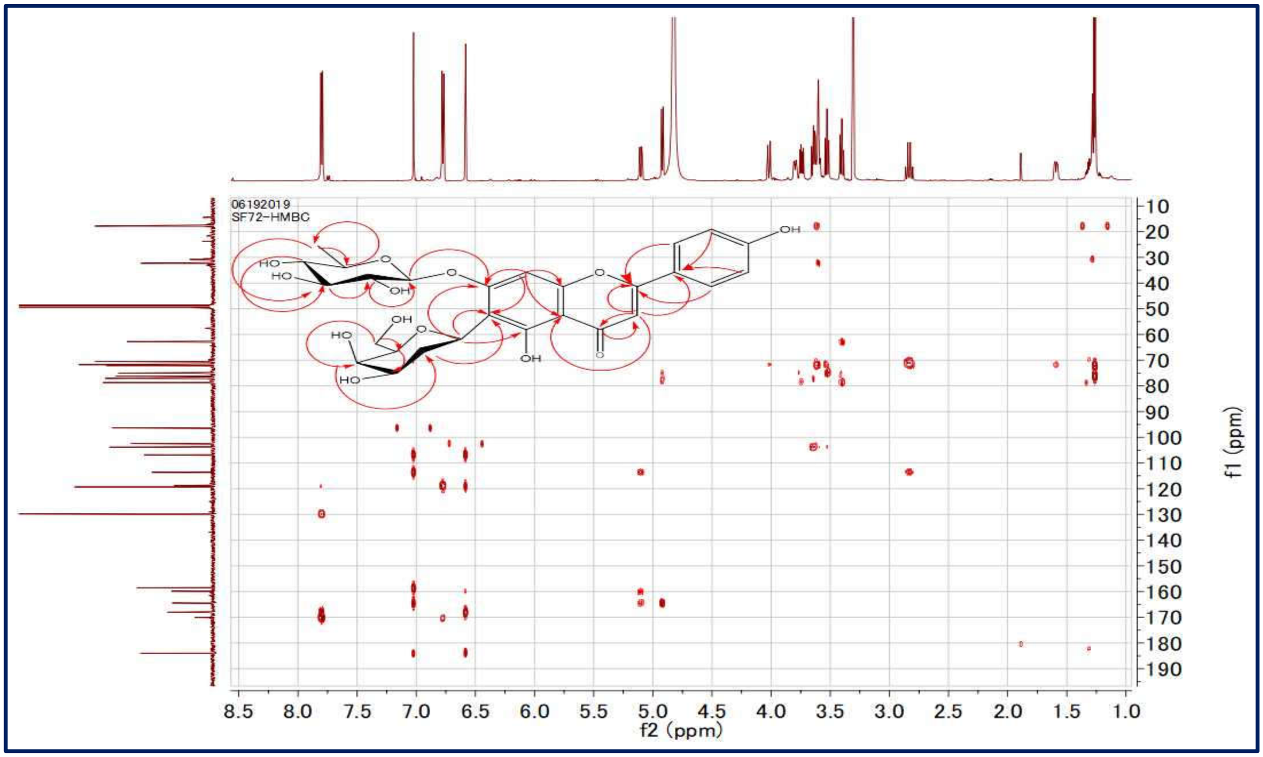

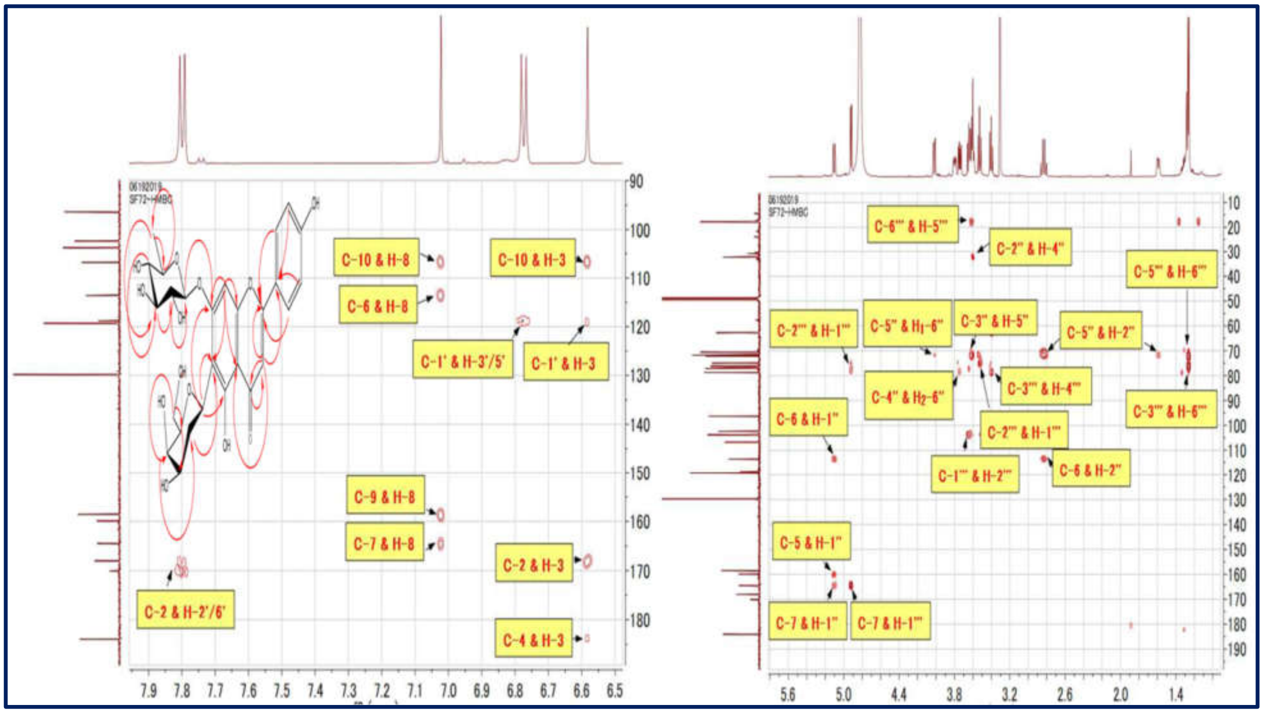

Fig.S6: HMBC spectrum of compound 1 (CD_3OD).

Compound 2

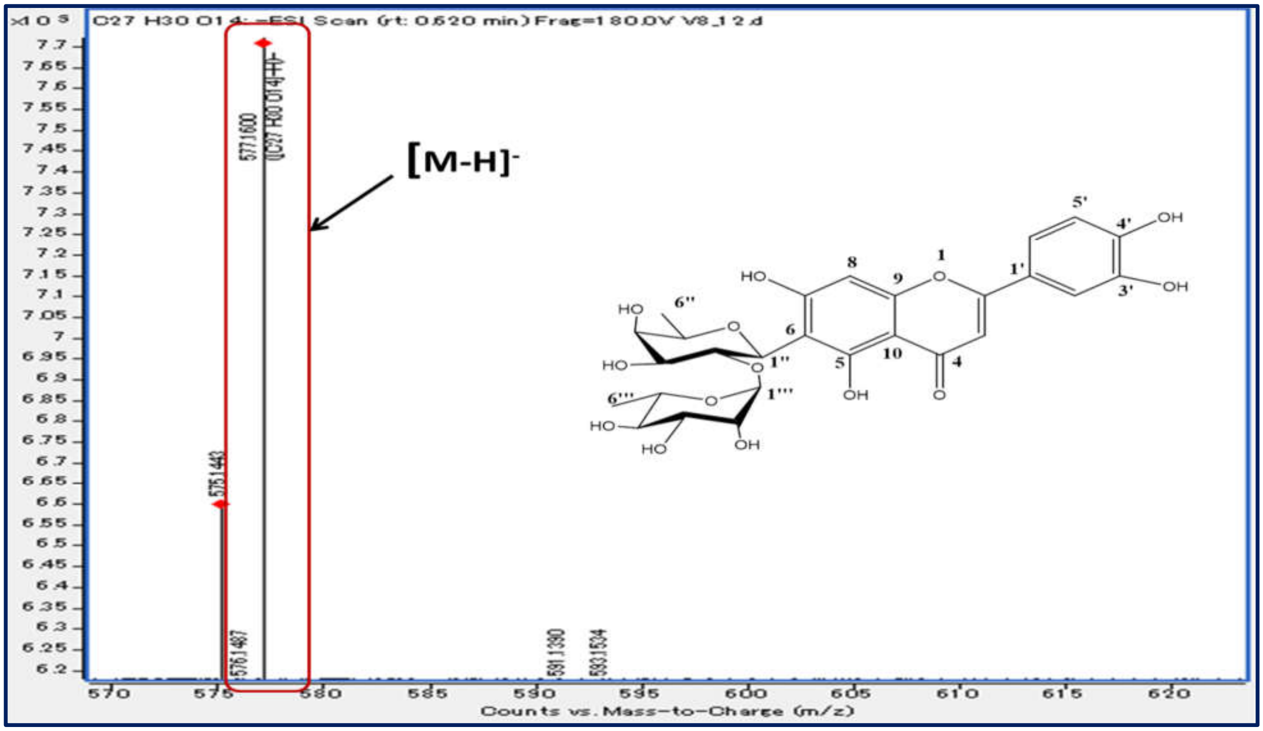

Fig.S7: HRESIMS of compound 2

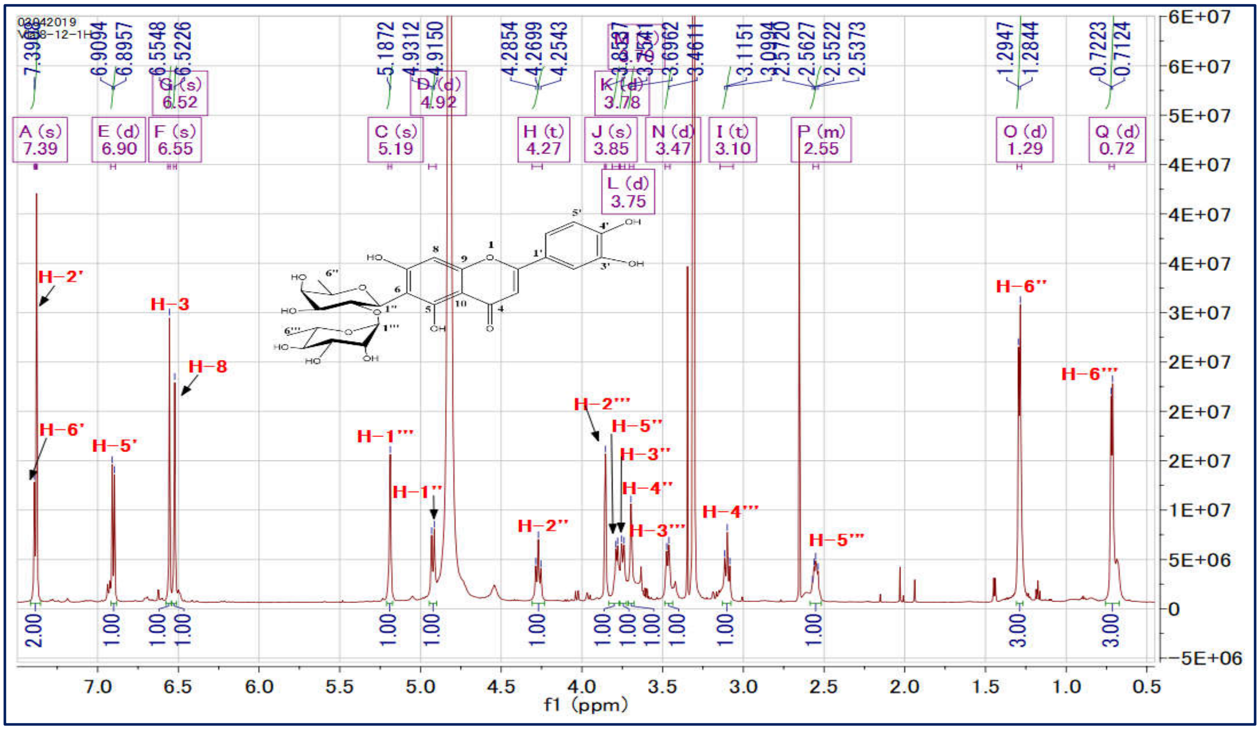

Fig.S8: [1] H-NMR spectrum of compound 2 (CD_3OD).

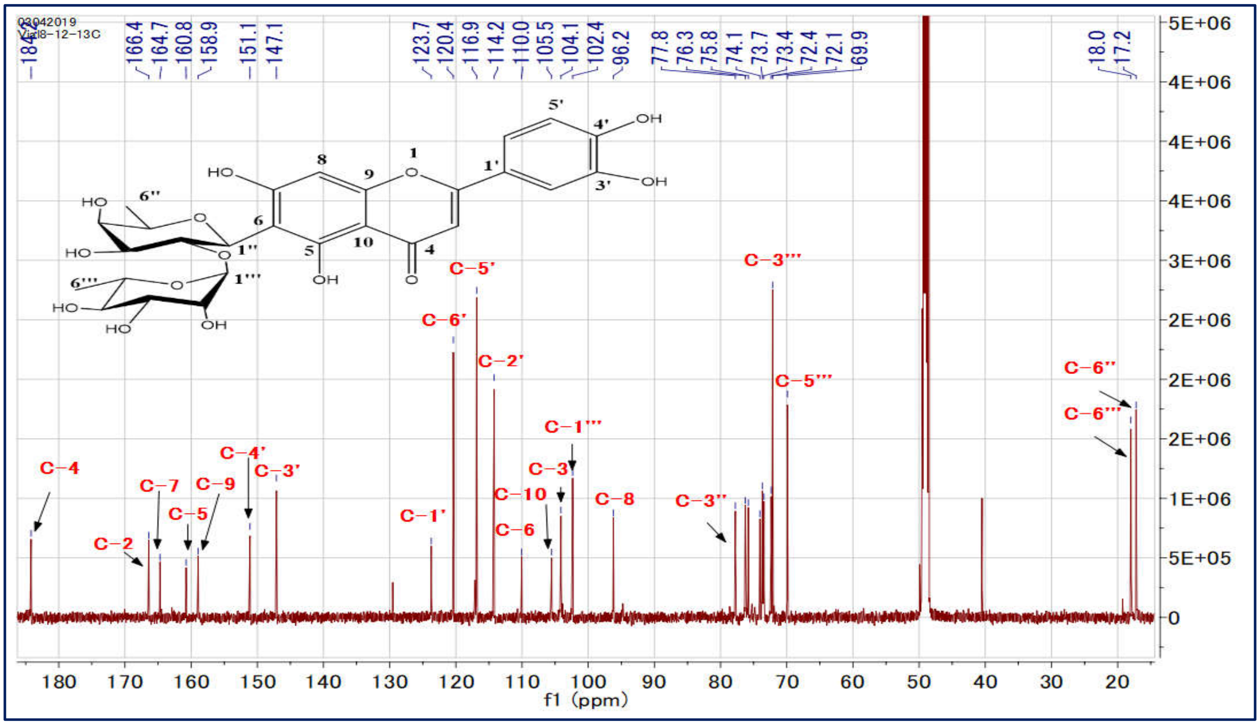

Fig.S9: [13] C-NMR spectrum of compound 2 (CD_3OD).

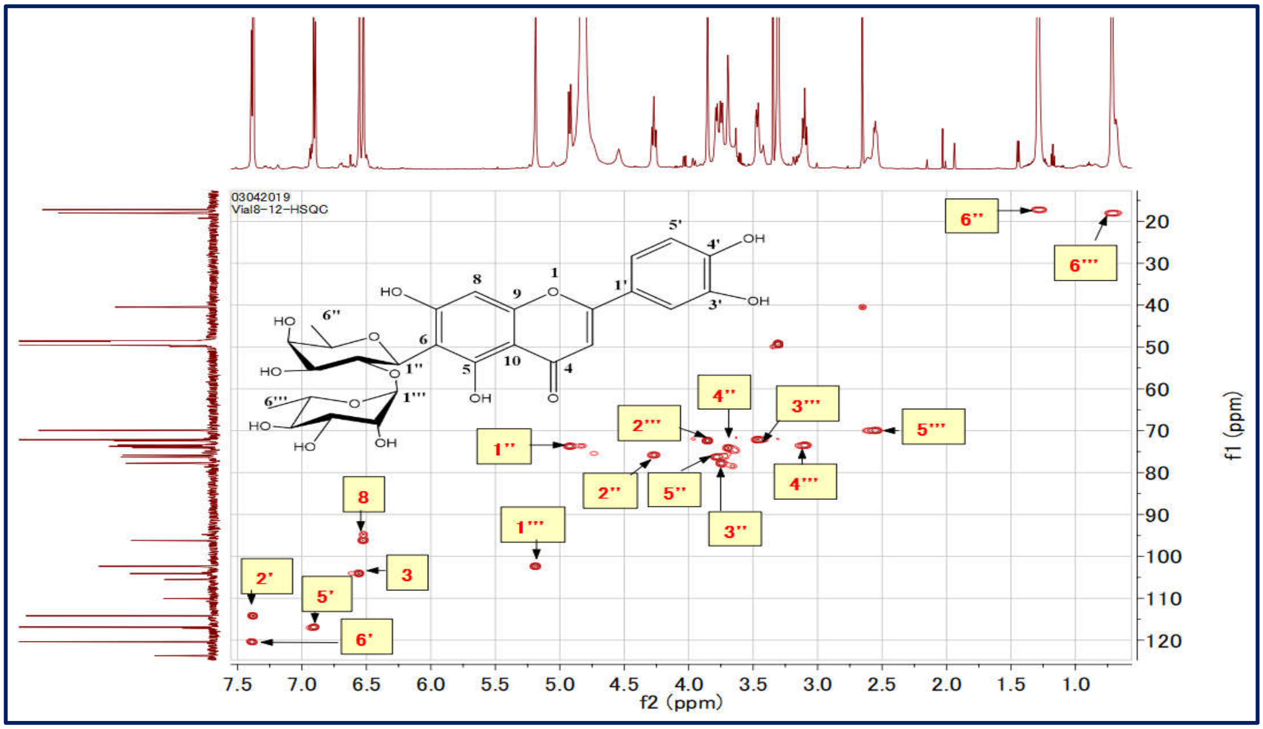

Fig.S10: HSQC spectrum of compound 2 (CD_3OD).

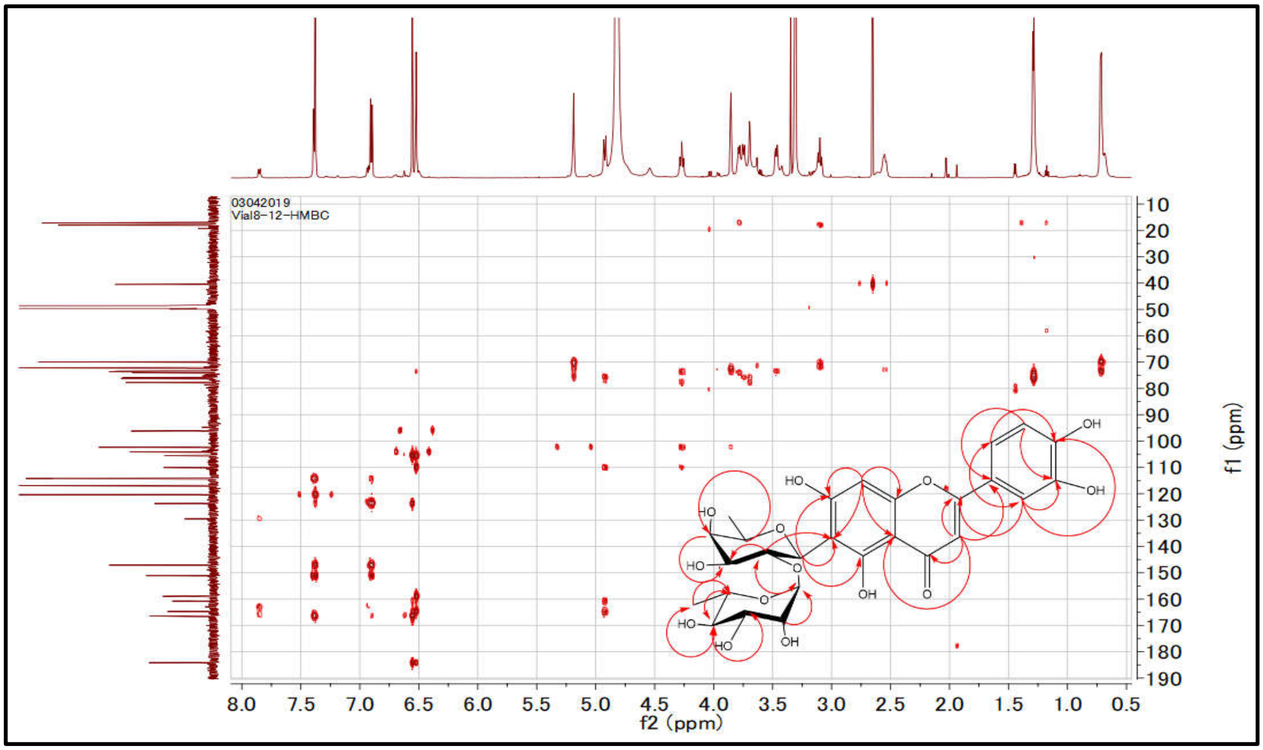

Fig.S11: HMBC spectrum of compound 2 (CD_3OD).

Compound 3

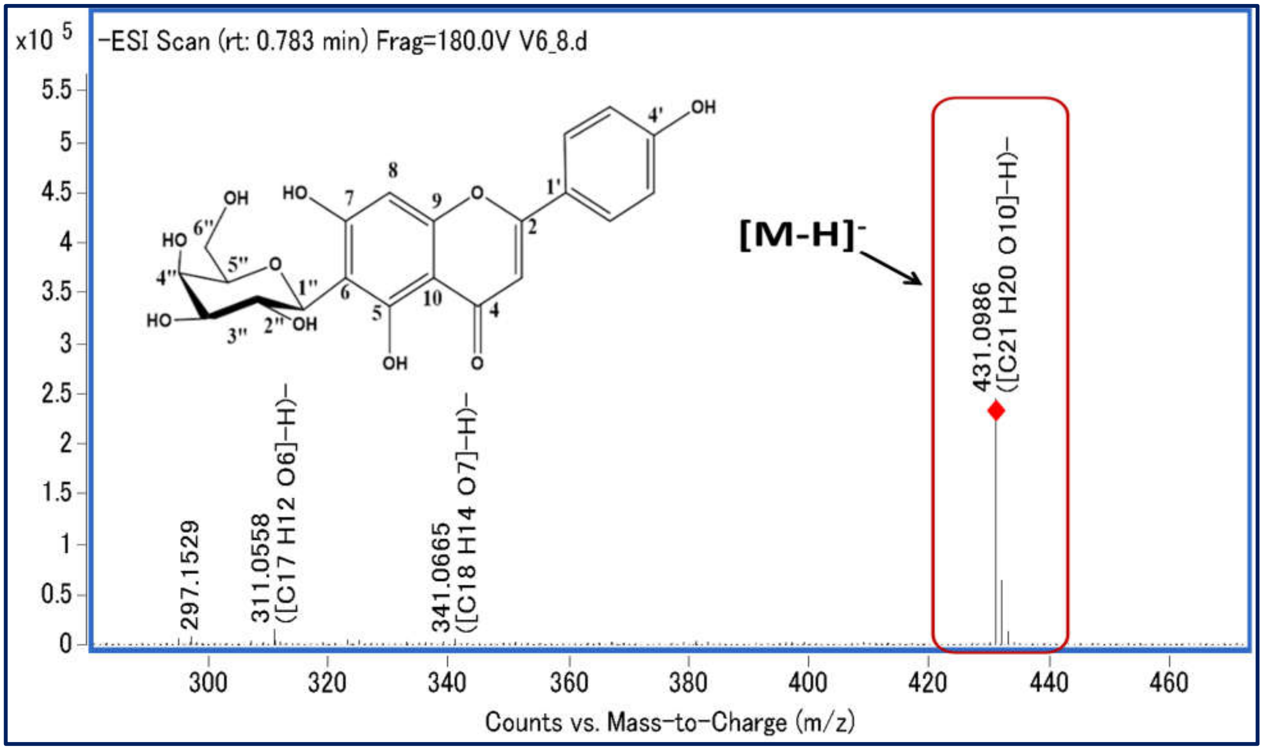

Fig.S12: HRESIMS of compound 3

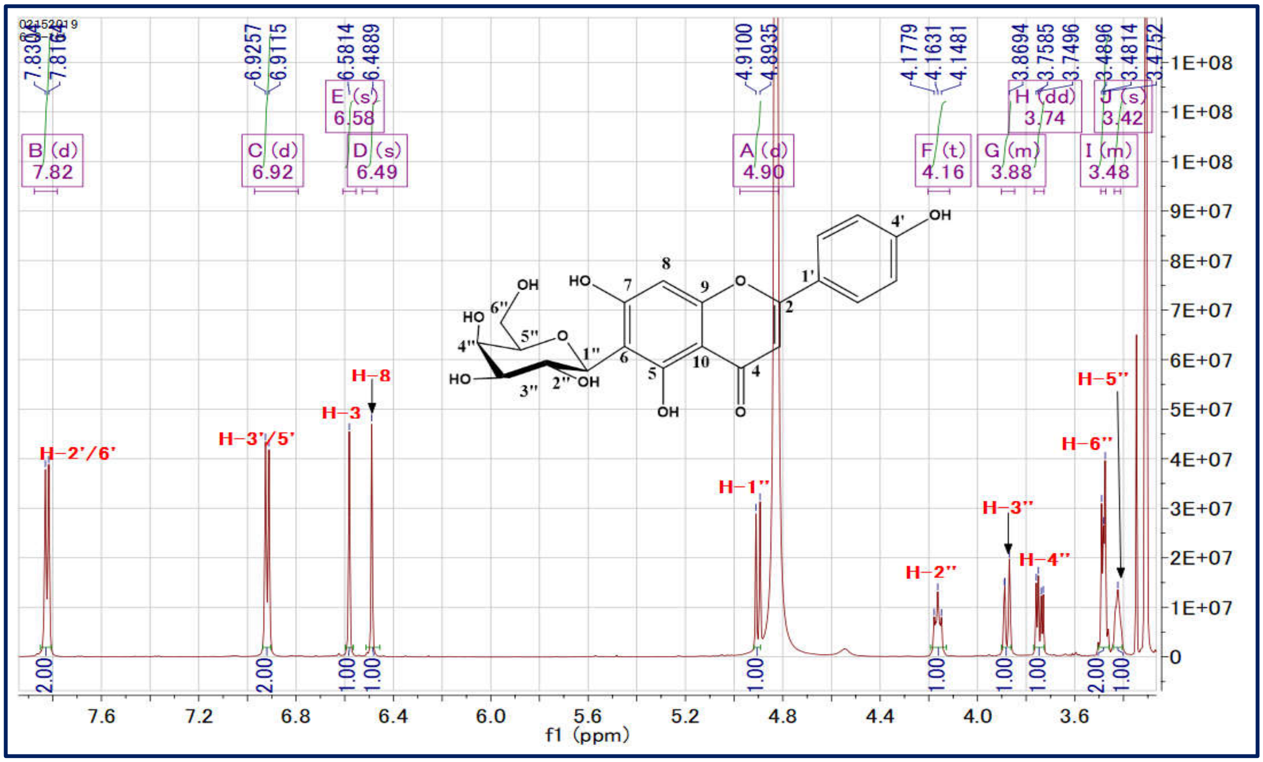

Fig.S13: [1] H-NMR spectrum of compound 3 (CD_3OD).

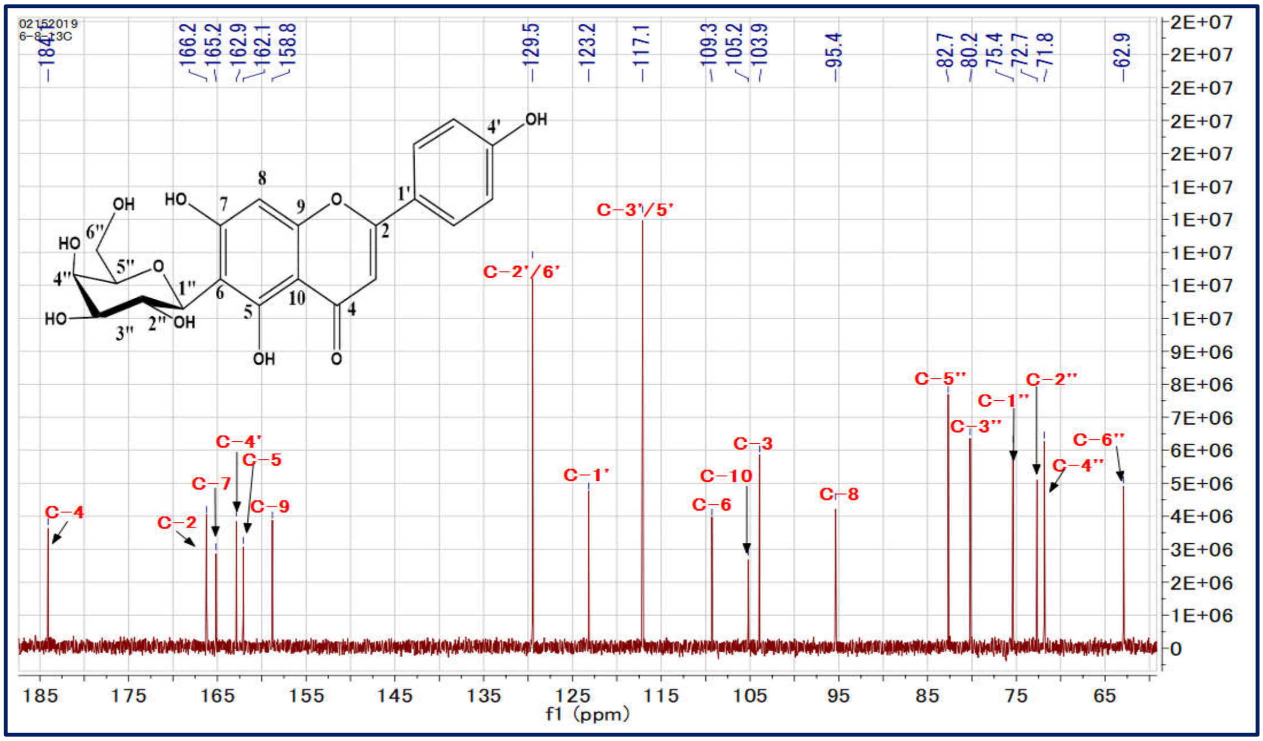

Fig.S14: 13 C-NMR spectrum of compound 3 (CD3OD).

Compound 4

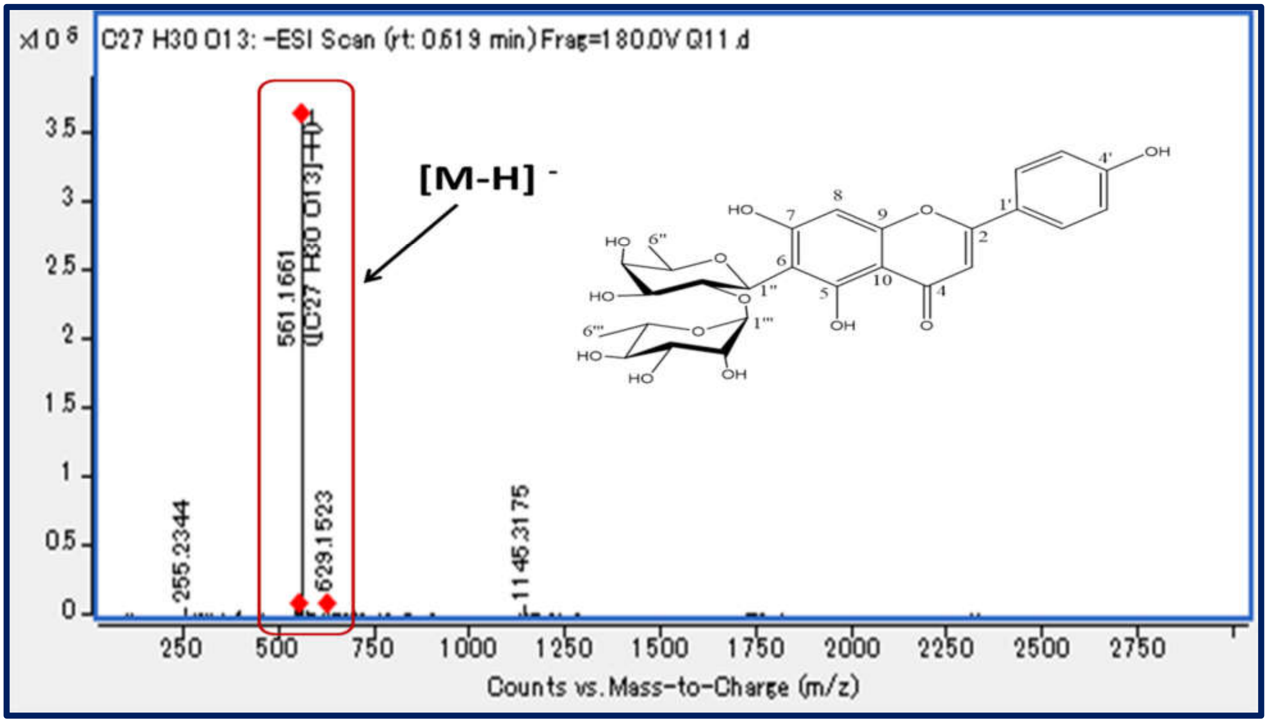

Fig.S15: HRESIMS of compound 4

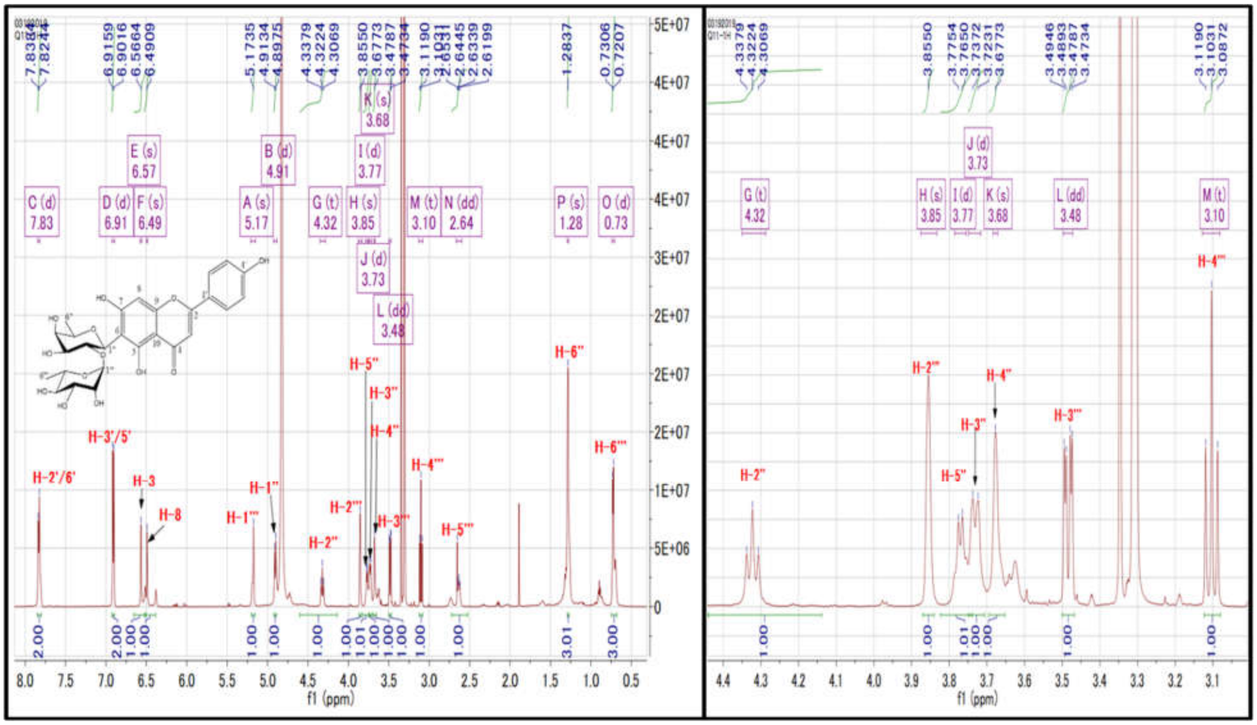

Fig.S16: 1 H-NMR spectrum of compound 4 (CD3OD).

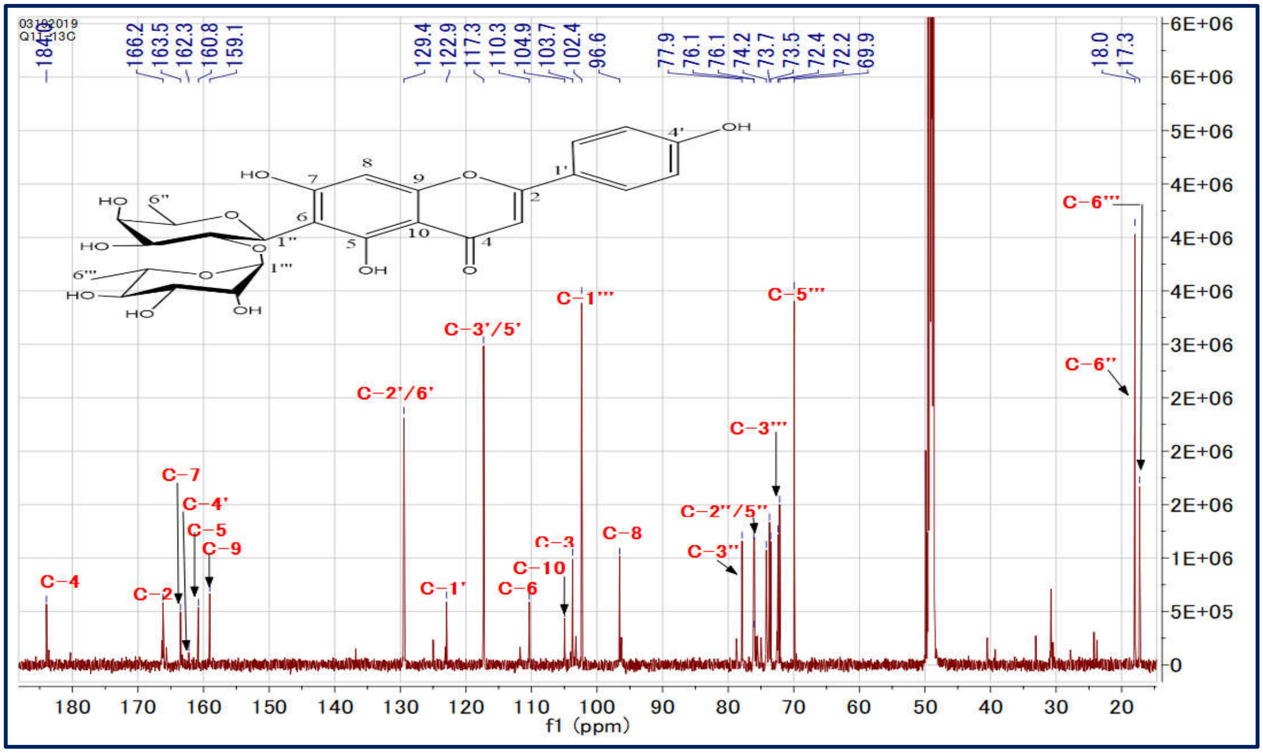

Fig.S17: 13 C-NMR spectrum of compound 4 (CD3OD).

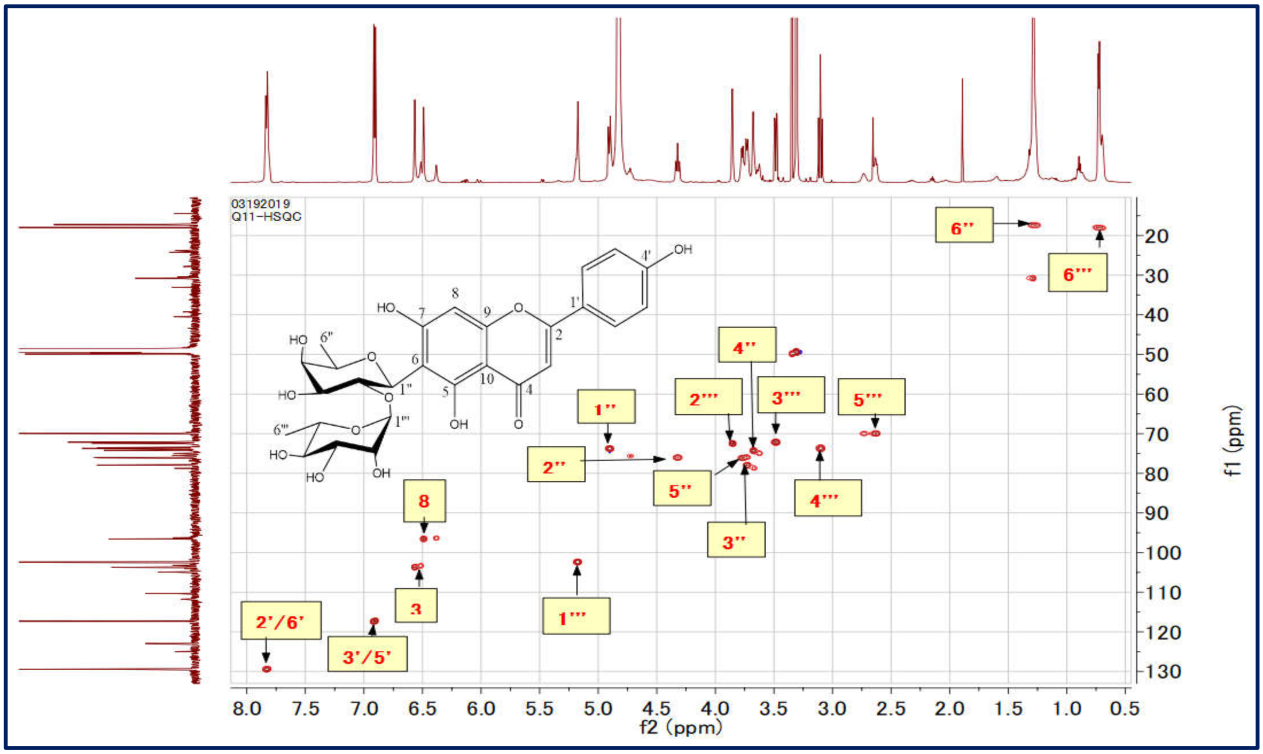

Fig.S18: HSQC spectrum of compound 4 (CD3OD).

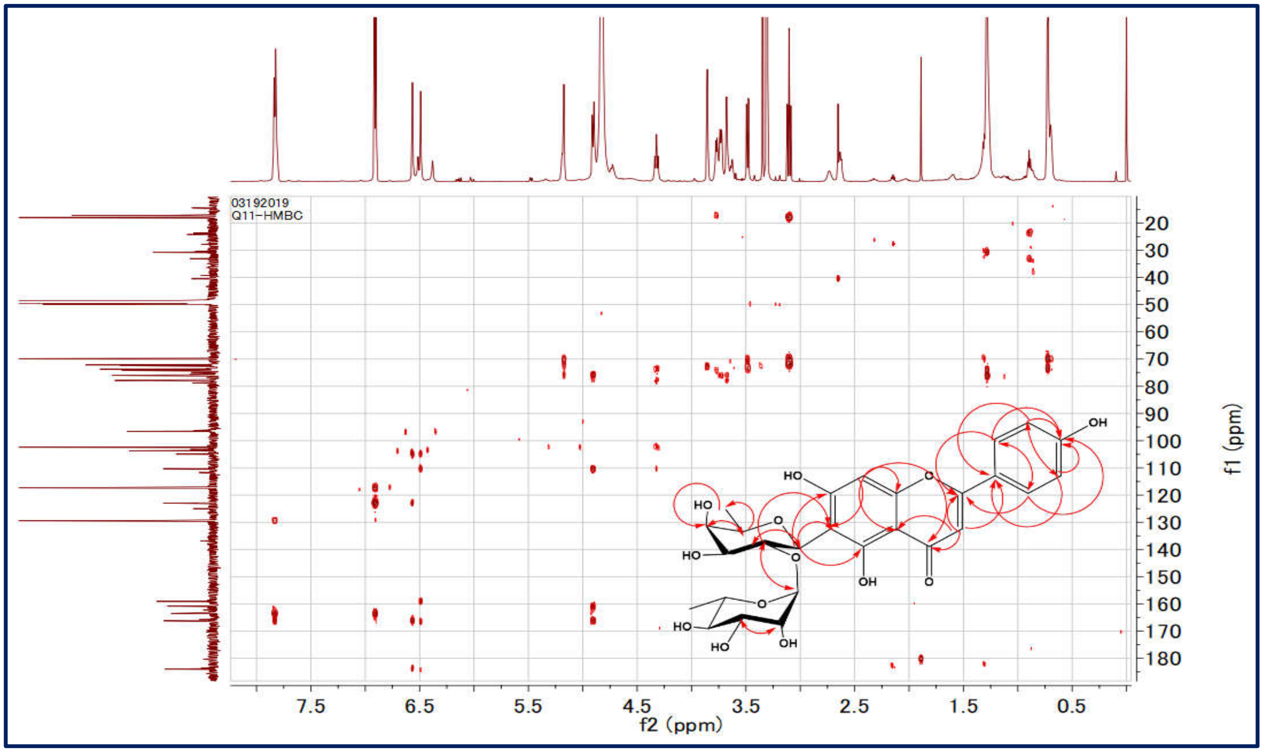

Fig.S19: HMBC spectrum of compound 4 (CD3OD).

Compound 5

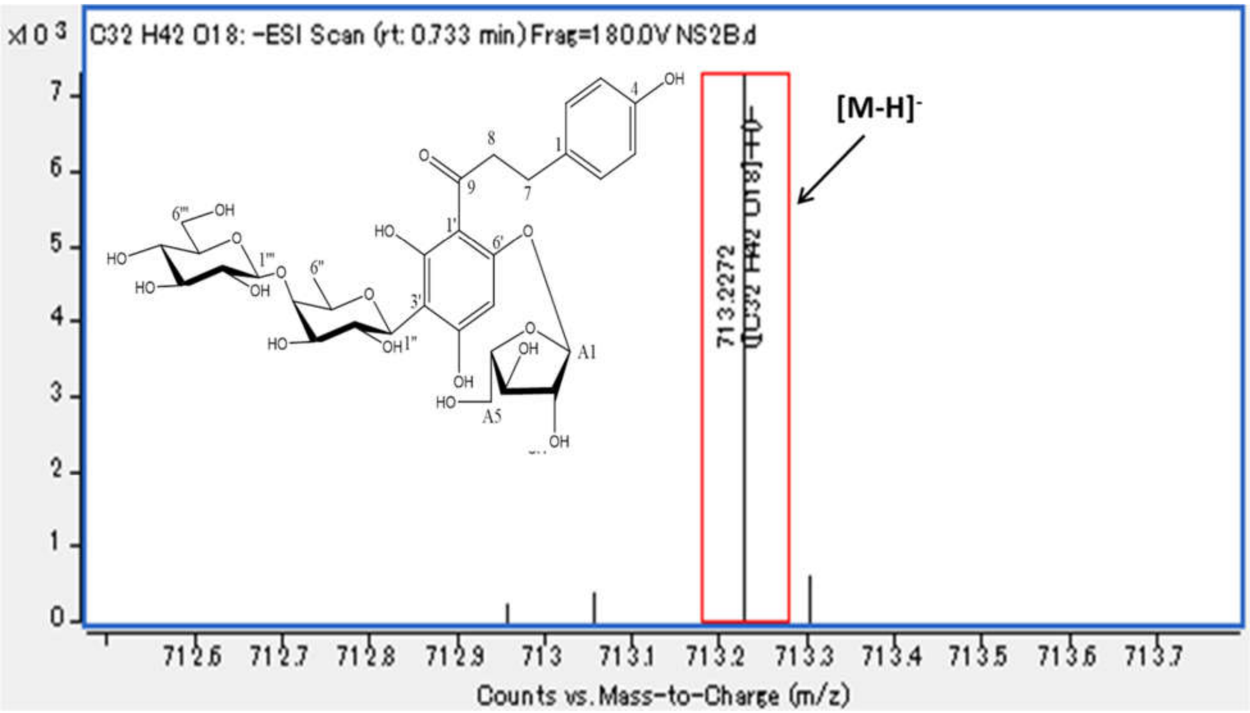

Fig.S20: HRESIMS of compound 5

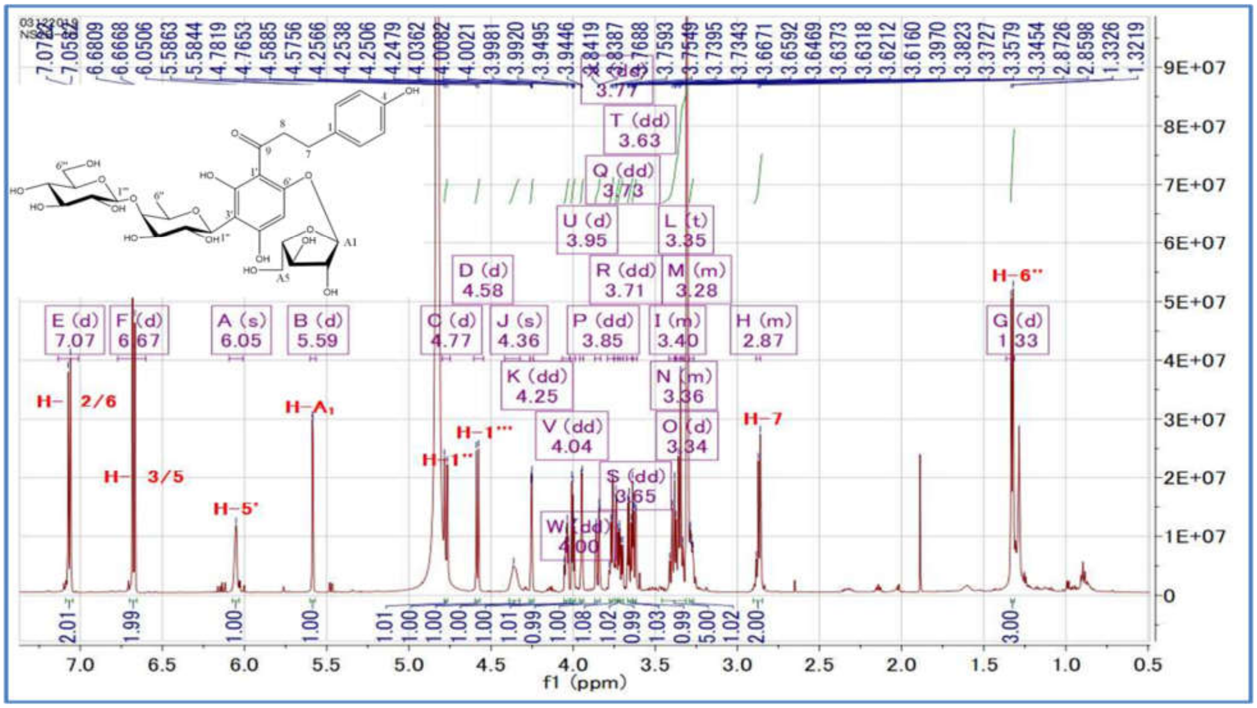

Fig.S21: 1 H-NMR spectrum of compound 5 (CD3OD).

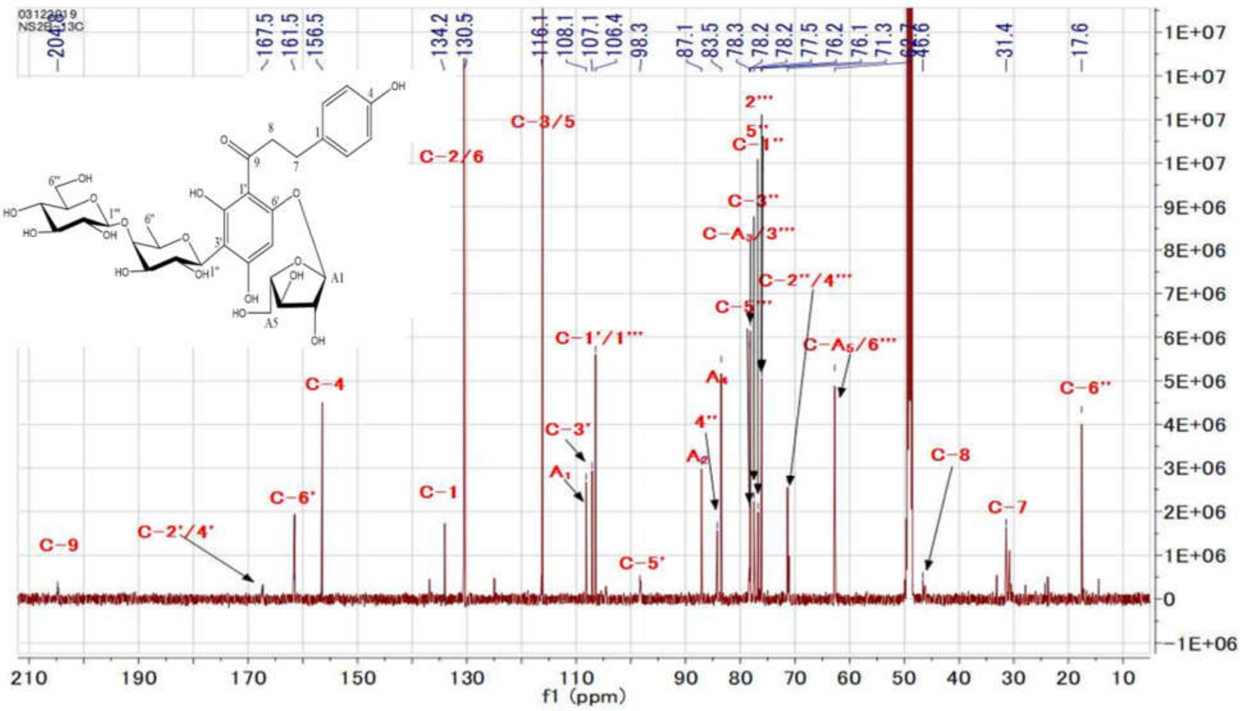

Fig.S22: [13] C-NMR spectrum of compound 5 (CD_3OD).

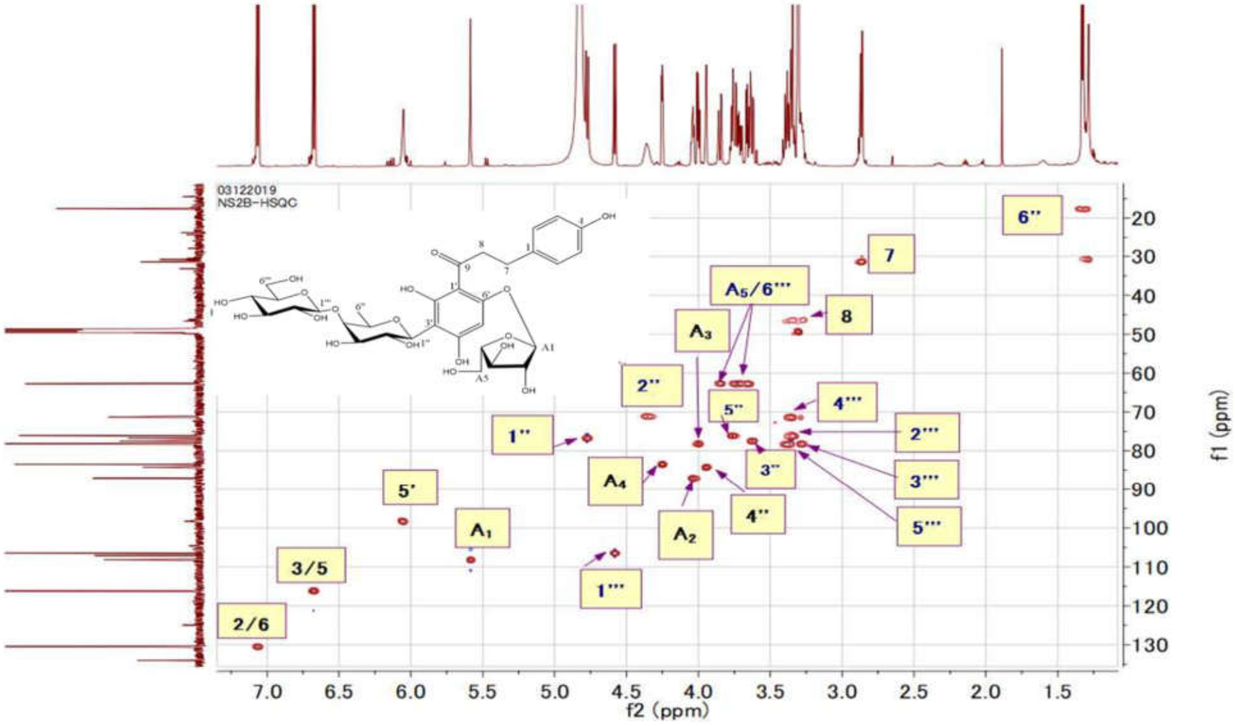

Figure S23: HSQC spectrum of compound 5 (CD3OD).

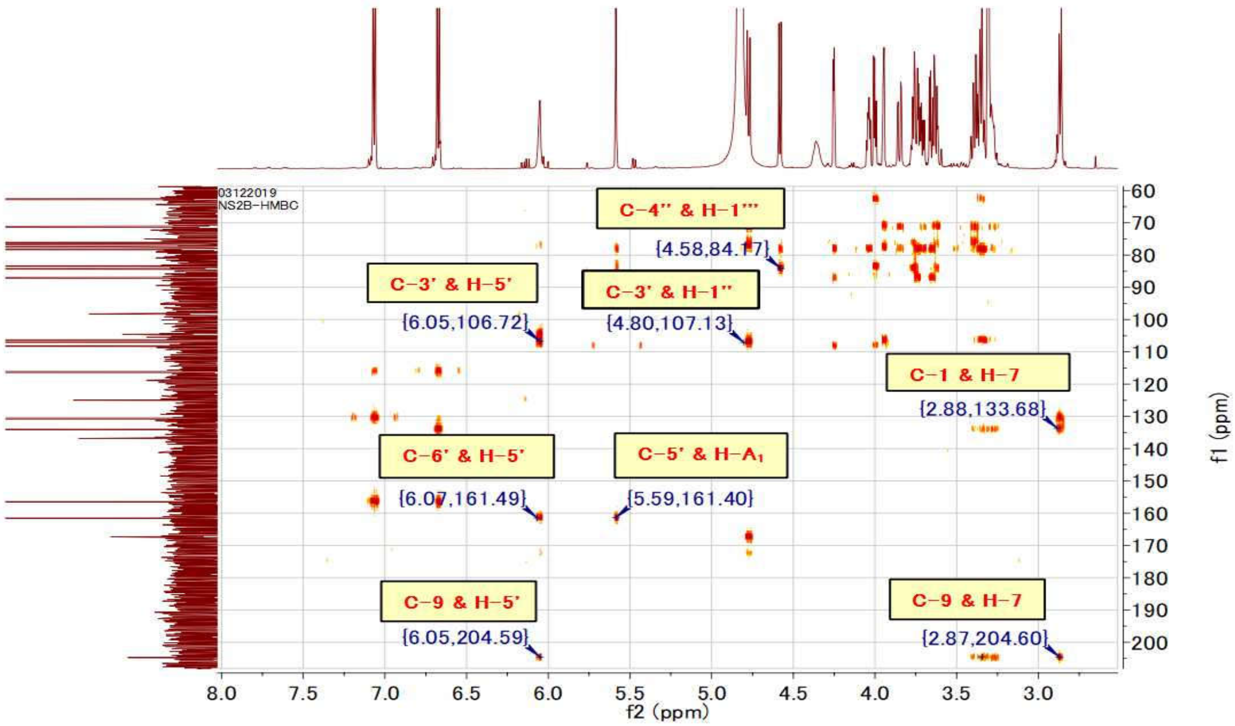

Figure S24: HMBC spectrum of compound 5 (CD3OD).

Compound 6

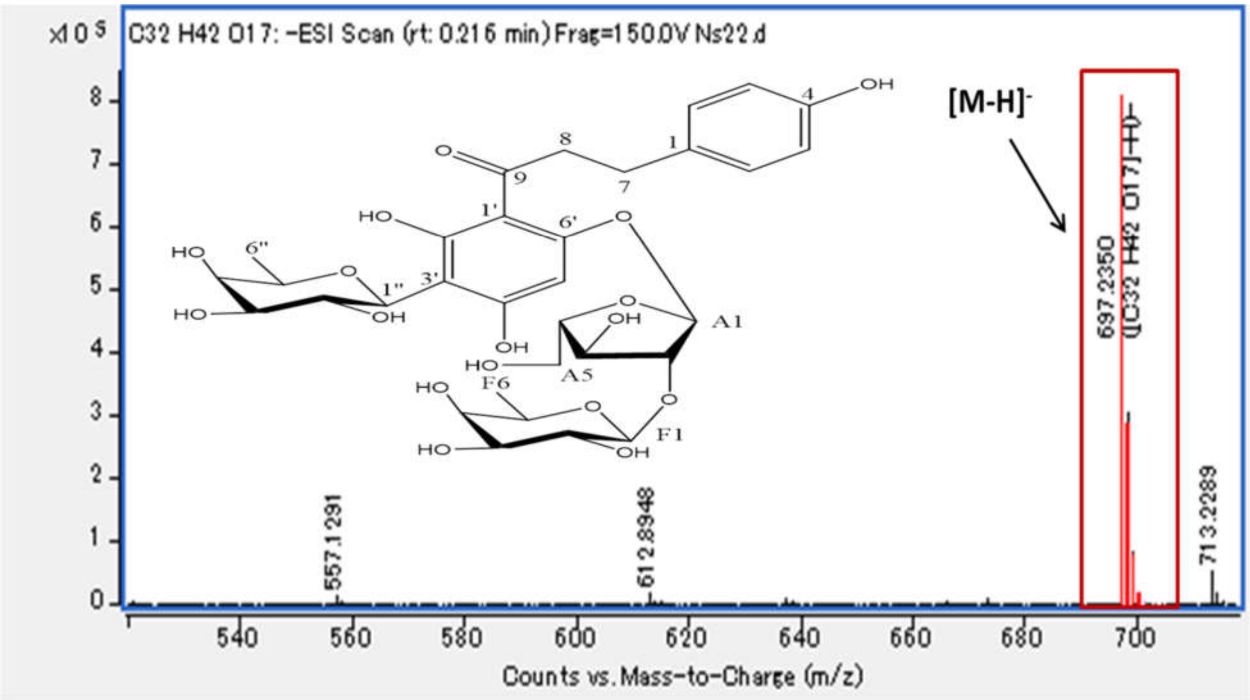

Fig.S25: HRESIMS of compound 6

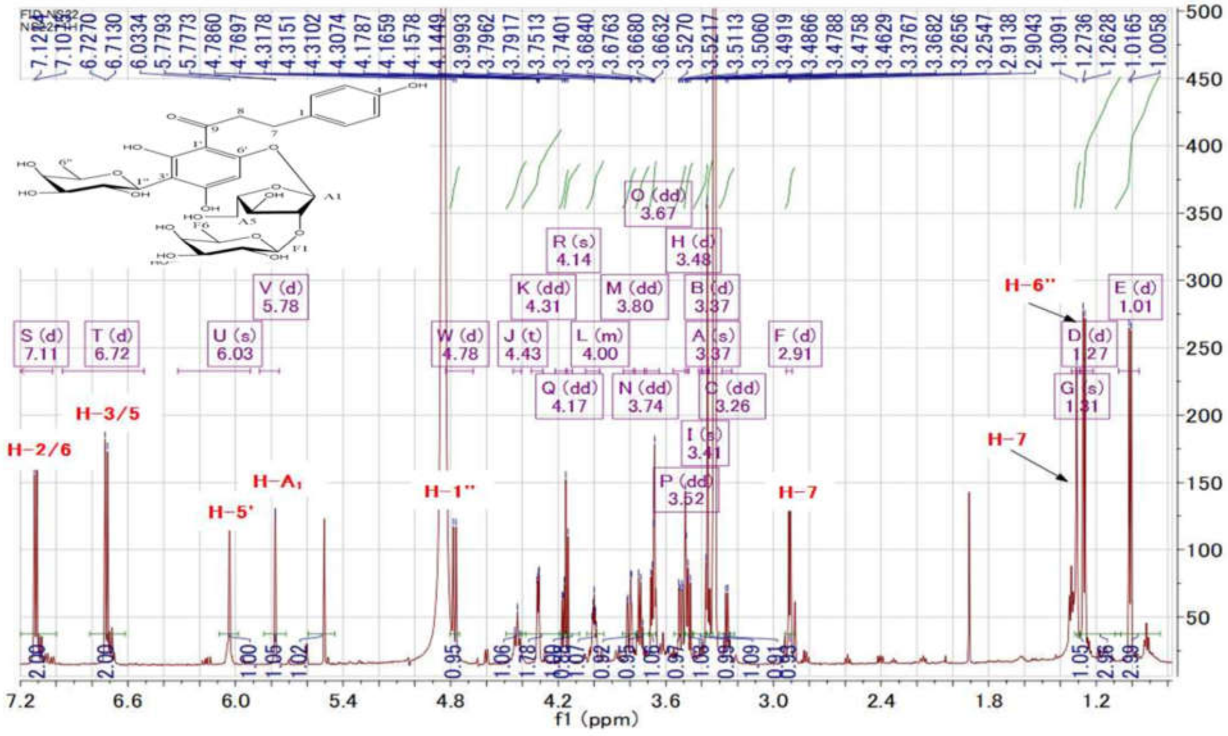

Figure S26: 1 H-NMR spectrum of compound 6 (CD3OD).

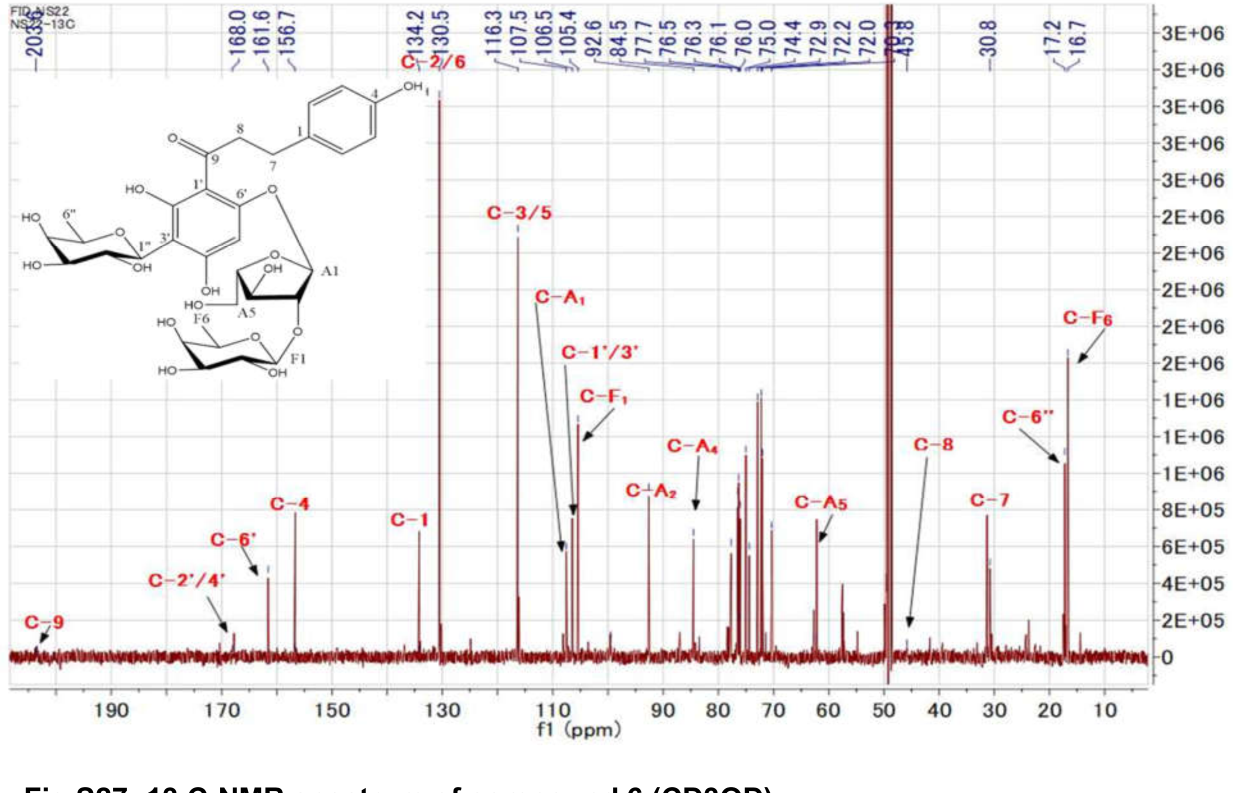

Fig.S27: 13 C-NMR spectrum of compound 6 (CD3OD).

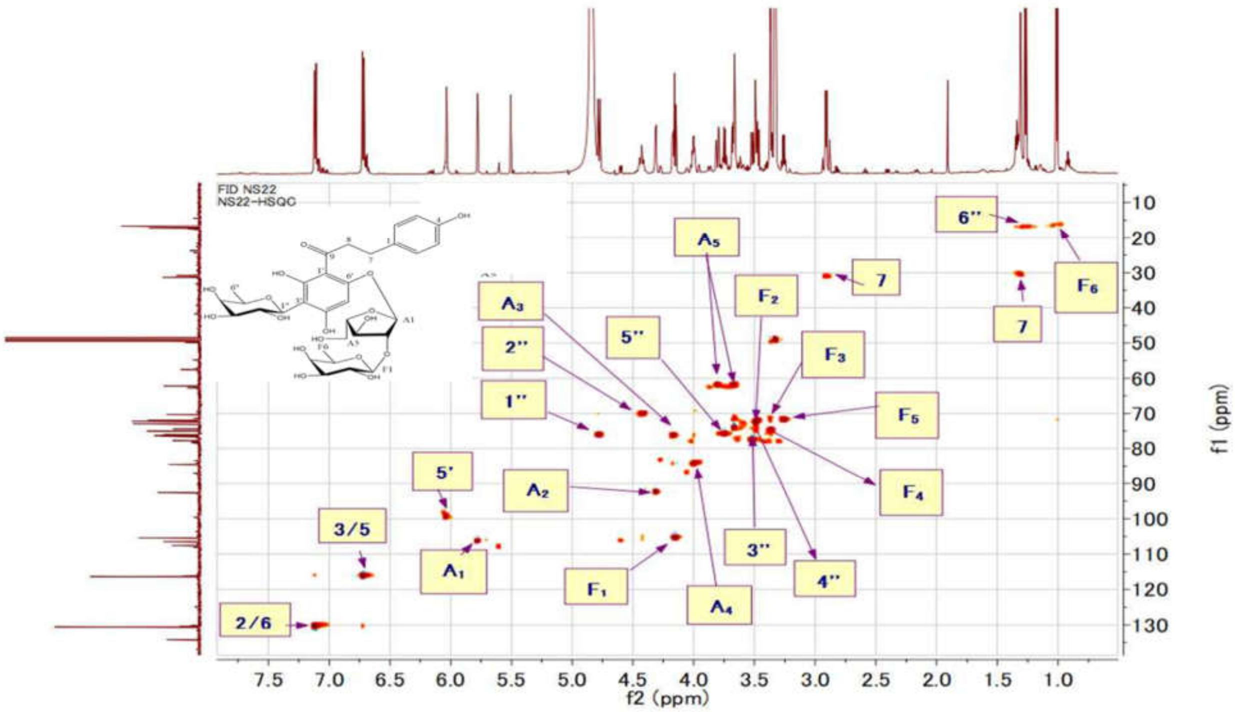

Figure S28: HSQC spectrum of compound 6 (CD_3OD).

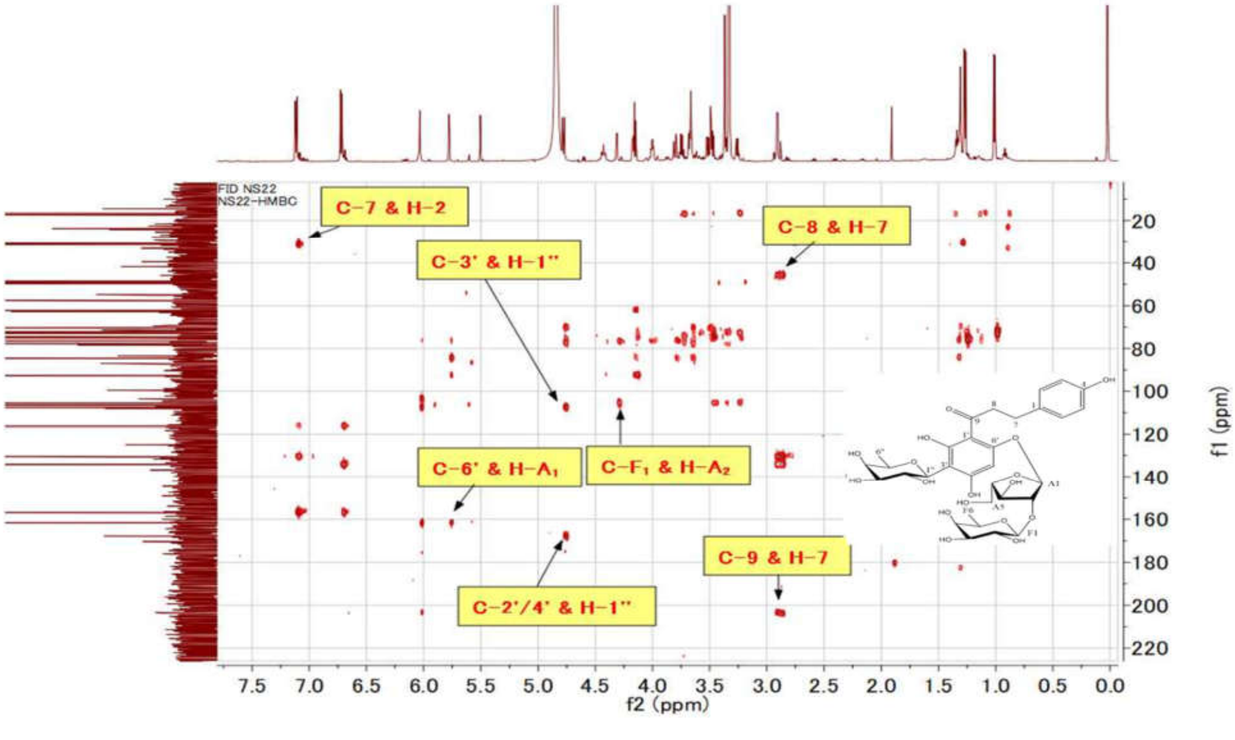

Figure S29: HMBC spectrum of compound 6 (CD3OD).

Compound 7

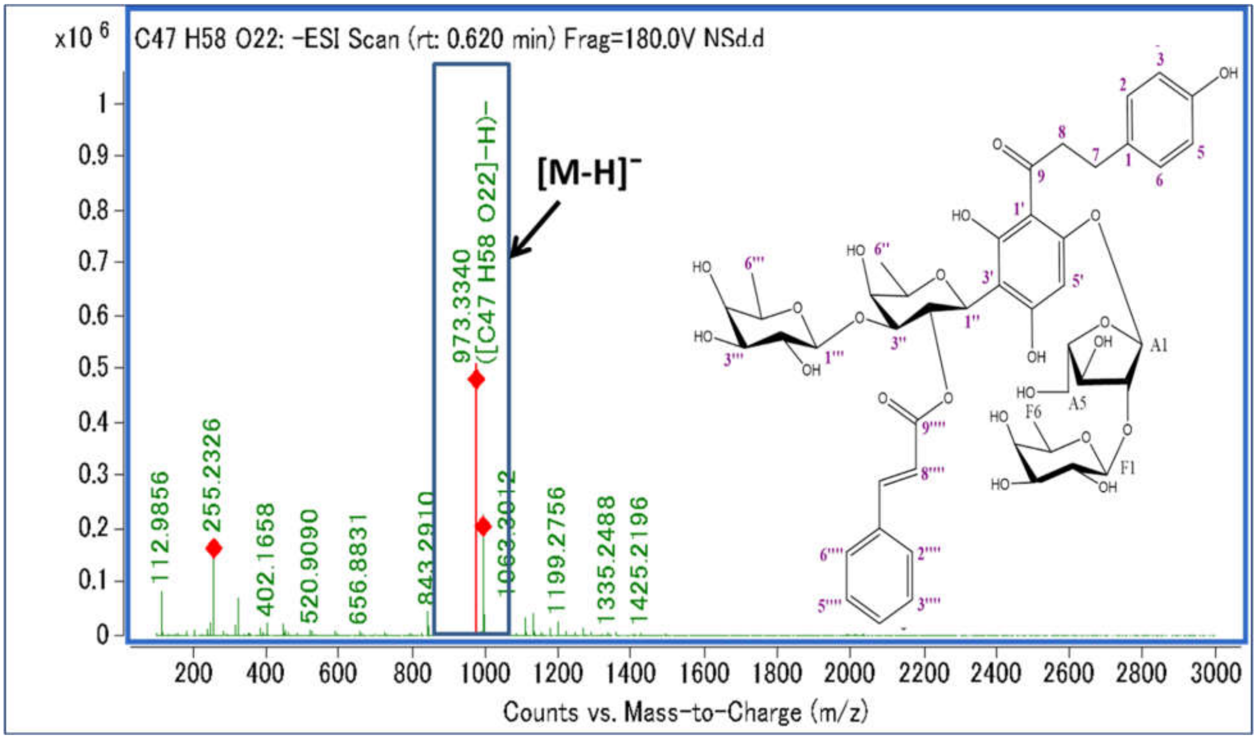

Fig.S30: HRESIMS of compound 7

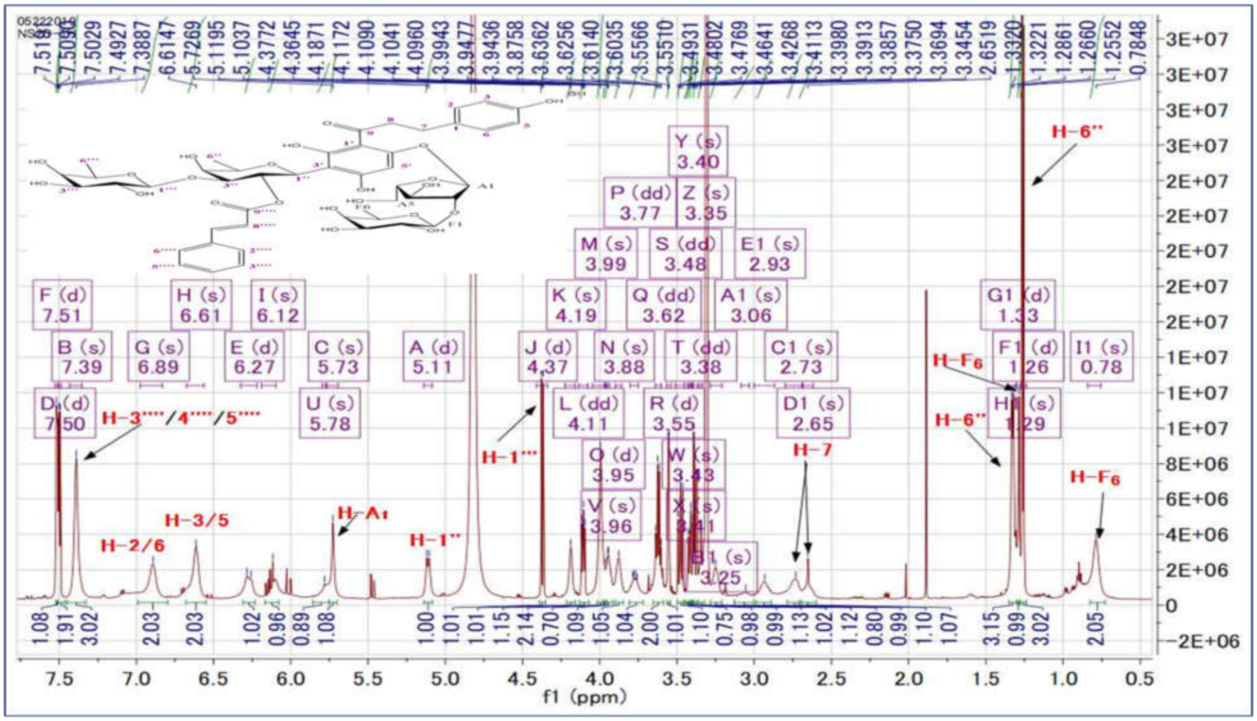

Fig.S31: ^{1} H-NMR spectrum of compound 7 (CD_3OD).

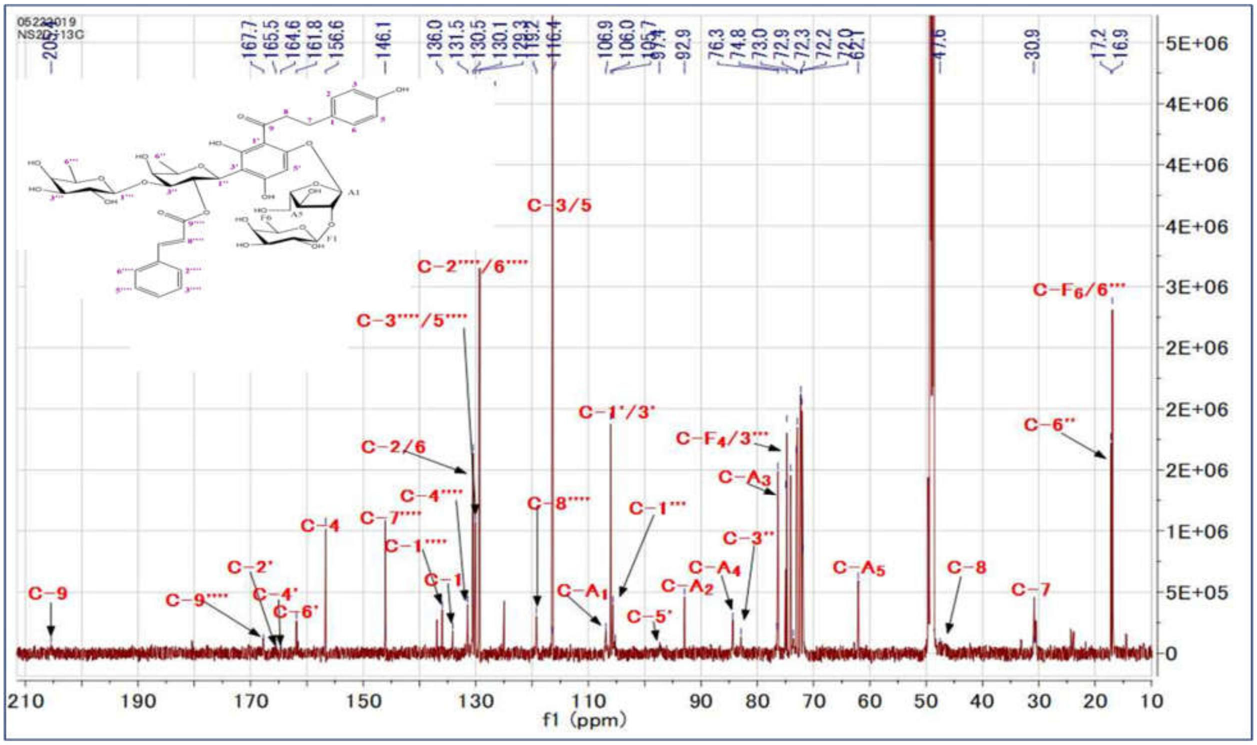

Fig.S32: 13 C-NMR spectrum of compound 7 (CD3OD).

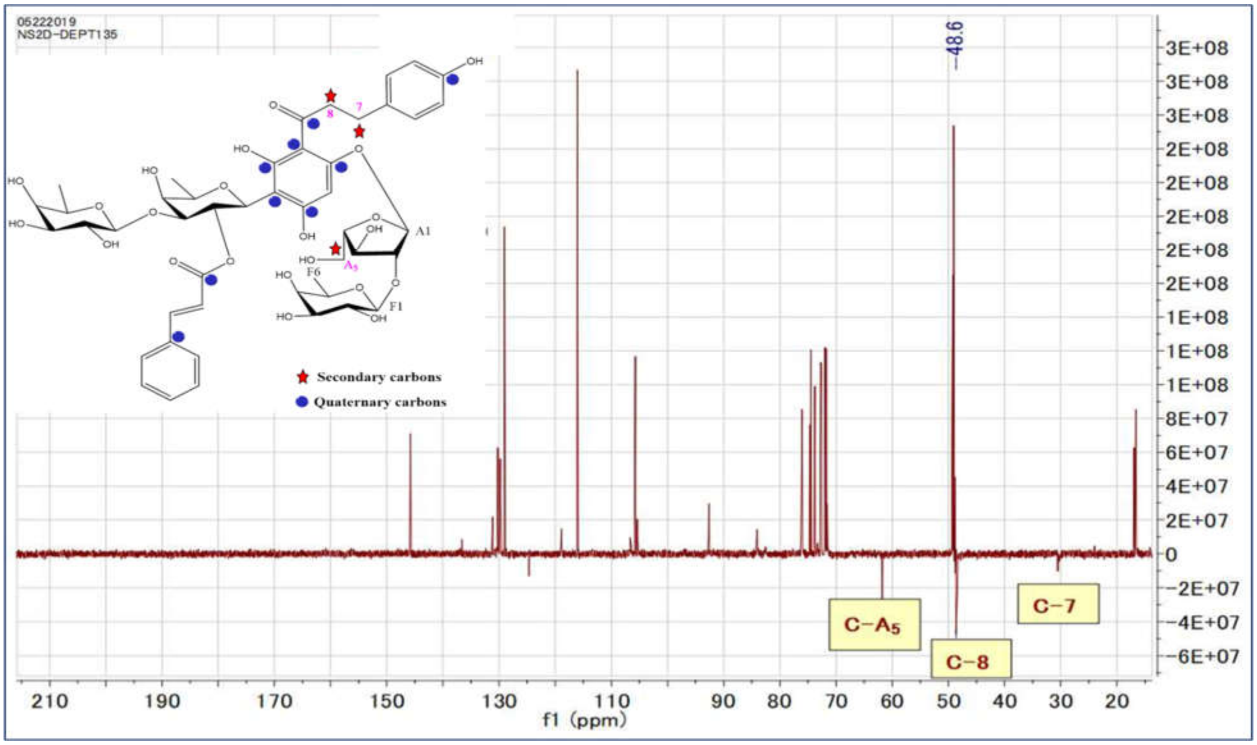

Fig.S33: DEPT-135 spectrum of compound 7 (CD_3OD).

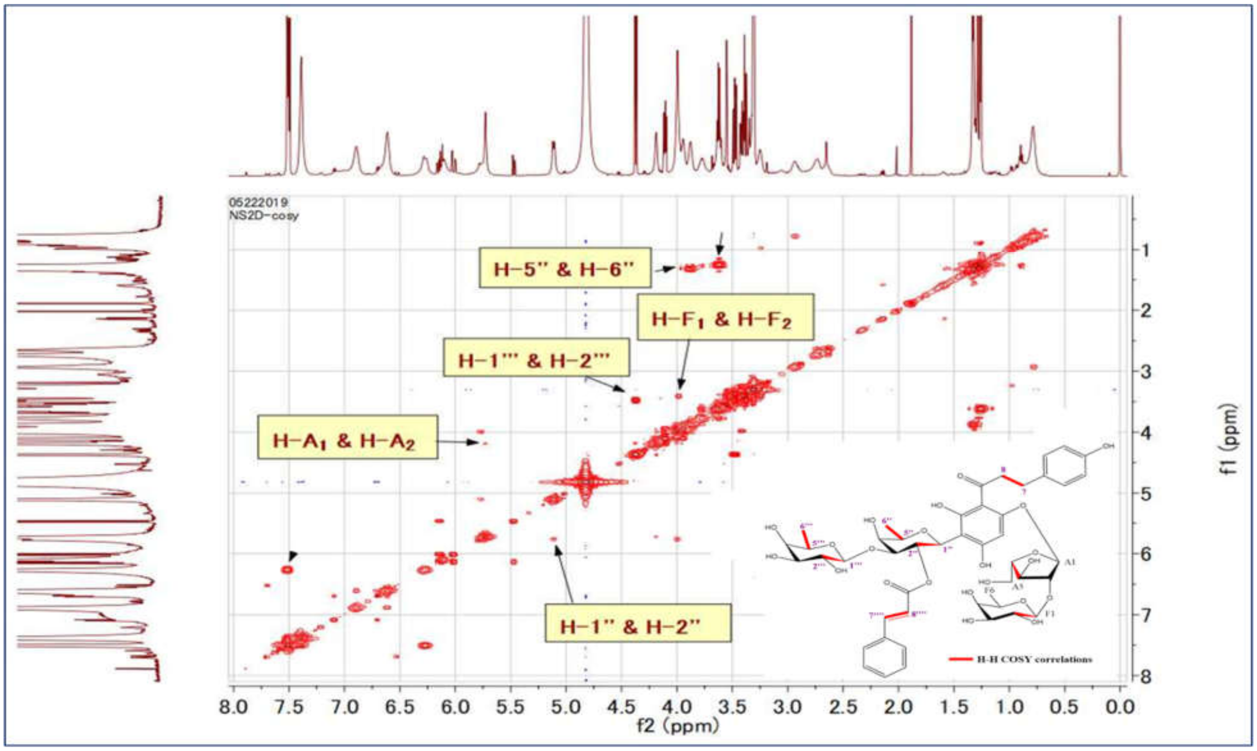

Fig.S34: ^{1}H-^{1}H COSY spectrum of compound 7 (CD_3OD).

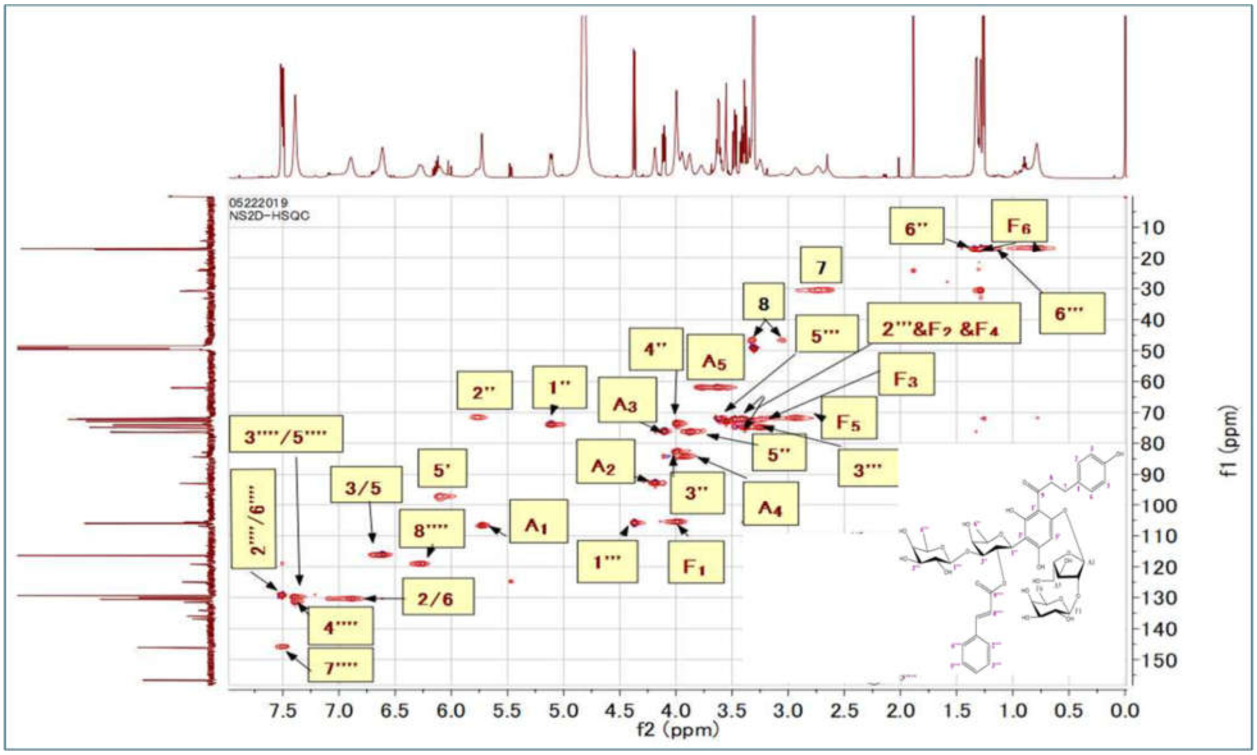

Fig.S35: HSQC spectrum of compound 7 (CD_3OD).

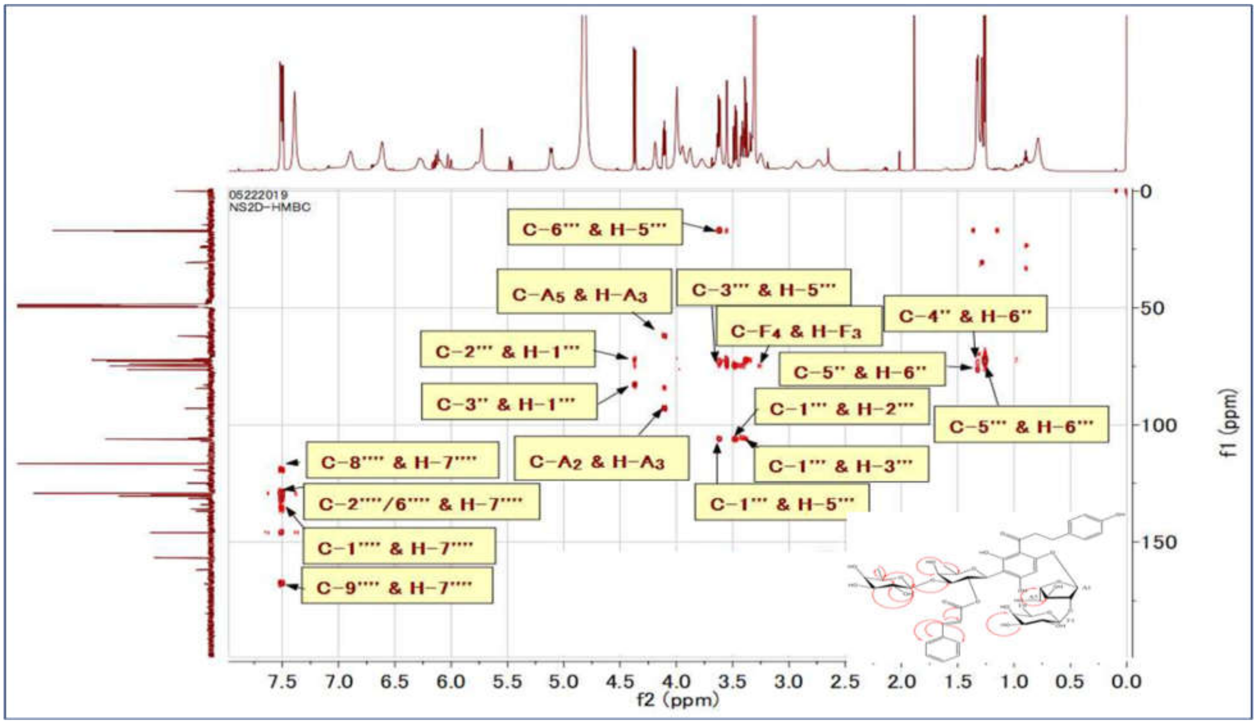

Fig.S36: HMBC spectrum of compound 7 (CD3OD).

Compound 8

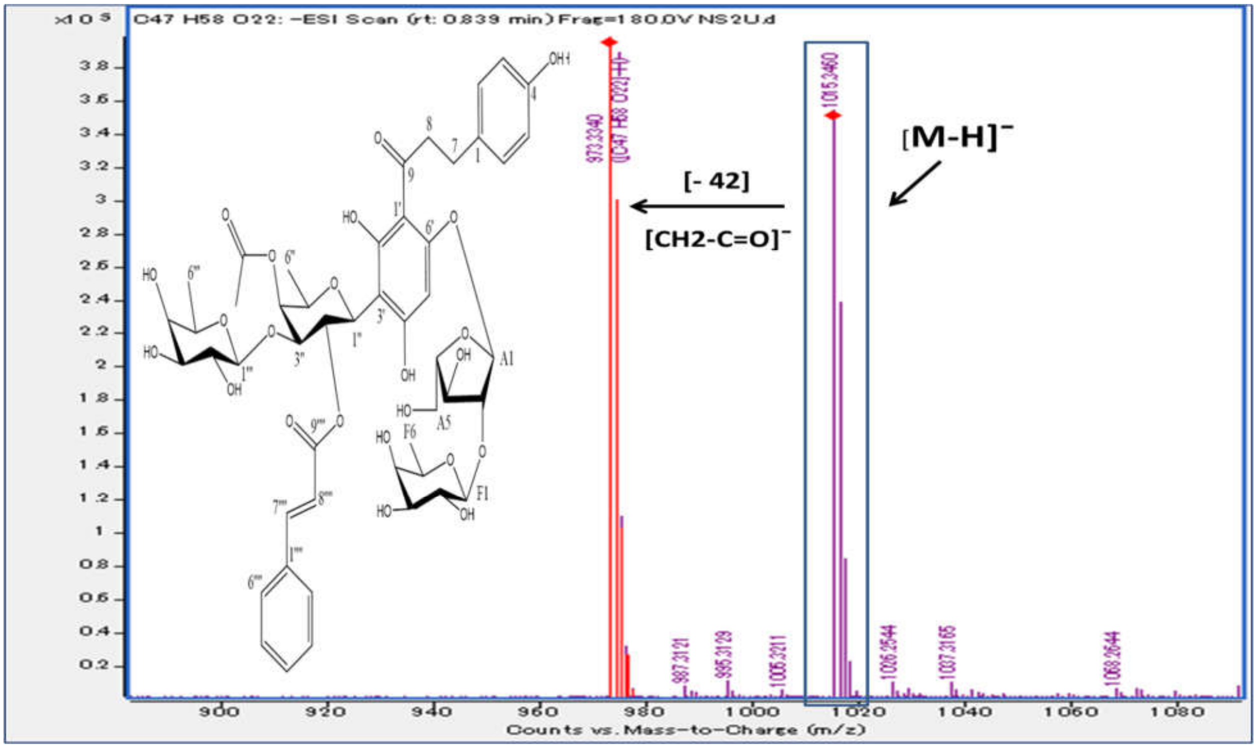

Fig.S37: HRESIMS of compound 8

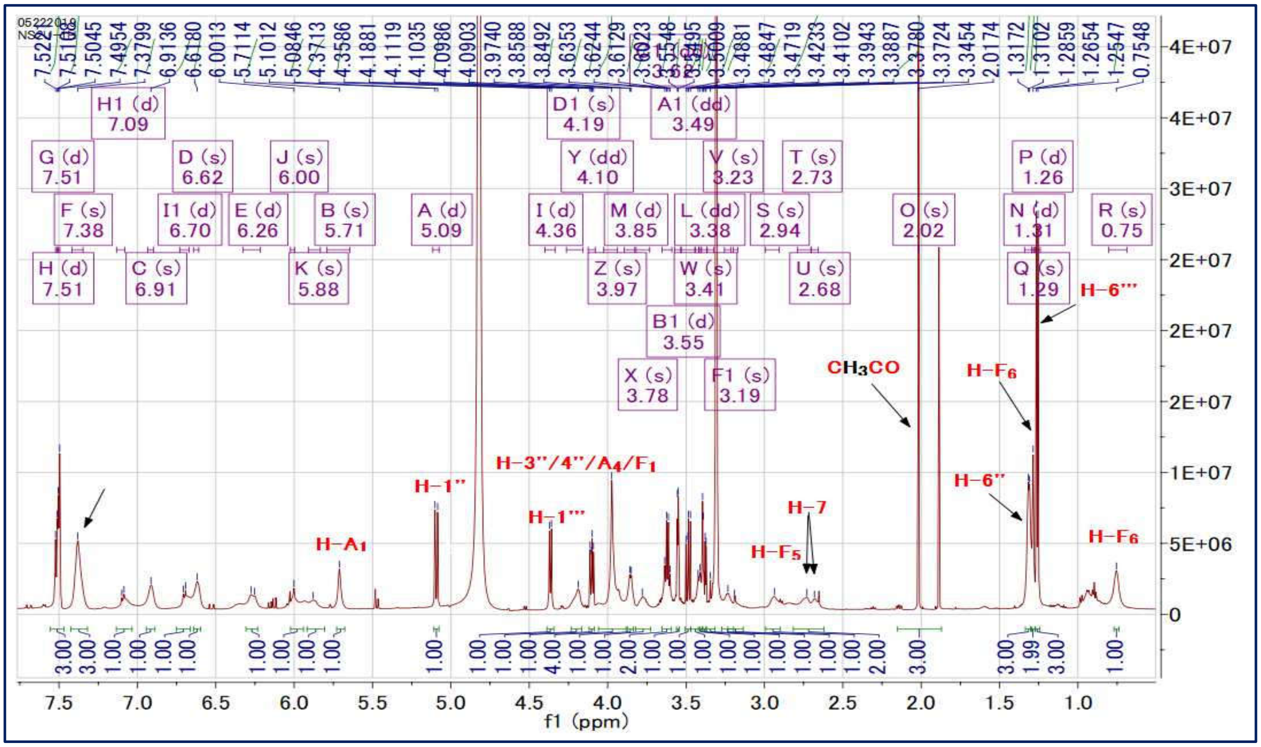

Fig.S38: [1] H-NMR spectrum of compound 8 (CD$_3$OD).

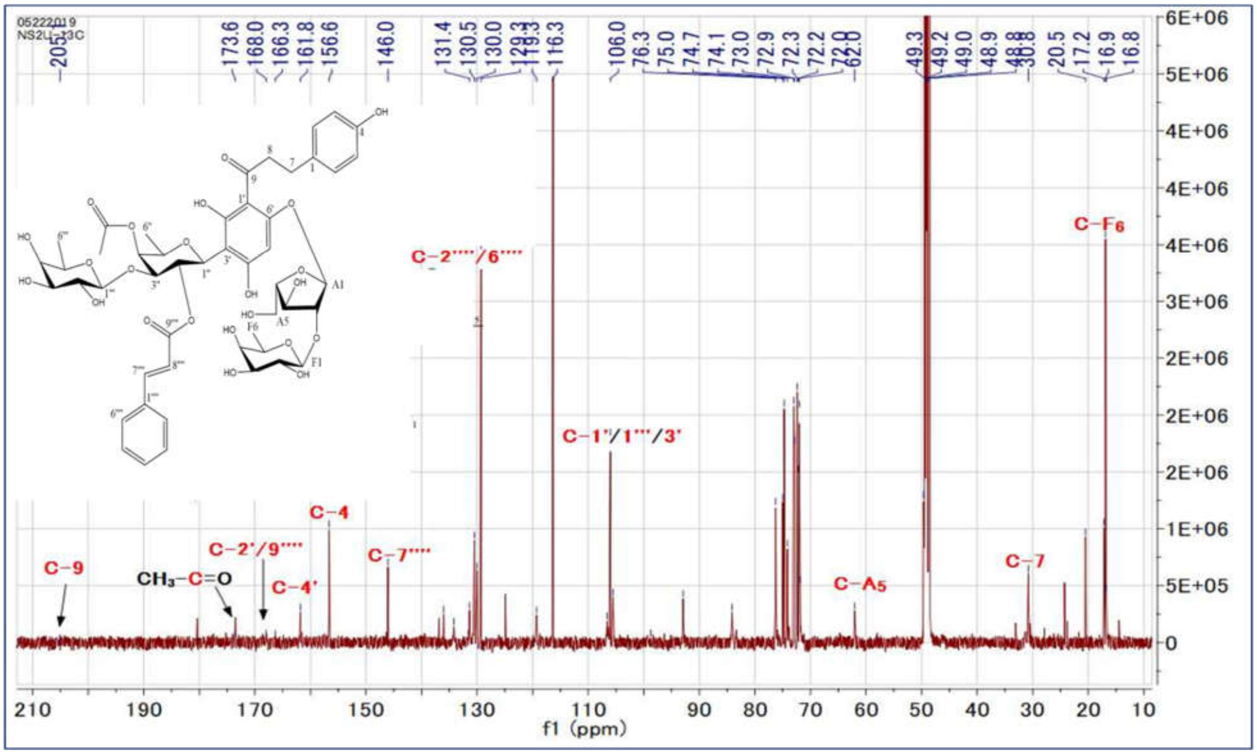

Figure S39: [13] C-NMR spectrum of compound 8 (CD$_3$OD).

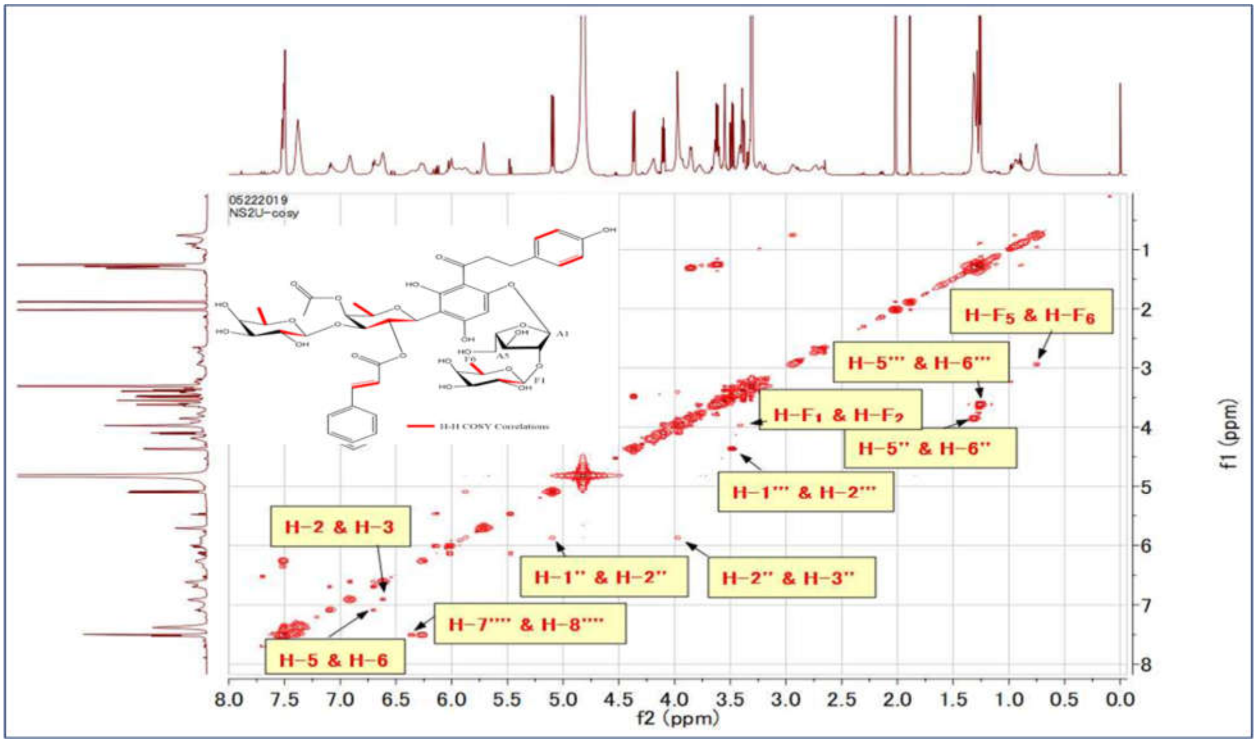

Figure S41: ^{1}H-^{1}H COSY spectrum of compound 8 (CD_3OD).

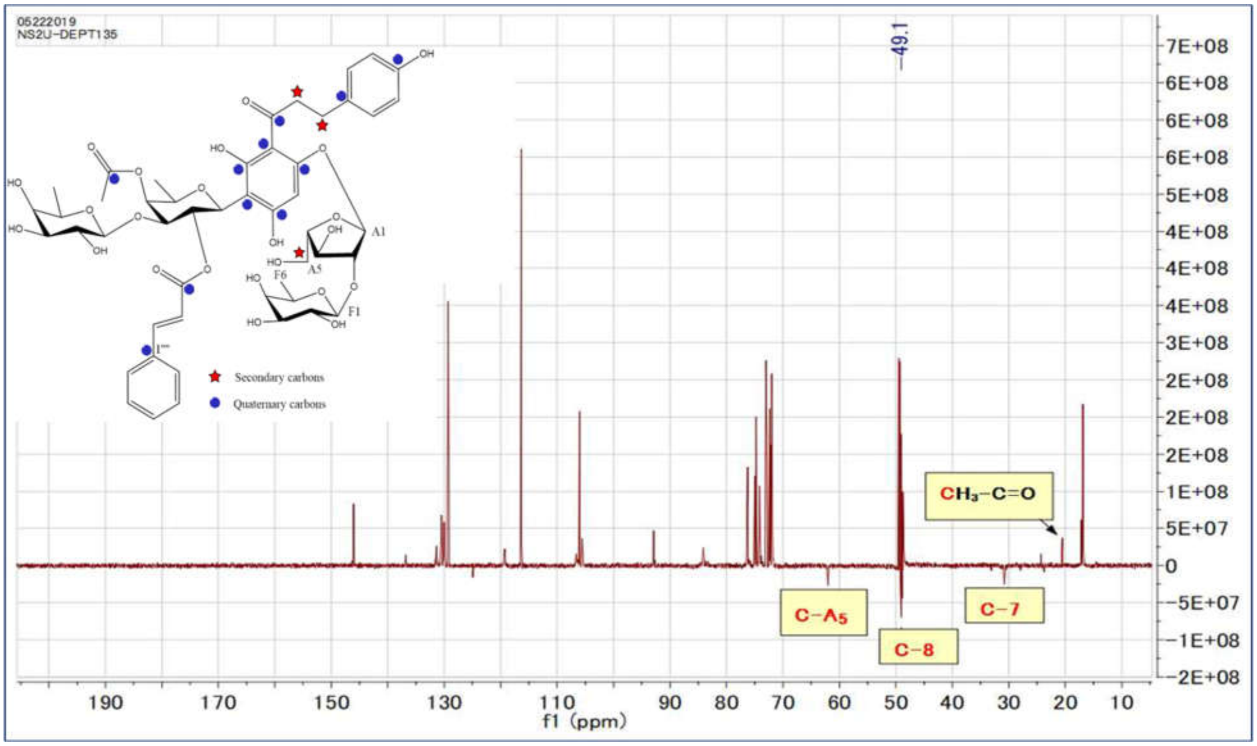

Figure S40: DEPT-135 spectrum of compound 8 (CD_3OD).

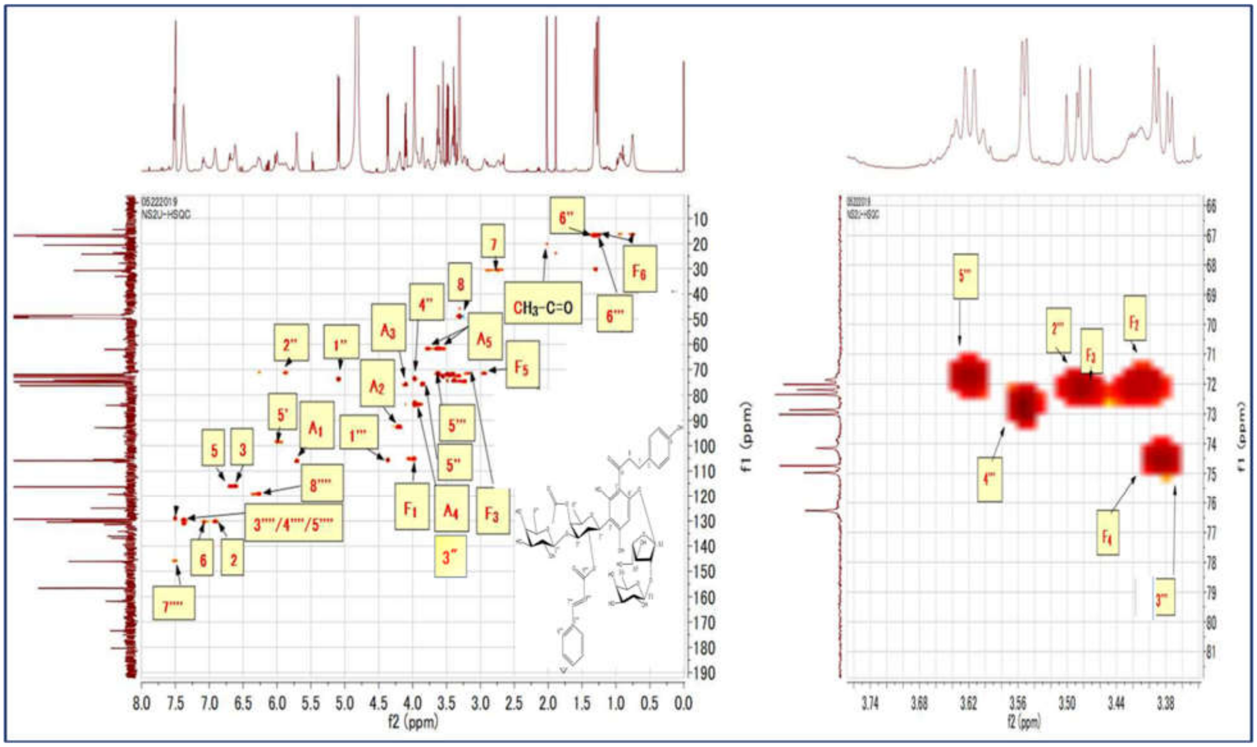

Figure S42: HSQC spectrum of compound 8 (CD_3OD).

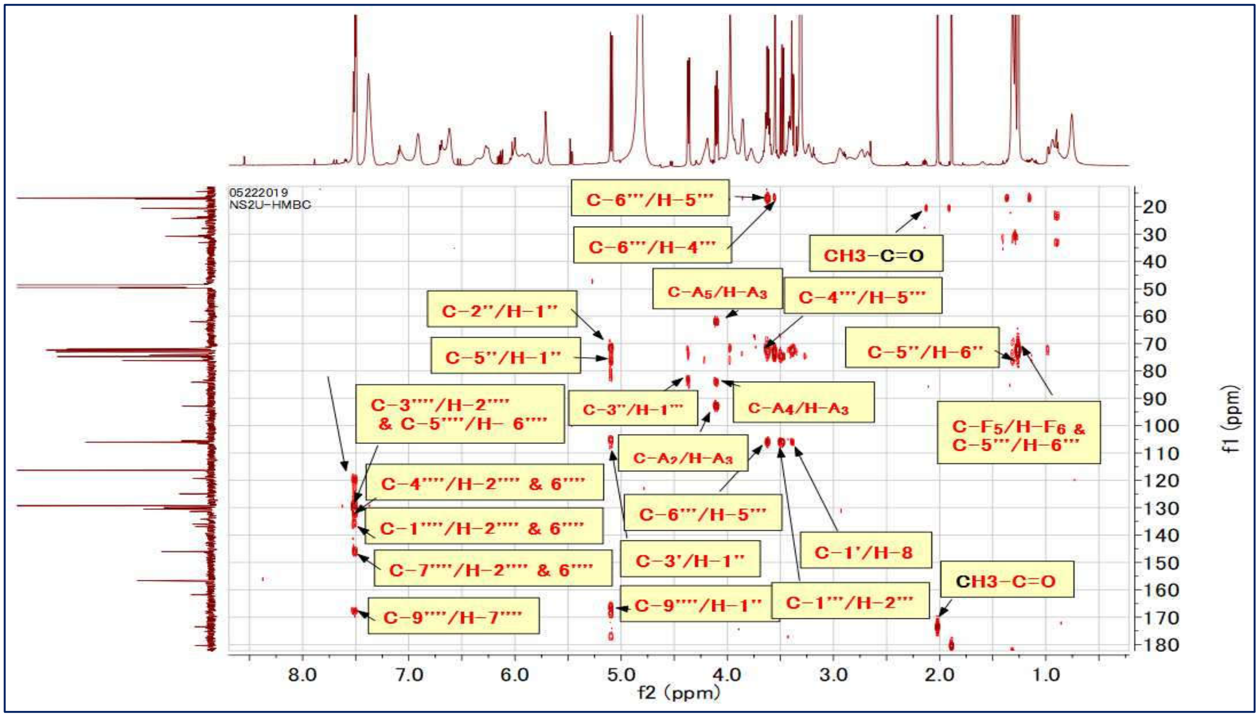

Figure S43: HMBC spectrum of compound 8 (CD3OD).

Compound 9

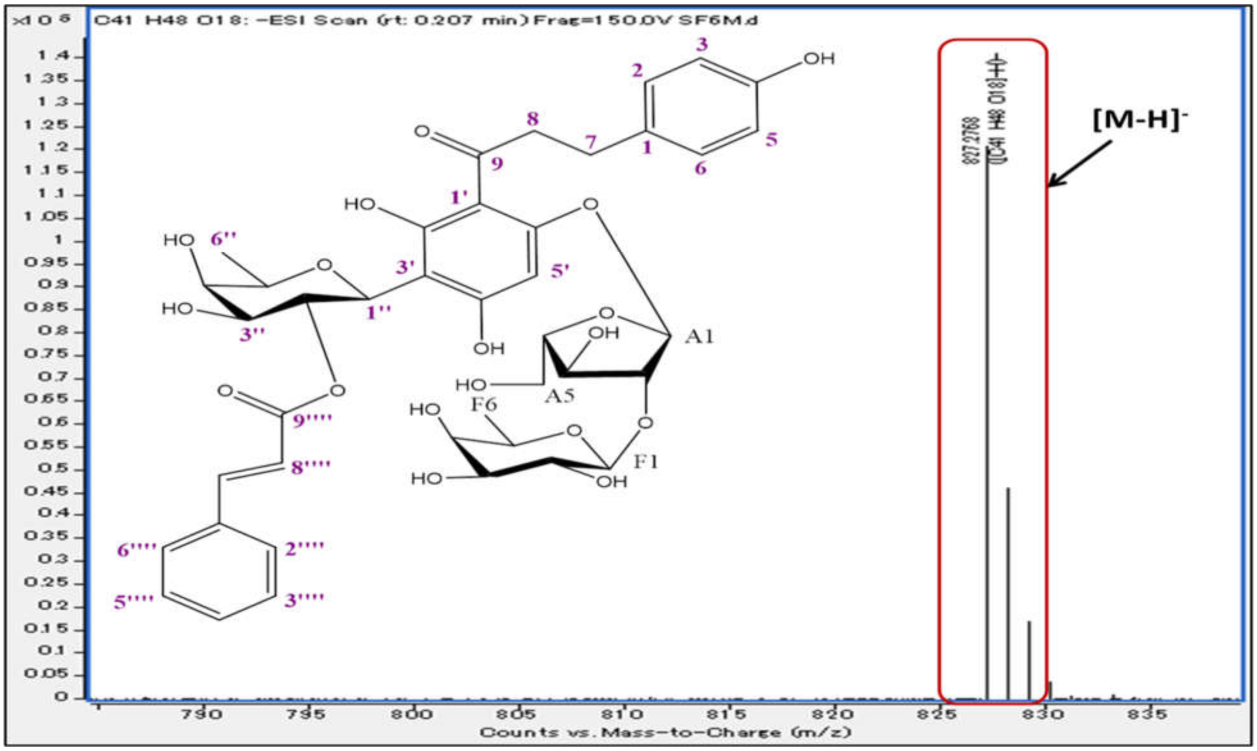

Figure S44: HRESIMS of compound 9

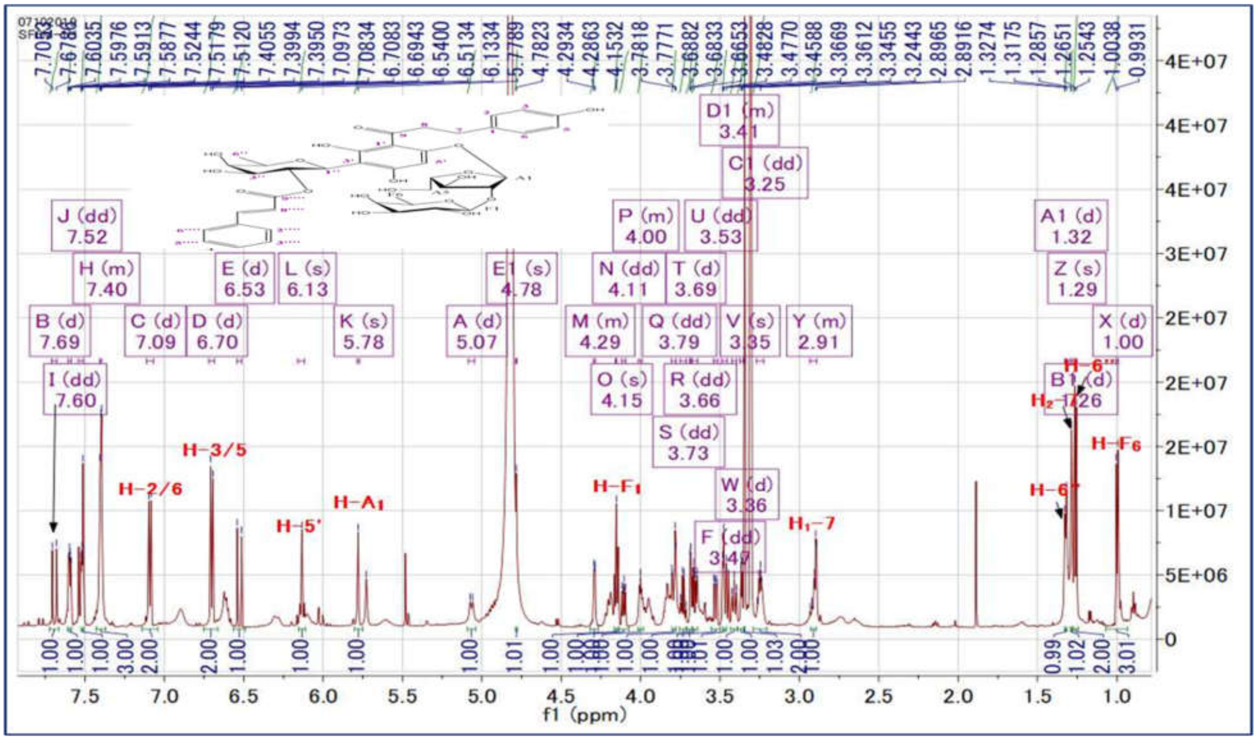

Figure S45: 1 H-NMR spectrum of compound 9 (CD3OD).

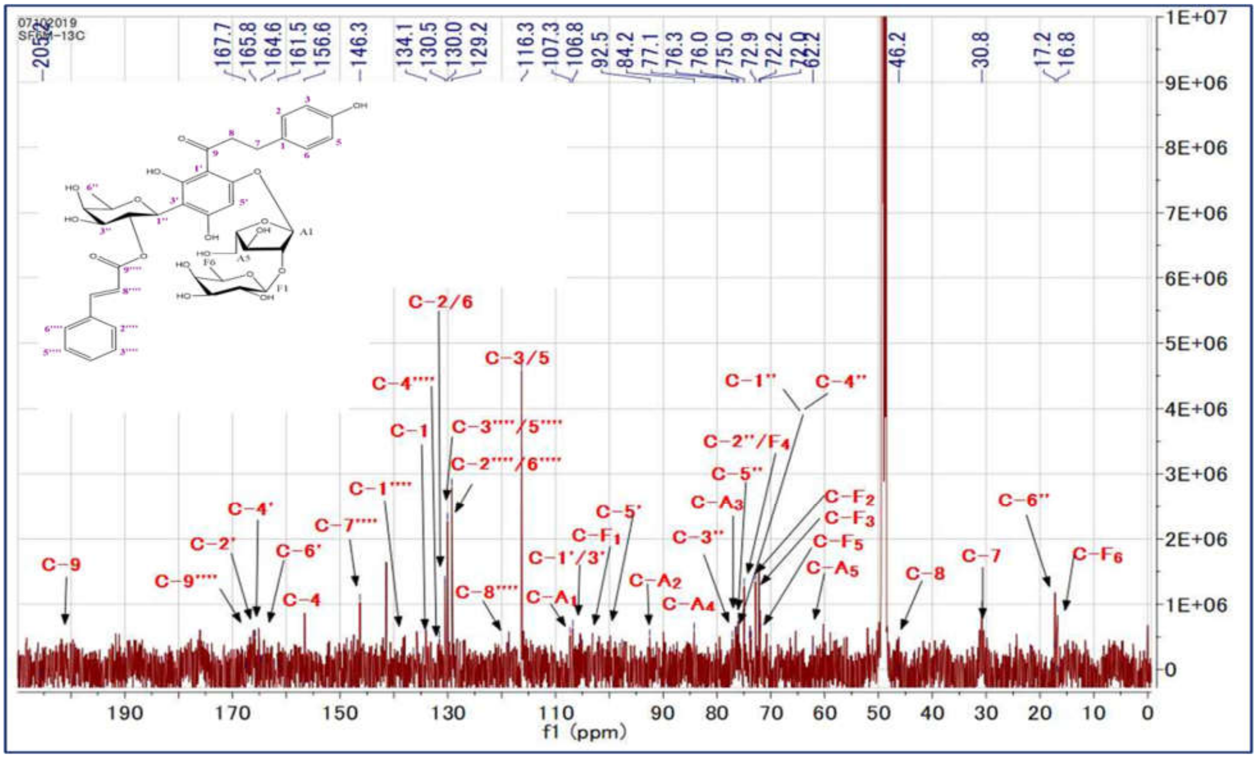

Figure S46: ^{13} C-NMR spectrum of compound 9 (CD_3OD).

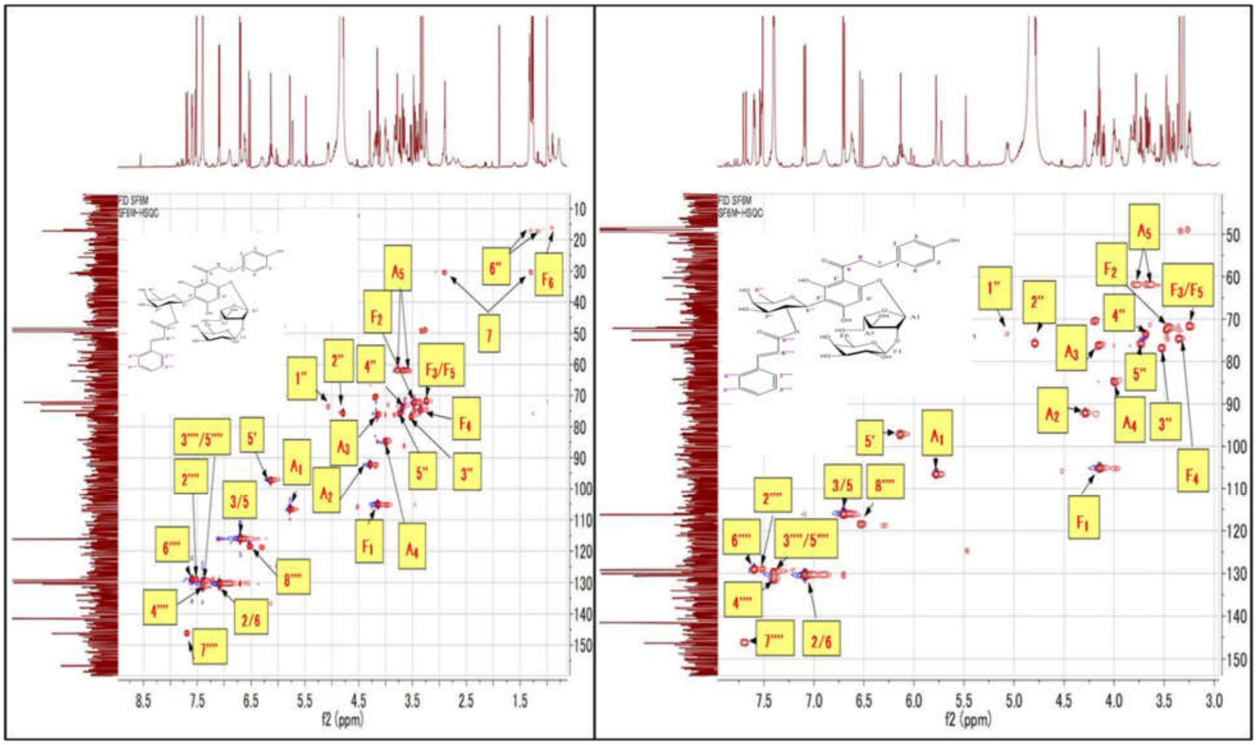

Fig.S47: HSQC spectrum of compound 9 (CD_3OD).

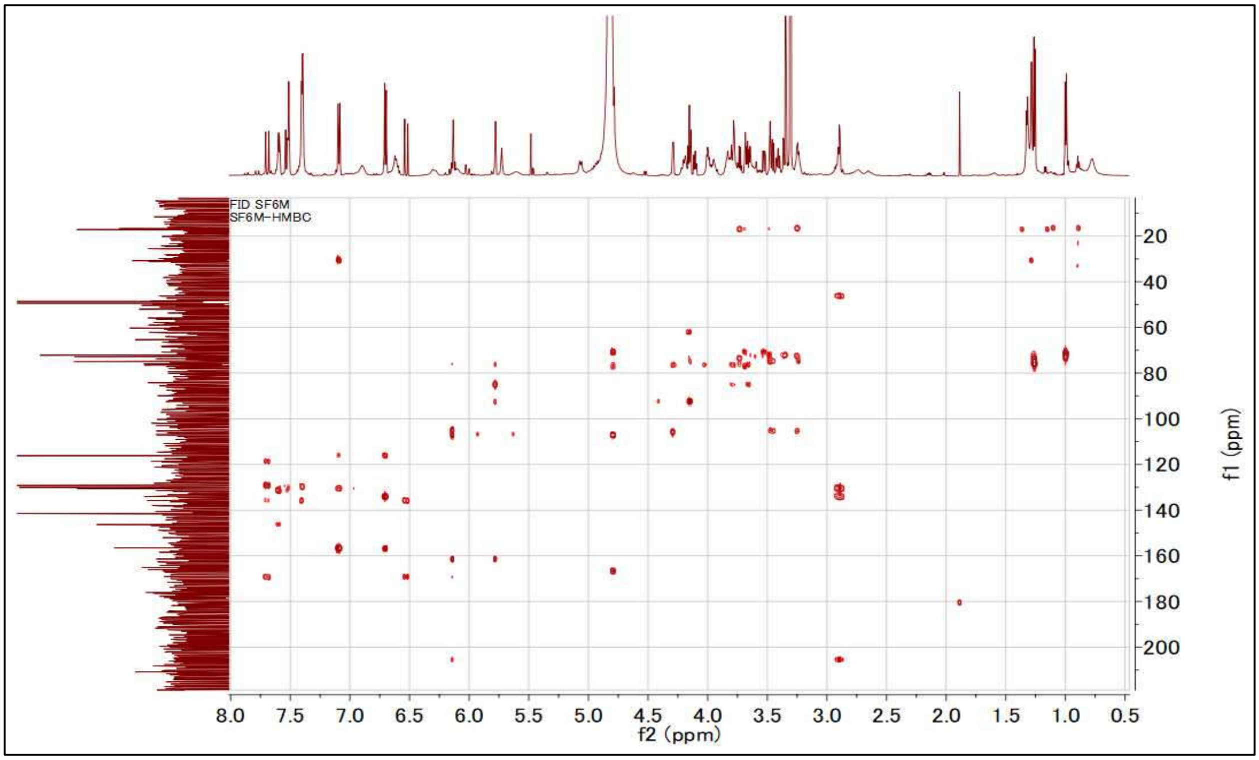

Figure S48: HMBC spectrum of compound 9 (CD_3OD).

Compound 10

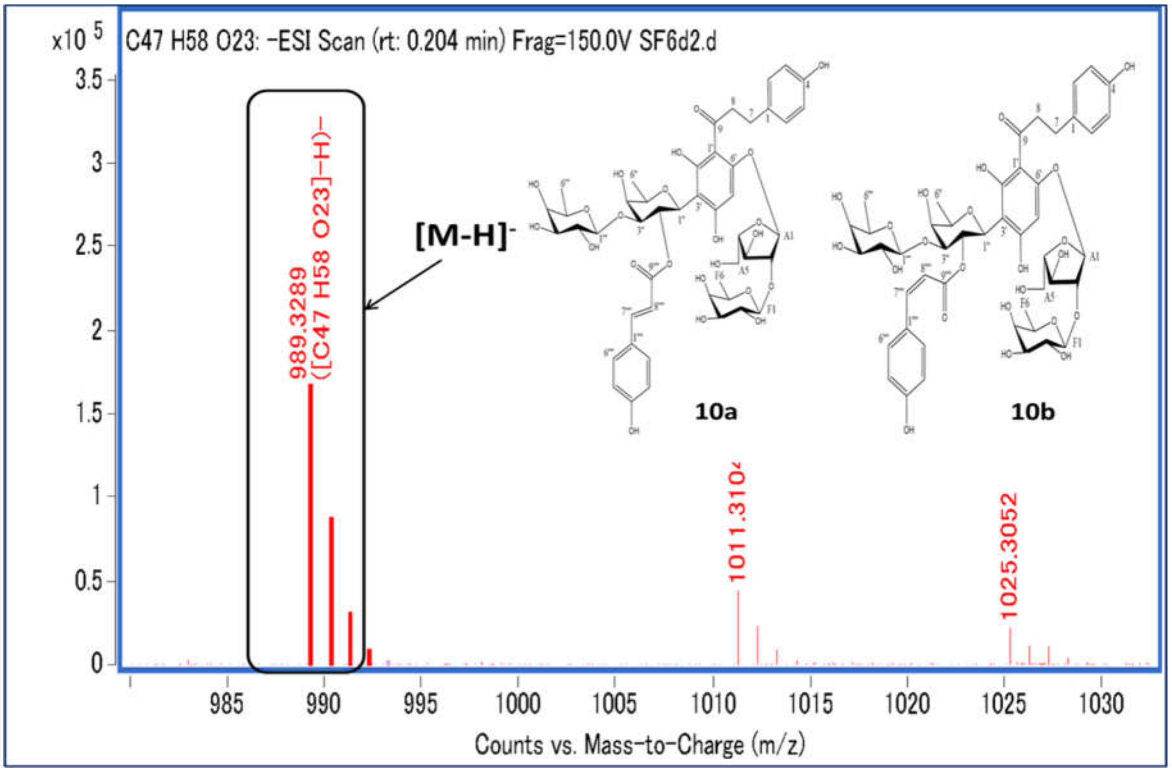

Figure S49: HRESIMS of compound 10

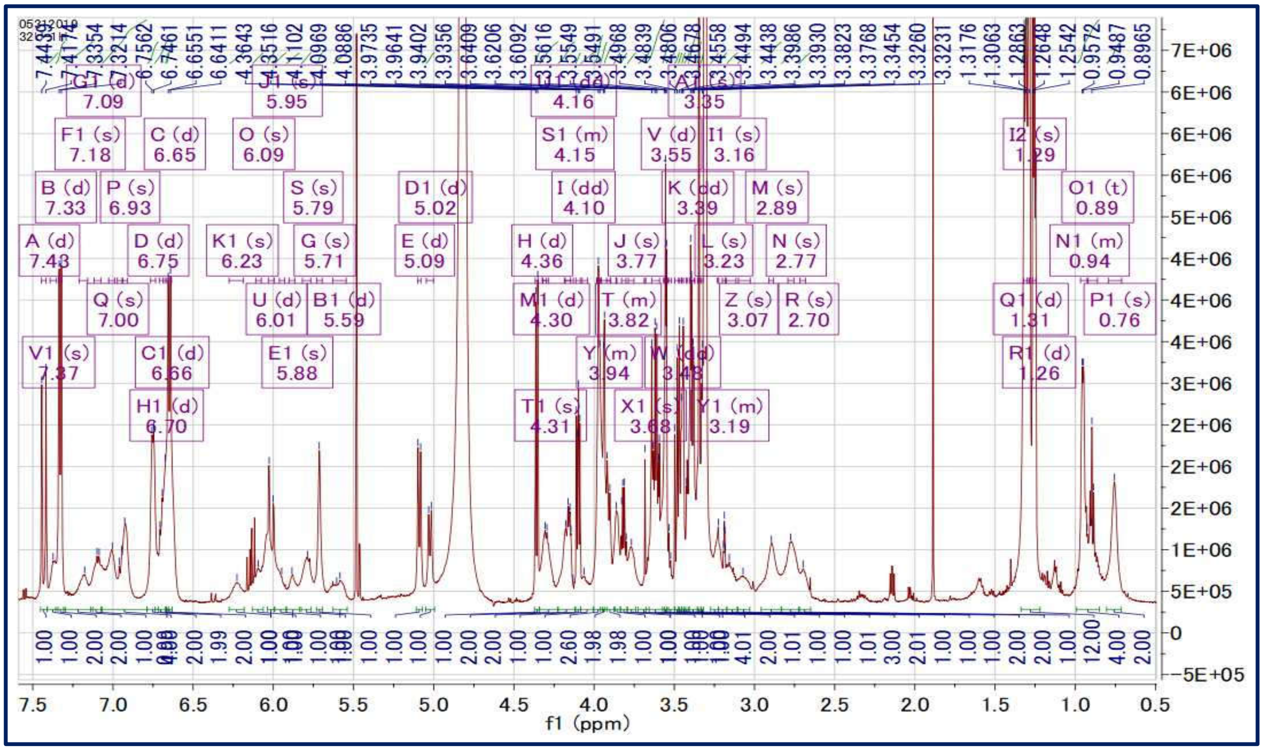

Fig.S50: [1] H-NMR spectrum of compound 10a & 10b (CD_3OD).

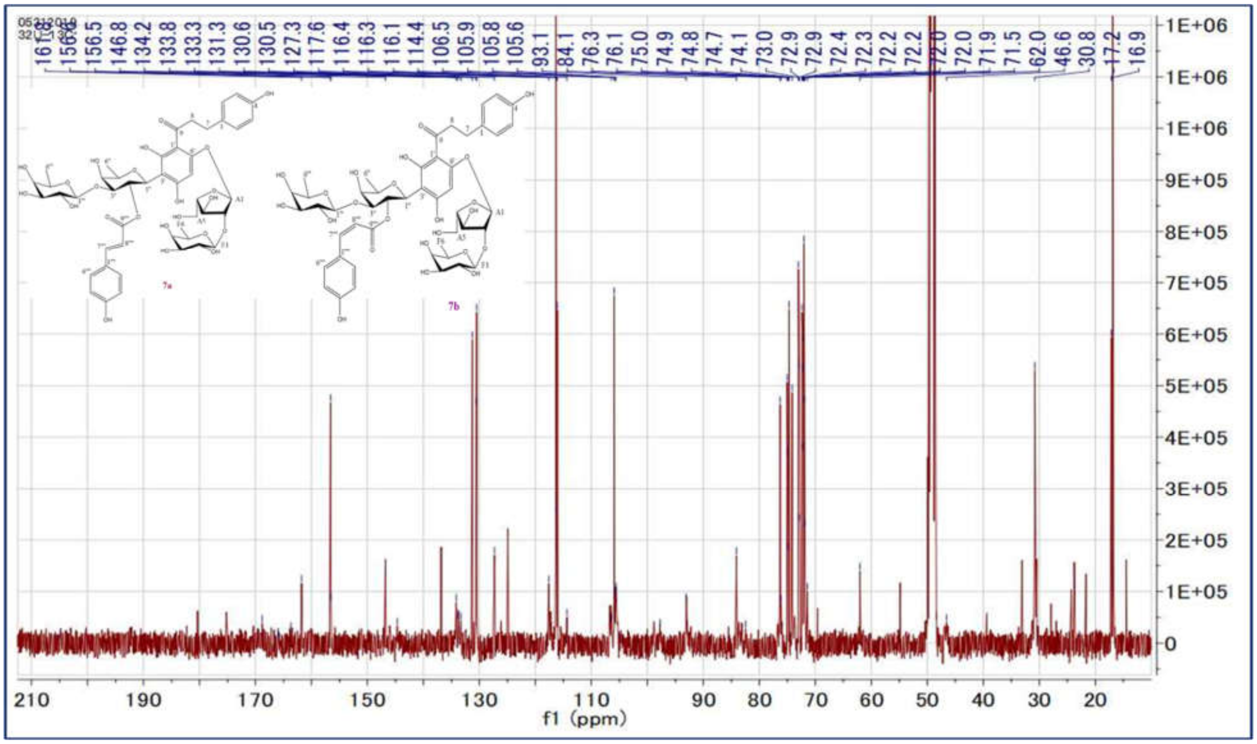

Fig.S51: [13] C-NMR spectrum of compound 10a & 10b (CD_3OD).

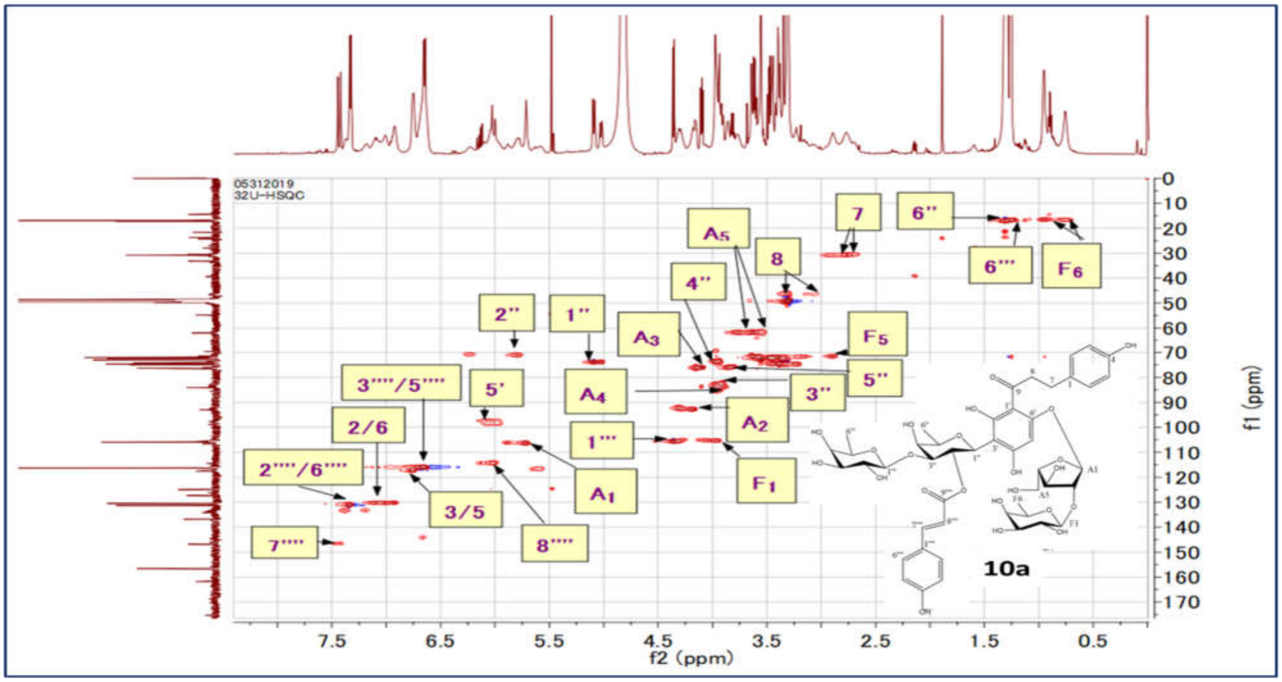

Fig.S52: HSQC spectrum of compound 10a (CD$_3$OD).

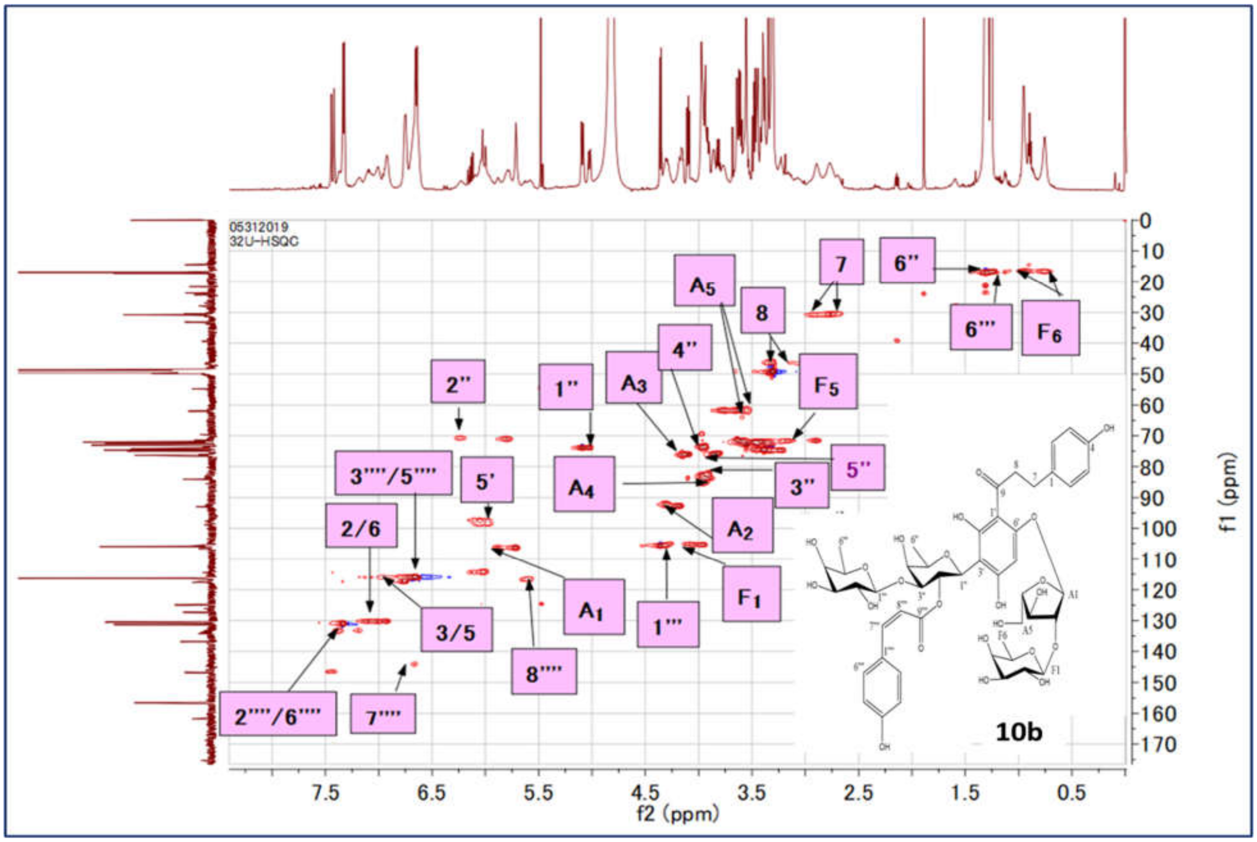

Fig.S53: HSQC spectrum of compound 10b (CD$_3$OD).

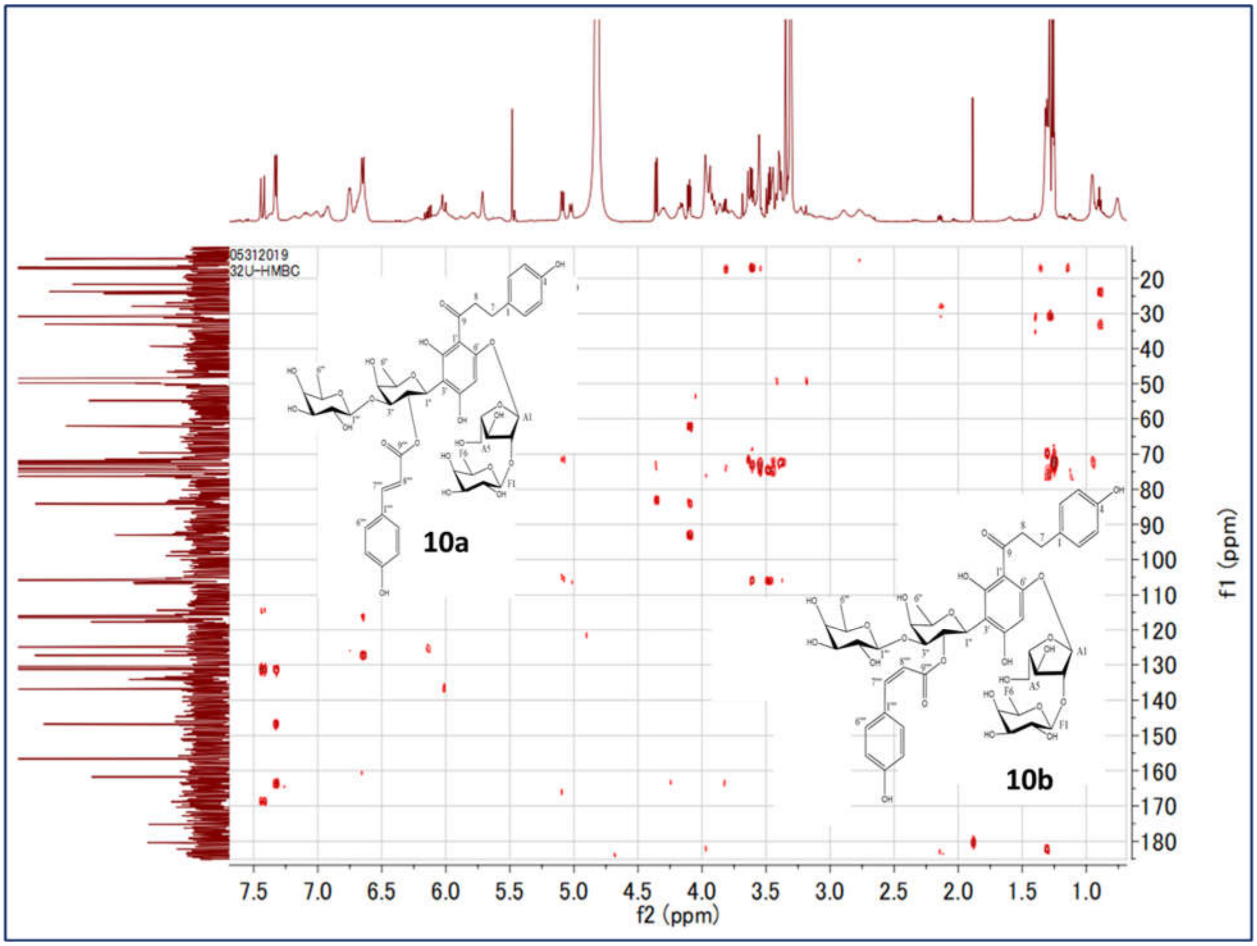

Fig.S54: HMBC spectrum of compounds 10a &10b (CD_3OD).

Compound 11

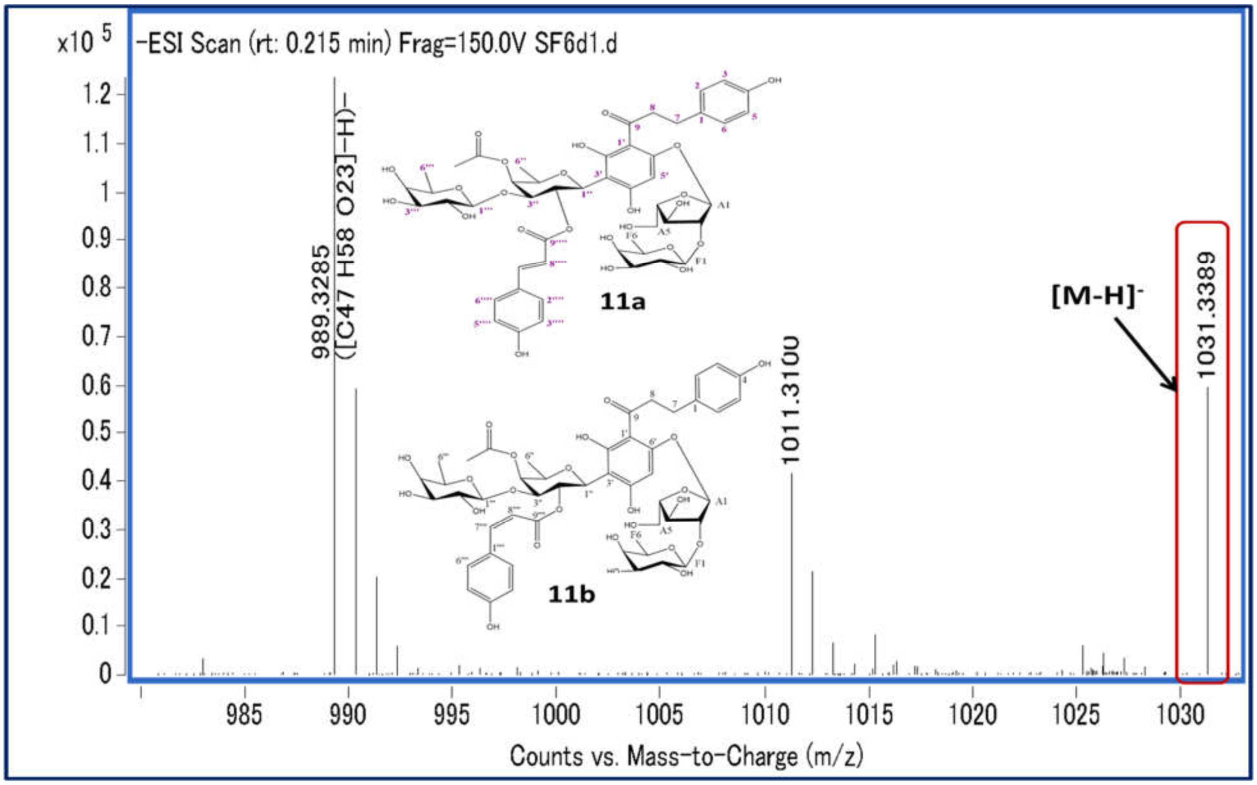

Fig.S55: HRESIMS of compound 11

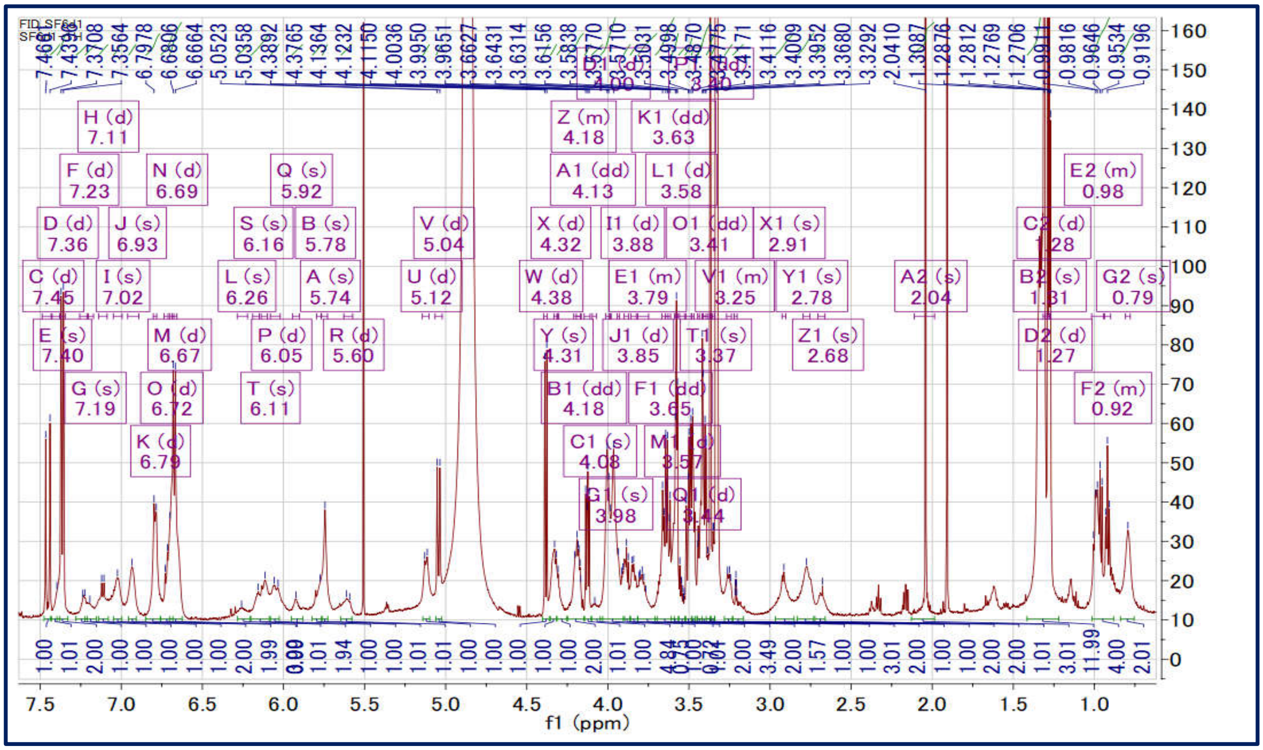

Fig.S56: [1] H-NMR spectrum of compound 11a & 11b (CD_3OD).

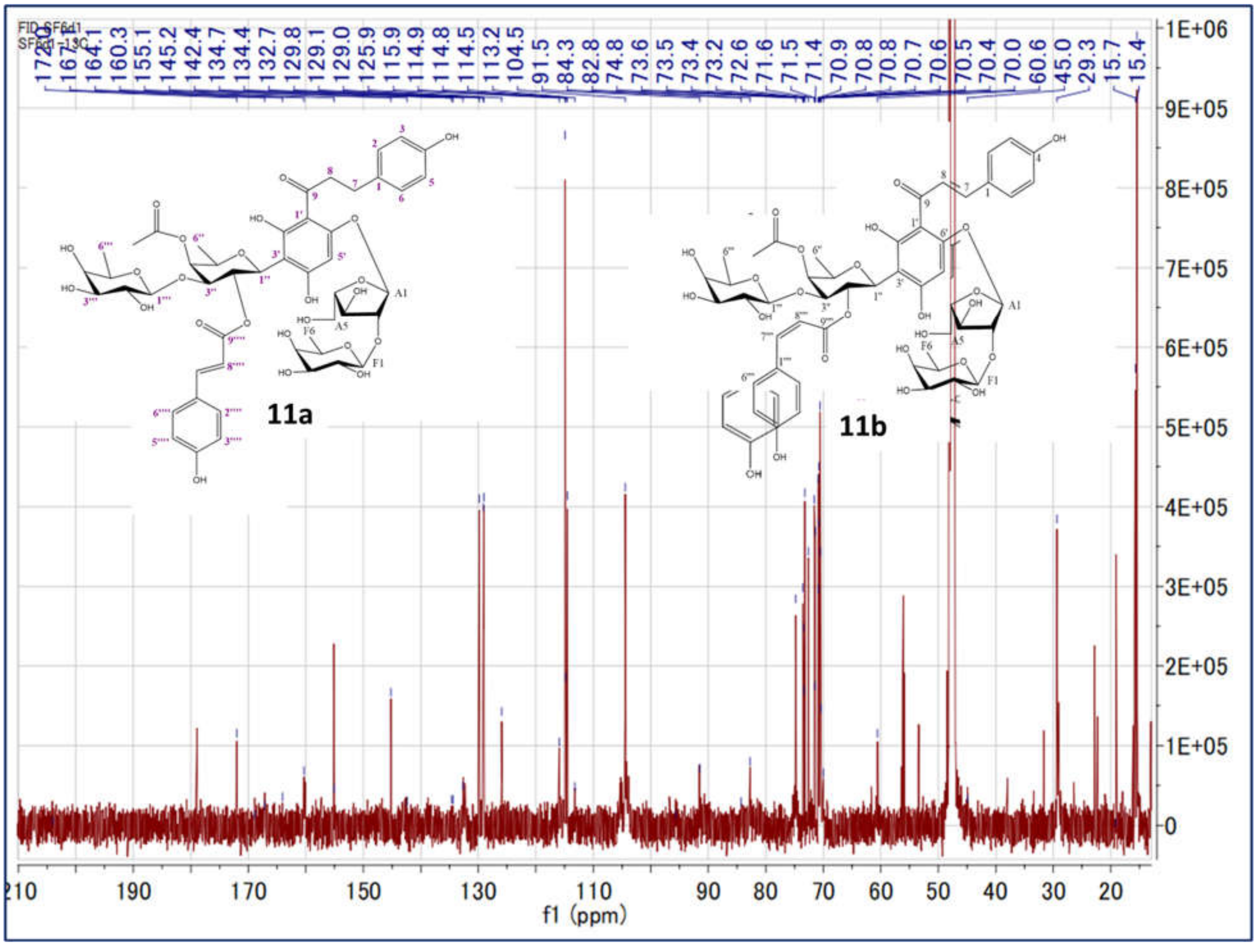

Fig.S57: [13] C-NMR spectrum of compound 11a & 11b (CD_3OD).

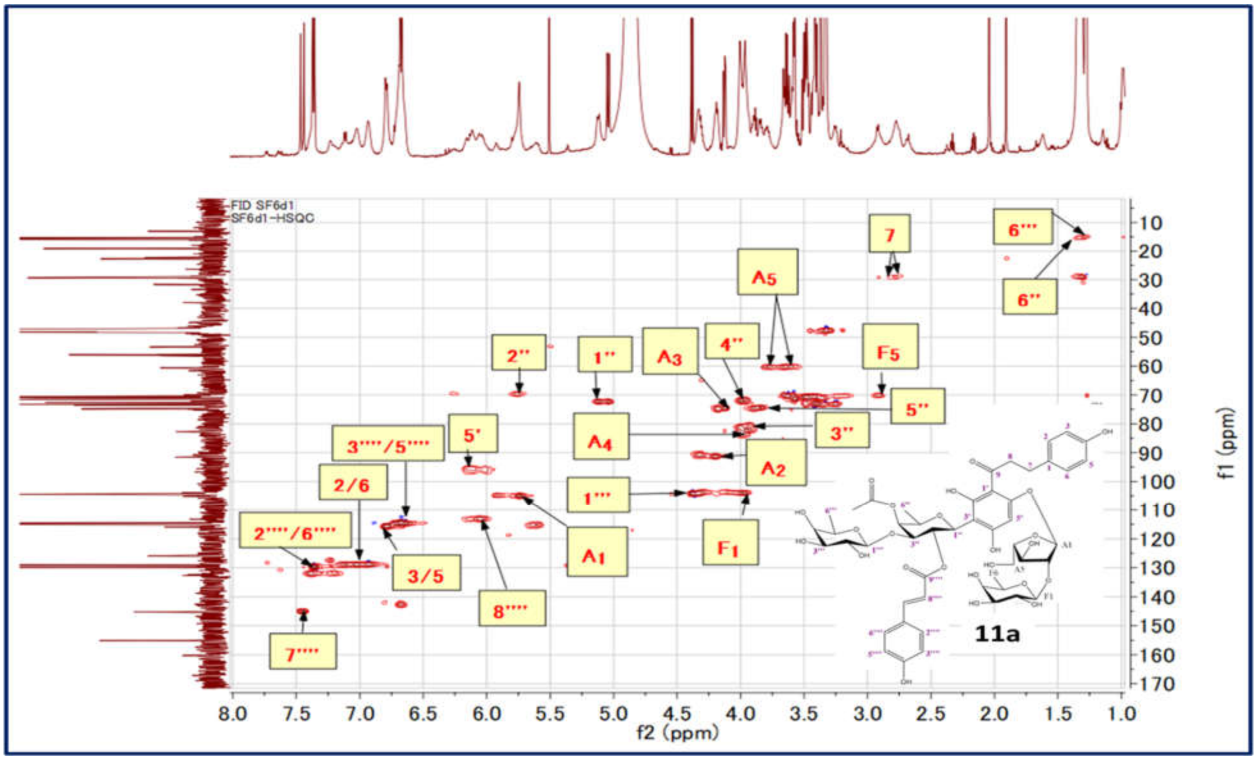

Fig.S58: HSQC spectrum of compound 11a (CD_3OD).

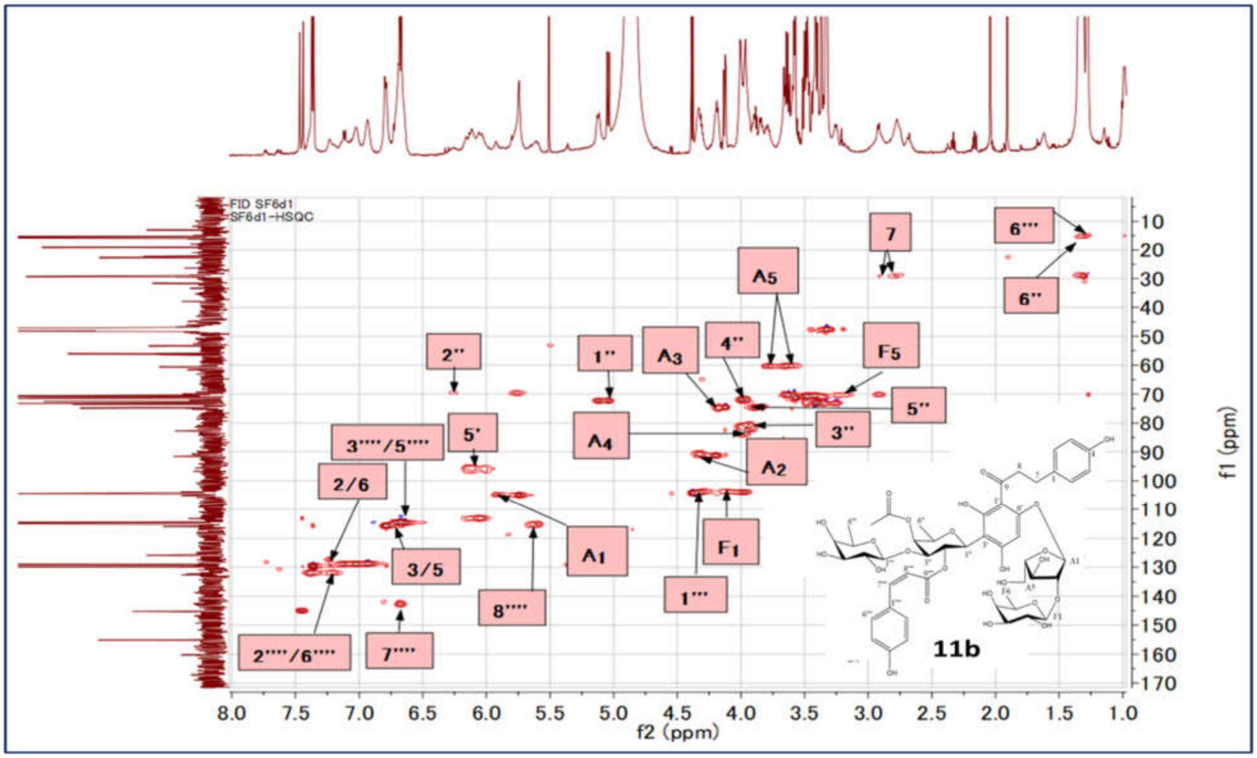

Fig.S59: HSQC spectrum of compound 11b (CD_3OD).

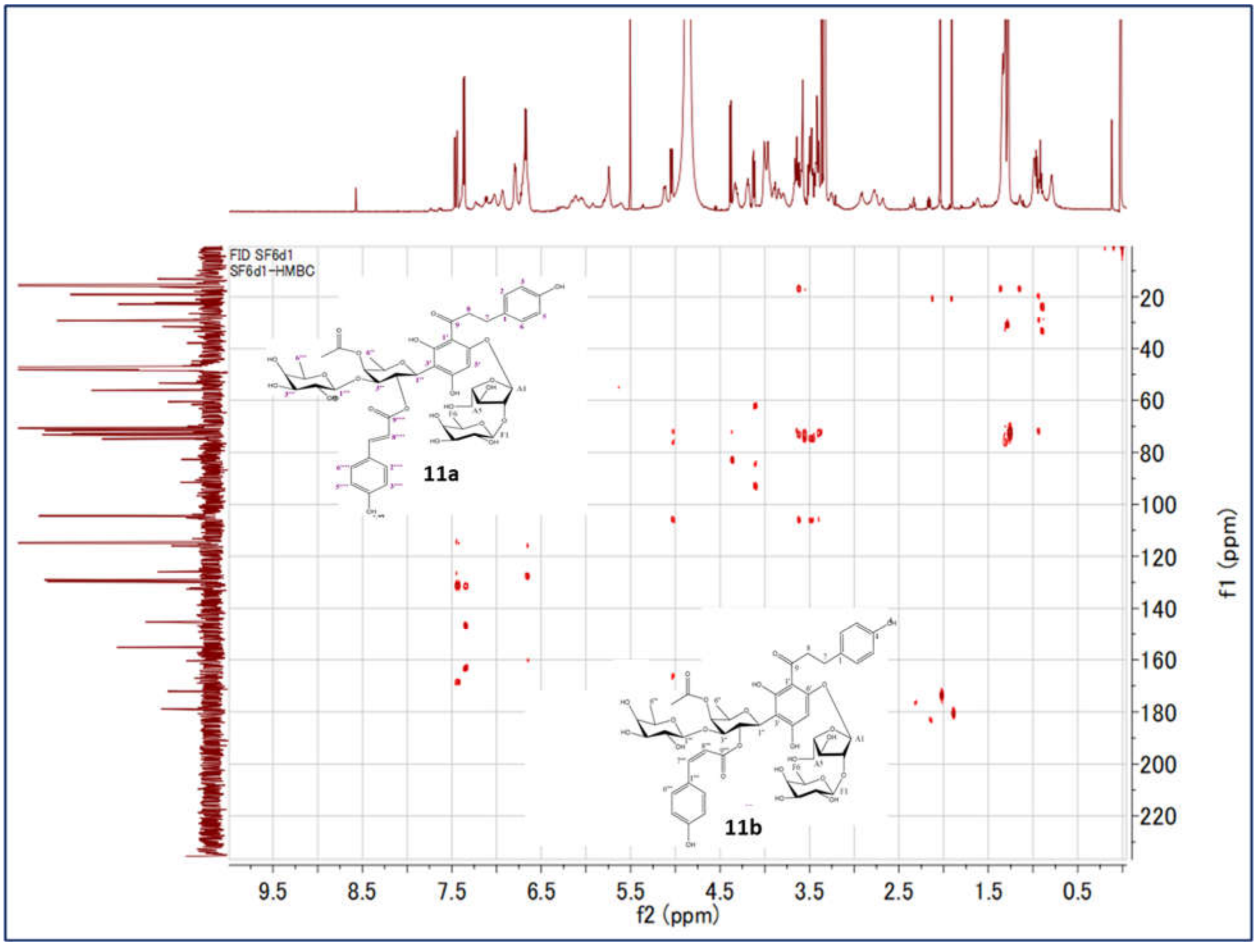

Fig.S60: HMBC spectrum of compounds 11a & 11b (CD_3OD).

Compound 12

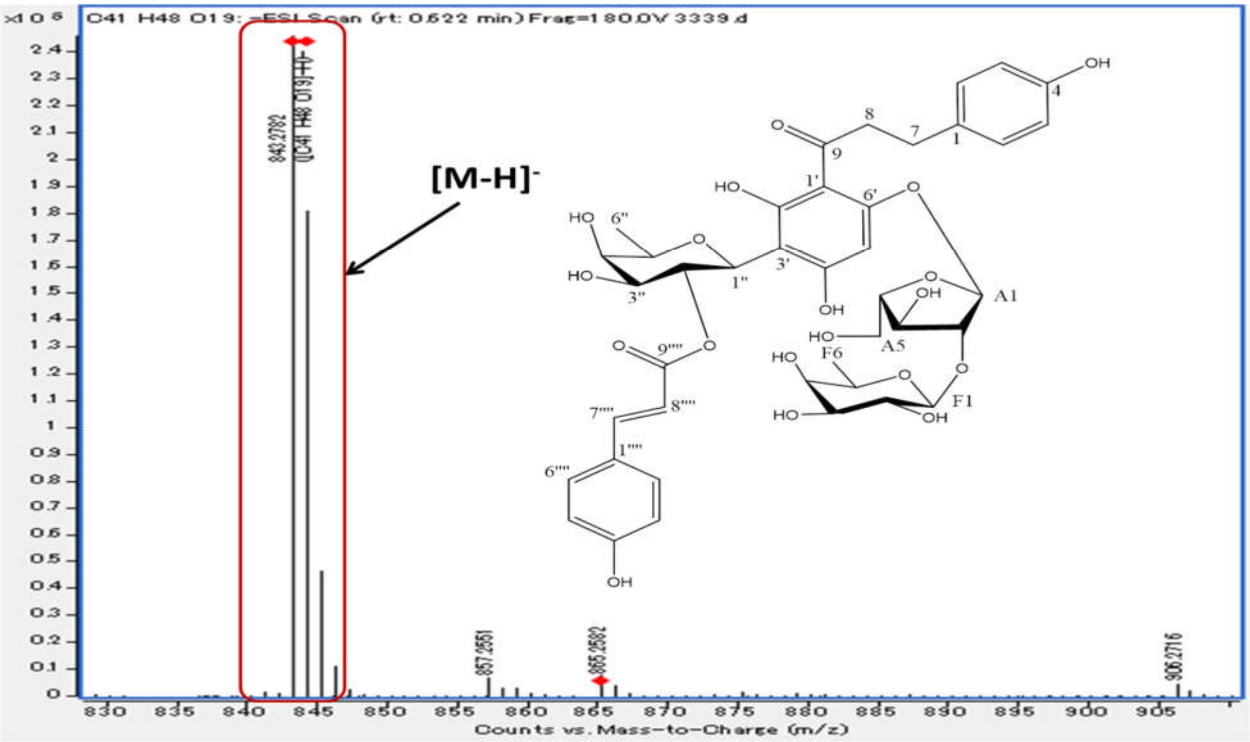

Fig.S61: HRESIMS of compound 12

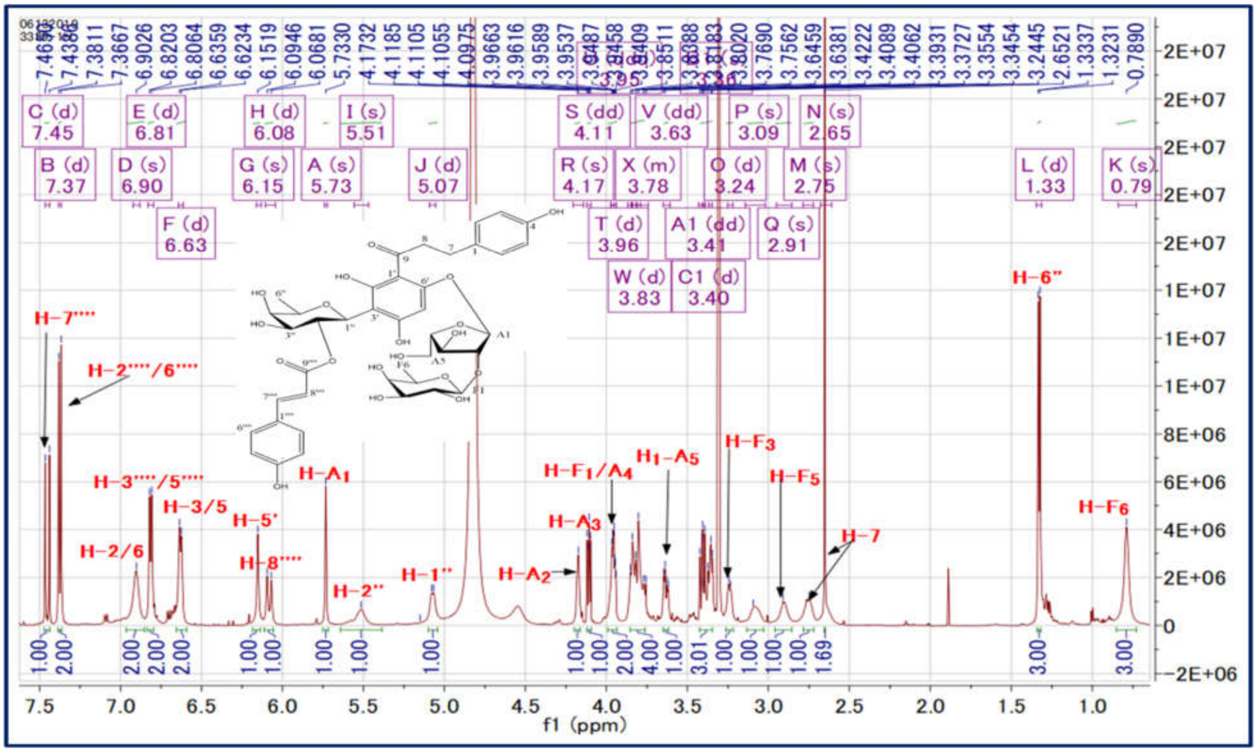

Fig.S62: [1] H-NMR spectrum of compound 12 (CD_3OD).

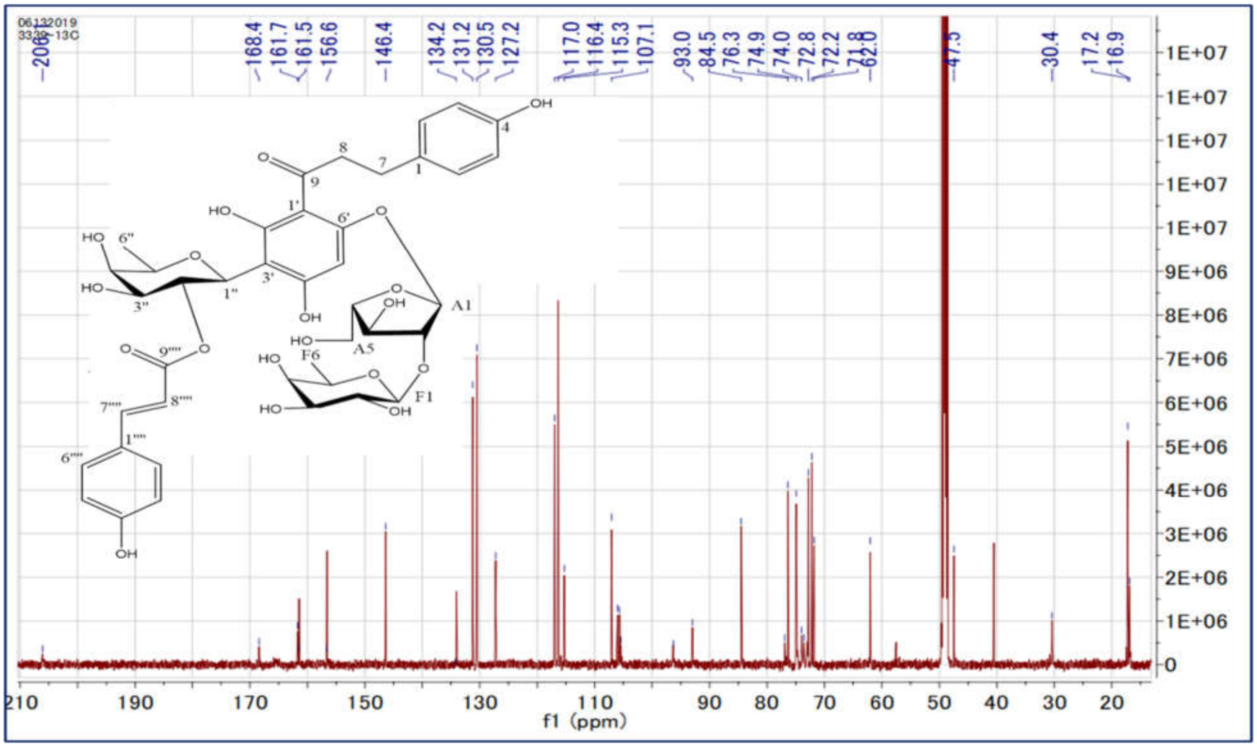

Fig.S63: ^{13} C-NMR spectrum of compound 12 (CD_3OD).

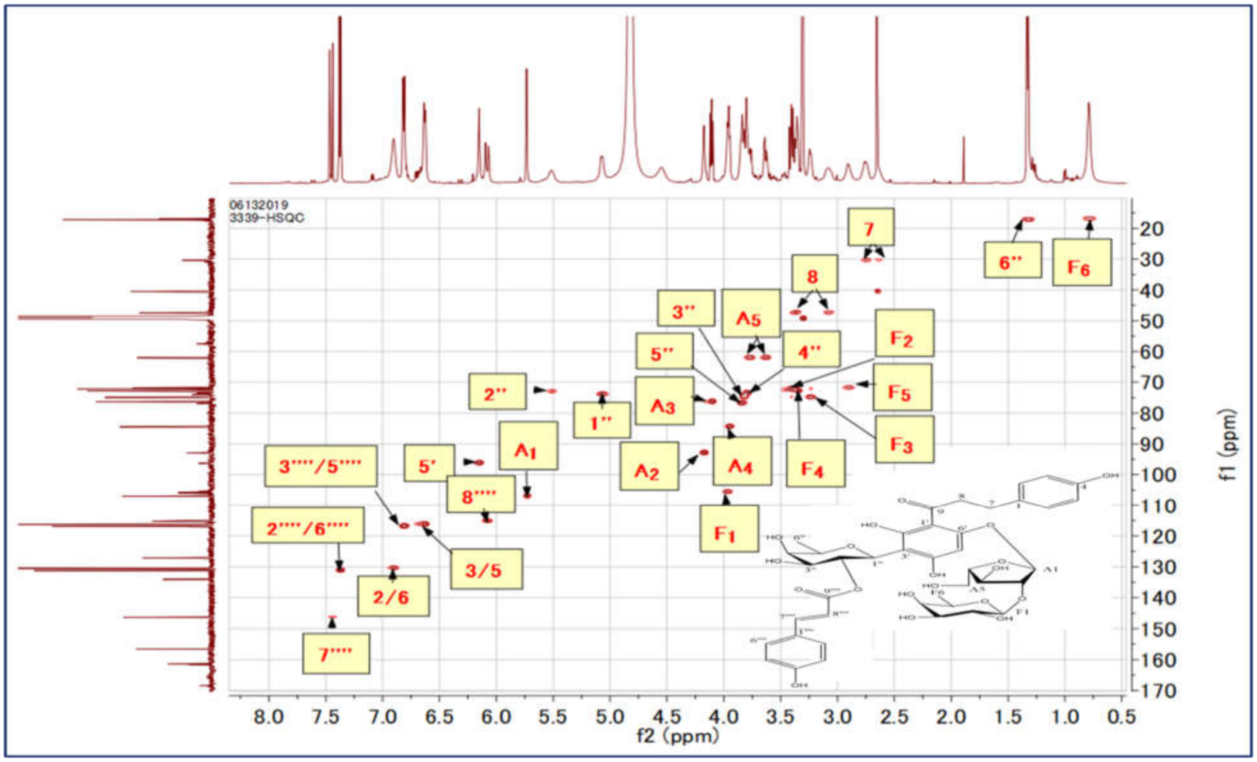

Fig.S64: HSQC spectrum of compound 12 (CD_3OD).

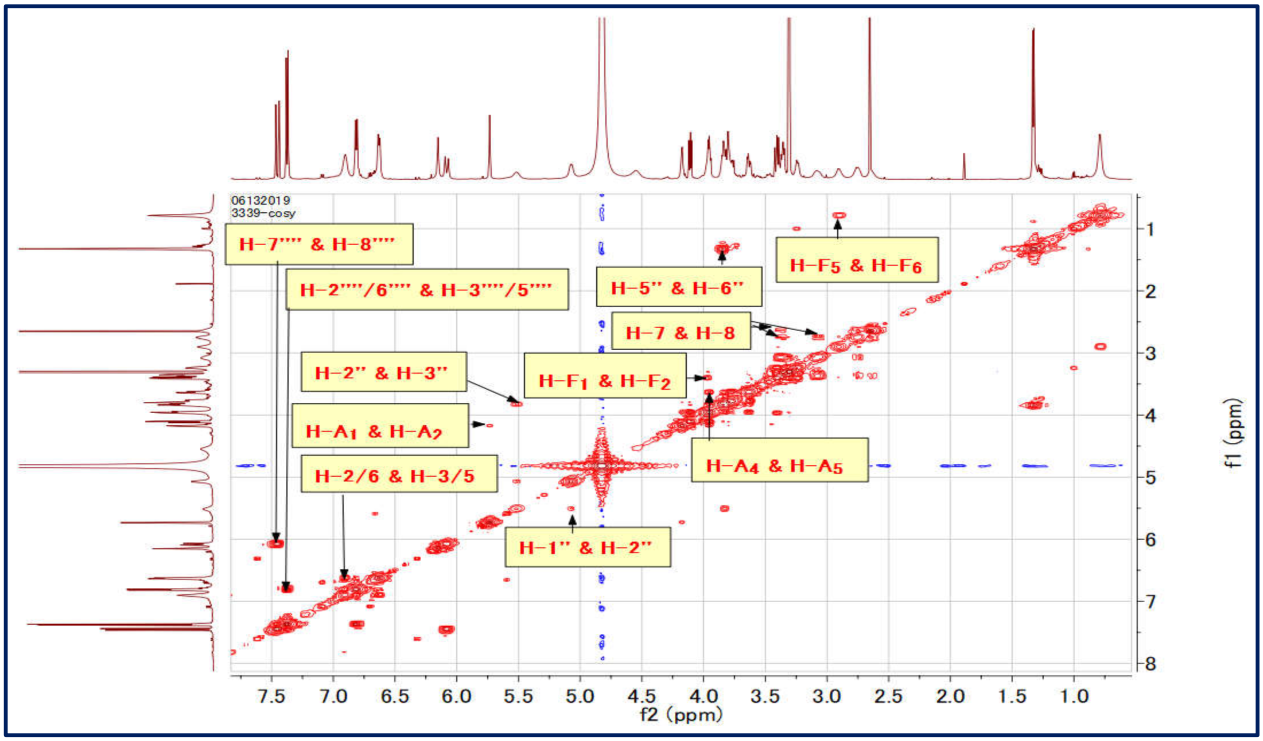

Fig.S65: ^{1}H-^{1}H COSY spectrum of compound 12 (CD_3OD).

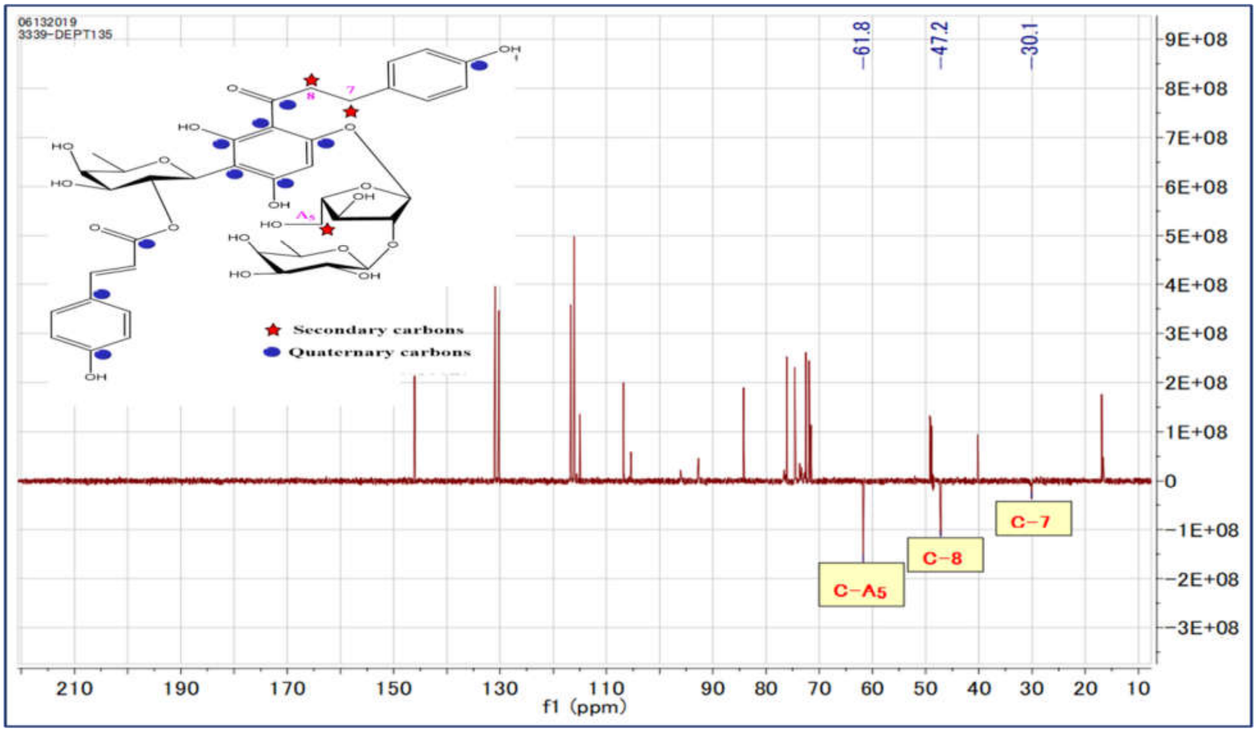

Fig.S66: DEPT-135 spectrum of compound 12 (CD_3OD).

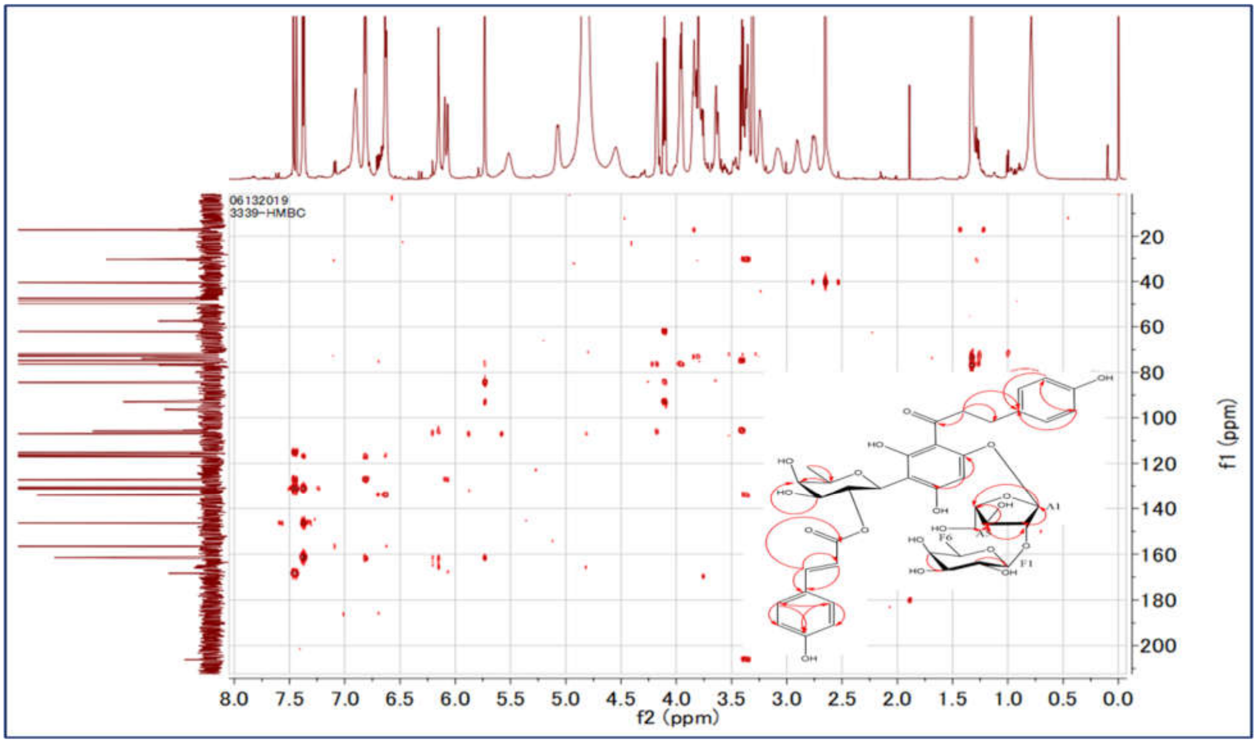

Fig.S67: HMBC spectrum of compound 12 (CD_3OD).

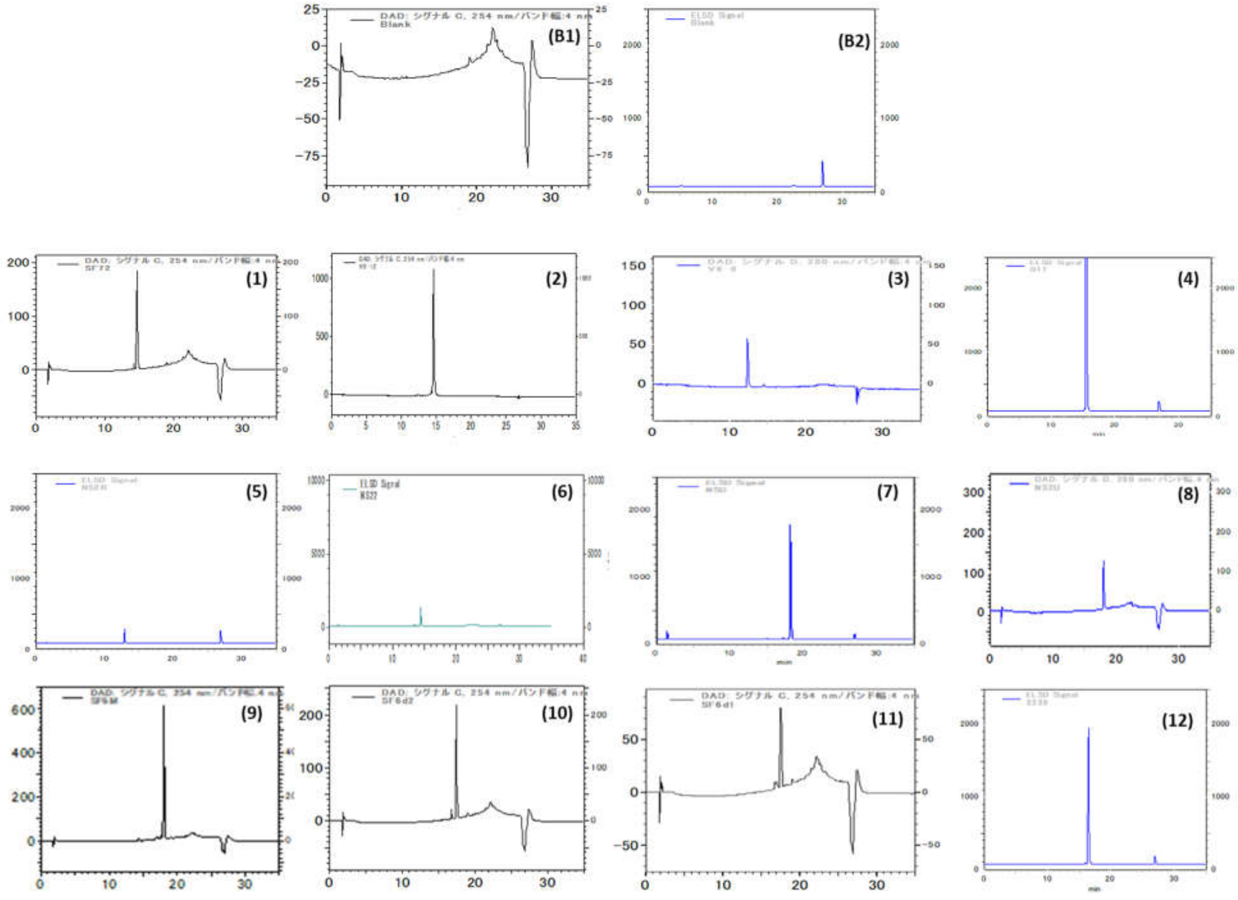

Fig.S68: HPLC purity check for isolated compounds.

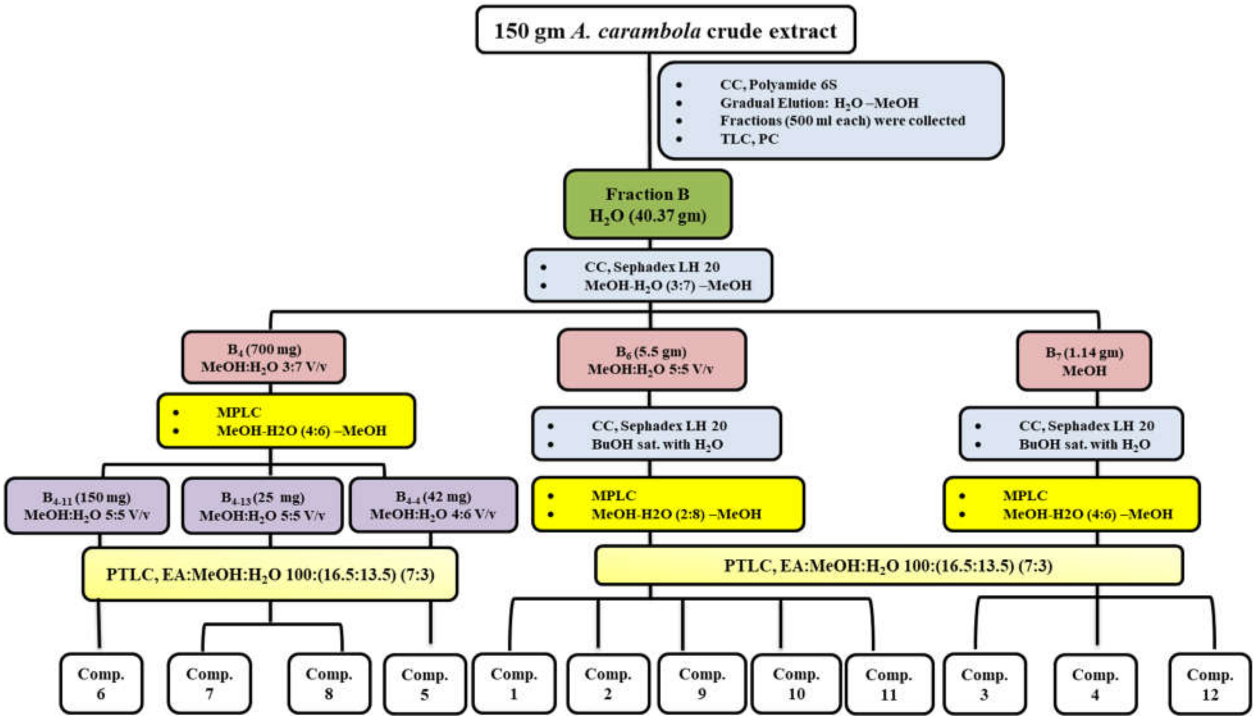

Figure S69 Scheme of isolation of compounds.

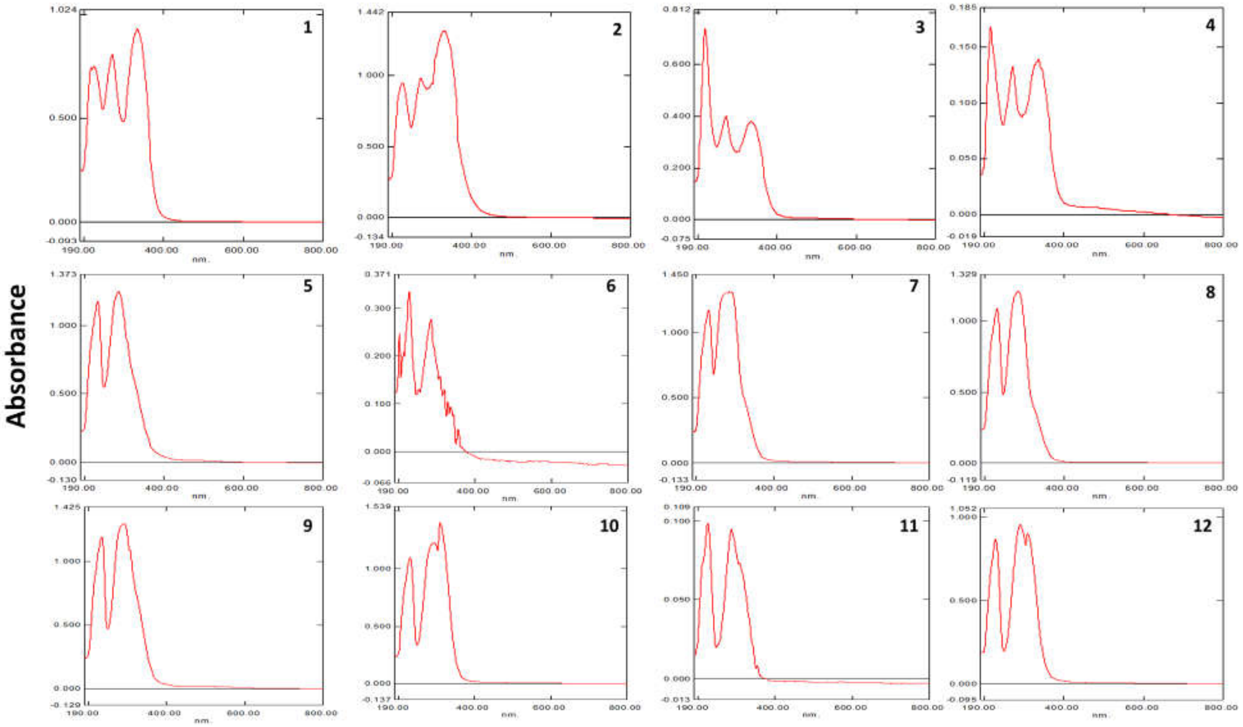

Figure S70 UV spectra of isolated compounds

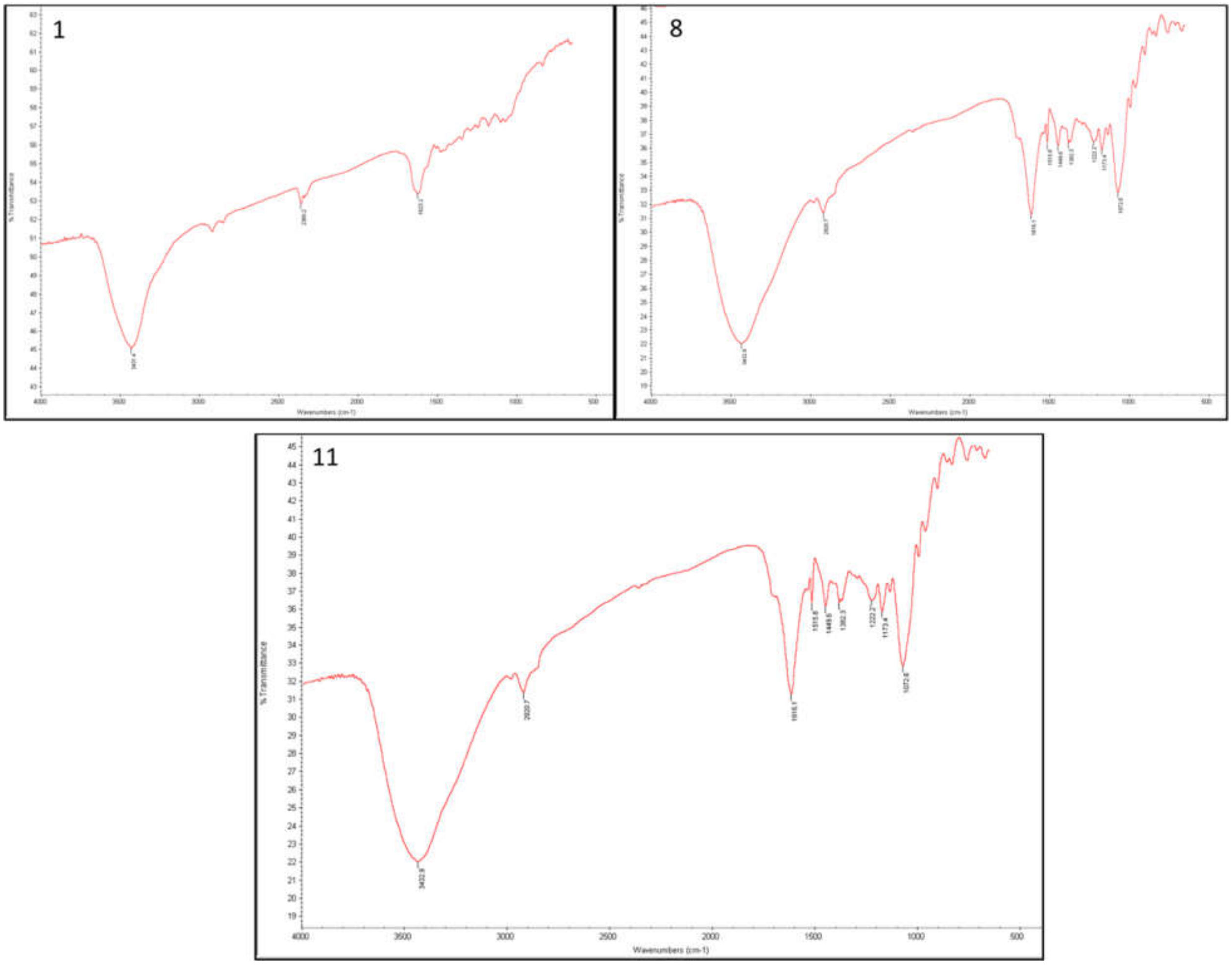

Figure S71 IR spectra of new compounds 1, 8, 11
